# Enzyme kinetics shapes the growth response of metabolic networks

**DOI:** 10.1371/journal.pcbi.1014642

**Published:** 2026-08-19

**Authors:** Leon Seeger, Fernanda Pinheiro, Michael Lässig

**Affiliations:** 1 Institute for Biological Physics, University of Cologne, Cologne, Germany; 2 Computational Biology Research Centre, Human Technopole, Milan, Italy; Institute for Stem Cell Science and Regenerative Medicine, INDIA

## Abstract

Microbes adjust their metabolism to environmental challenges by changing protein expression levels, metabolite concentrations, and reaction rates. Average expression levels in large proteome sectors change coherently, while individual proteins show divergent shifts even within the same pathway. Here, we establish a metabolic model that integrates local enzyme kinetics and global network architecture to predict the joint growth response of proteins and metabolites. Under nutrient limitation, we predict a remarkably simple pattern of proteome reallocation with growth rate: protein expression levels change linearly but heterogeneously. For a given enzyme, the direction of change is determined by its local kinetic constants – catalytic rate and substrate affinity – and by the degree of nutrient restriction affecting its embedding pathway. This double-graded growth response of the proteome is mediated by restriction-dependent metabolite levels, which are predicted to decrease with growth rate in a nonlinear way. The model establishes three specific growth laws: protein expression changes of individual enzymes are negatively correlated with their expression and with their substrate saturation at high growth; average changes of pathways and larger functional sectors are correlated with their internal variance. These predictions are in quantitative agreement with measured system-wide proteomics and metabolomics data of *E. coli*. Enzyme-specific response patterns are a starting point for model-guided interventions into bacterial metabolism.

## Author summary

Engineering microbes, microbial communities, or novel antimicrobials depends on understanding cell metabolism in diverse growth environments. Here, we establish a model of cellular resource allocation in networks of biochemically heterogeneous enzymes and metabolites. We identify key variables that determine resource allocation to an enzyme-substrate pair: its biochemical quality and its position in the metabolic network. Our model predicts quantitative patterns of systems metabolism, explains empirical observations of growth-dependent protein expression, and sets the stage for regulatory and evolutionary analysis of metabolic networks.

## Introduction

Bacteria exhibit remarkable plasticity in response to environmental challenges, adjusting their physiology through changes in protein expression, metabolite concentrations, and reaction rates [1,2]. Understanding and harnessing this plasticity promises advances in medicine, synthetic biology, and biotechnology [3–7]. At the level of metabolic fluxes, this plasticity has been extensively studied using constraint-based approaches [8,9]. These models predict flux states across diverse environments but do not resolve the underlying co-variation of enzyme and metabolite levels. System-wide measurements of the proteome [10–15] and the metabolome [16–19], including several datasets recording proteome changes as a function of growth [10–12,14], are an important step towards this goal.

Empirical models describe important patterns of growth response: large functional sectors, including ribosomal, catabolic, and housekeeping proteins, show coherent and often linear changes under nutrient limitation and other stresses [10,14,20,21]. Akin to flux-balance formulations [8,22–25], sector models assume that enzymes have fixed reaction rates per molecule, neglecting local variations in enzyme saturation and metabolite concentrations [26]. In these models, altered fluxes determine altered expression levels [27], while enzyme-to-enzyme variation within pathways catalyzing the same flux is neglected [28]. Recent work on microbial responses to temperature highlights an alternative perspective: models incorporating metabolite concentrations and enzyme-substrate affinities reveal how local biochemical constraints shape the growth rate [29]. These approaches move beyond sector models by explicitly accounting for enzyme kinetics and metabolite dynamics. Genome-scale kinetic models [7,30] and methods that incorporate kinetic information into constraint-based models [31,32] are another important step in this direction, but the large number of unknown or uncertain kinetic parameters [33–36] currently limits their predictive power [37,38]. Analytical approaches to kinetic models of metabolism have revealed generic patterns that generalize across parameterizations [39–43], including mathematical properties of optimal resource allocation [44,45] and scaling relations between individual enzyme and substrate concentrations [46], all of which enable an efficient computation of optimal resource allocation in kinetic models. Recent conceptual syntheses of kinetic modeling with constraint-based [32] and analytical approaches [46,47] unify the modeling of fluxes, proteome and metabolome resource allocation, yet leave enzyme-specific, heterogeneous growth responses insufficiently explained.

The present paper addresses this gap. We develop an analytically tractable multi-level model for metabolic networks that integrates local enzyme kinetics with global network architecture graded in pathways and larger functional sectors. Building on enzyme cost minimization [48] and balance conditions from growth balance analysis [44,45], we derive a simple but general principle linking metabolite concentrations and enzyme levels to environment-dependent nutrient restriction factors. This principle predicts specific linear, yet heterogeneous patterns of growth response for individual enzymes, pathways, and functional network sectors. We validate these predictions using quantitative proteomics, metabolomics, and enzyme kinetics data, showing that our model captures key, experimentally observable features of cellular resource allocation.

## Results

### Proteins respond linearly but heterogeneously to growth limitation

**Fig 1:**
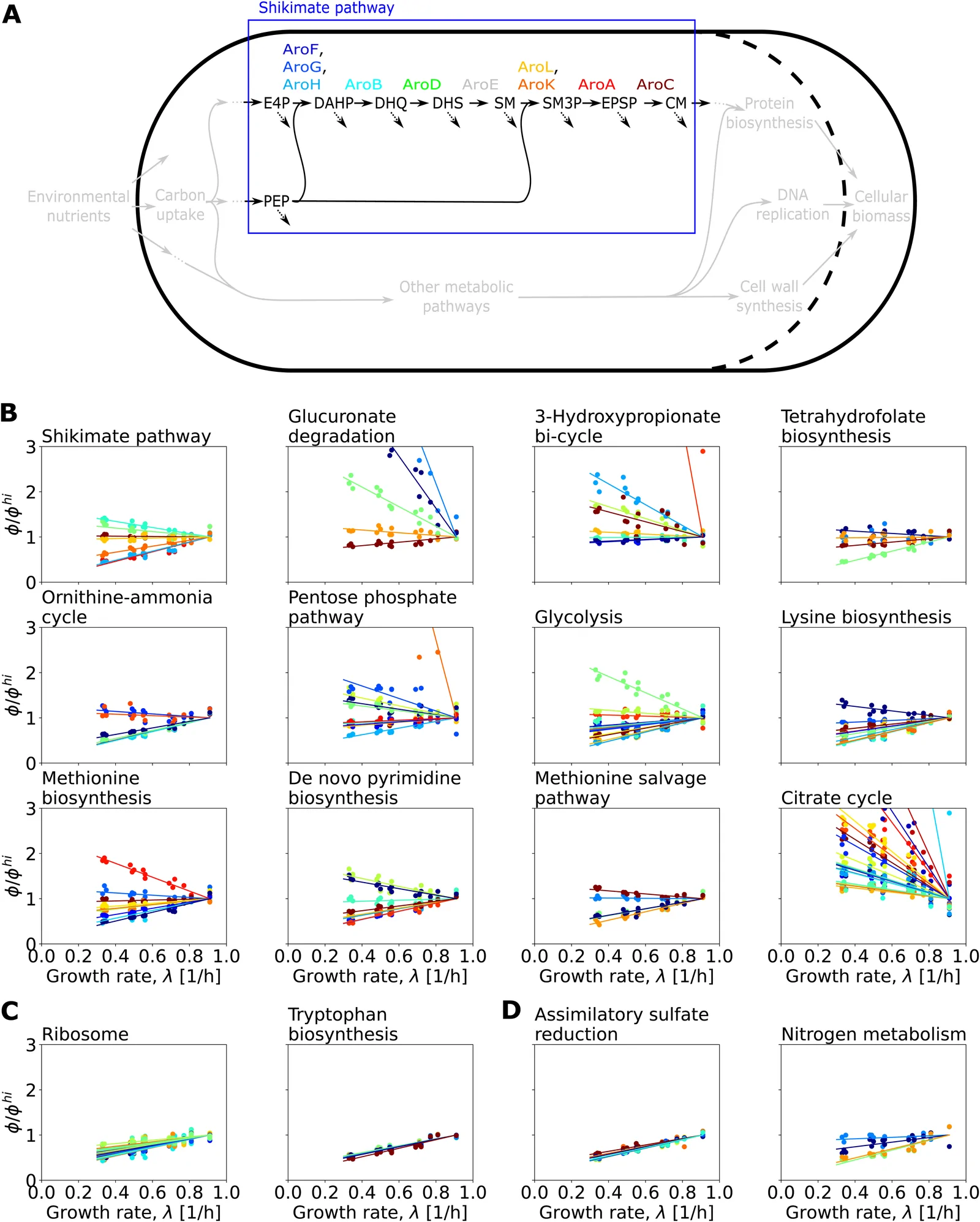
Enzyme-specific proteome growth response under nutrient limitation in *E. coli.* **(A)** Example of a metabolic pathway (Shikimate pathway, blue box) embedded in a metabolic network. The schematic illustrates the main organic metabolites (black) and enzymes (colored, without ATP and NADH). The model evaluates growth-optimal metabolite concentrations, enzyme levels and mass fluxes (arrows), accounting for loss of metabolites through dilution by growth at each reaction step. **(B-D)** Empirical growth response factors, $\hat{Q}$ (dots), are plotted at different growth rates relative to a high-growth state, $\lambda /\lambda^{hi}$, for a set of enzymes in different pathways (listed in S1 Table). These data are shown together with linear fit lines for each enzyme, which define the values $\phi^{hi}$ (at $\lambda /\lambda^{hi}=1$) and $\phi^{lo}$ (at $\lambda /\lambda^{hi}=0$), and the empirical growth response parameter $\hat{q}\equiv \phi^{lo}/\phi^{hi}$ (see text). **(B)** Pathways with heterogeneous growth response are compared to co-regulated (C) or nutrient-replete pathways (D) with homogeneous growth response (see text). The full set of metabolic pathways is shown in S1 Fig. Proteomics data from Mori et al. [14]; pathways are KEGG modules [49] for which ≥5 proteins were detected under all carbon limitation conditions in the dataset.

We analyze absolute proteome fractions for 1,647 *E. coli* proteins under graded inhibition of glucose uptake [14]. We assign enzymes to KEGG modules [49] and curate 34 pathways that meet two criteria: each pathway contains ≥5 proteins quantified in all conditions, each pair overlaps by < 50%. An example is shown in Fig 1A; all curated pathways and proteins are listed in S1 Table.

Two remarkable patterns characterize the proteome growth response to carbon limitation in this set of pathways. First, most enzymes change expression approximately linearly with growth rate (Figs 1B, 1C and S1). Statistical analyses of these data do not support constant and quadratic dependencies by comparison of fit-residuals to residuals between biological replicates (*p* < 10^−25^ and *p* < 10^−10^, respectively). Linear growth response appears over a wide range of growth rates ($0.3≲\lambda /\lambda^{hi}<1$; the superscript hi denotes a nutrient-rich reference state). In this range, deviations from the linear fits do not differ significantly from deviations between biological replicates (p≈0.2, Kolmogorov-Smirnov test, Methods). Second, growth response is strongly heterogeneous: in most pathways, expression changes have different amplitudes and different signs (Fig 1B). In a given growth condition, we assume that enzymes in the same pathway typically generate similar metabolic flux, because they contribute to the same metabolic function. Therefore, the observation of intra-pathway heterogeneity contrasts with previous models that explain growth responses by changes in catalyzed fluxes [50].

### A Michaelis-Menten reaction network predicts the joint response of proteome and metabolome

To explain why proteins show linear yet heterogeneous growth responses under nutrient limitation, we set up a solvable computational model of growth-dependent systems metabolism. Basic building blocks are metabolic pathways, modeled as sequences of enzymatic reactions obeying Michaelis-Menten kinetics [29,40]. These pathways are treated as units of nutrient constraint propagation. In a first step, we determine the growth-dependent intra-pathway resource allocation under a given input constraint. Here, Michaelis-Menten kinetics serves as a mathematically tractable approximation to more complex and partially reversible reaction kinetics. This approximation is motivated for fast-growing microbes in S1 Appendix, allows for the systems-level integration of kinetic data [16,51], and is validated by our data analysis presented below. The flux of a given metabolic step *i* is determined by the enzyme concentration $\phi_{i}$, the substrate concentration $\rho_{i}$, the catalytic constant $\kappa_{i}$, and the substrate affinities $K_{i}^{-1}$. We assume a constant intracellular protein density [1,13,52–54]; all concentrations and fluxes are expressed in units of this density (Methods). Production of protein biomass leads to cell growth, which dilutes proteins and metabolites [40,44,45,55–57]. Similar modeling frameworks have been used to study optimal resource allocation to the ribosome [40] and between metabolites and enzymes [44,46], as well as to predict dynamic growth response to temperature changes [29]. We compute growth-optimal pathway states that maximize the mass production rate per unit of protein mass, using enzyme cost minimization [48] in balanced exponential growth [58]. We solve the resulting balance equations [44] analytically; derivations are given in Methods and S1 Appendix.

In a second modeling step, we determine the inter-pathway resource allocation by a self-consistent closure. Together, the model provides an analytical map from input constraints to growth-optimal downstream metabolite concentrations, enzyme concentrations, and mass fluxes for a network under nutrient restriction (Fig 1A).

Growth-optimal metabolite concentrations emerge from balancing loss by dilution against suboptimal saturation of the consuming enzymes. Hence, the optimal concentration $\rho_{i}$ depends on three main factors: network position, nutrient level, and reaction kinetics. Specifically, higher $K_{i}$ lowers enzyme efficiency and increases $\rho_{i}$, while higher $\kappa_{i}$ lowers the amount of enzyme required to balance low saturation and reduces $\rho_{i}$. In nutrient-rich environments allowing high growth rates, we find growth-optimal metabolite concentrations


$$\begin{array}{c} \rho_{i}^{hi}≃A_{i}\sqrt{\frac{K_{i}}{\kappa_{i}}}. \end{array}$$ (1)

The proportionality constants $A_{i}$ (of order 1 h^−1/2^) depend on a pathway weight factor and the position of the enzyme within the pathway, as quantified by equation (17) in Methods. Equation (1) determines the high-growth saturation of the corresponding enzyme, $\sigma_{i}^{hi}\equiv \rho_{i}^{hi}/K_{i}$. High-growth expression levels of enzymes depend on their degree of substrate saturation,


$$\begin{array}{c} \phi_{i}^{hi}≃\frac{B_{i}}{\kappa_{i}}\left(1+\frac{K_{i}}{\rho_{i}^{hi}}\right), \end{array}$$ (2)

where the weight factor $B_{i}$ measures the contribution of the embedding pathway to biomass production (Methods). We can rewrite equation (2) as a scaling relation between enzyme and metabolite levels, $\phi_{i}^{hi}=\rho_{i}^{hi}B_{i}/A_{i}^{2}(1+\rho_{i}^{hi}/K_{i})$. Accounting for network structure and growth environment, this relation generalizes a previous result obtained from a mass minimization condition for individual metabolite-enzyme pairs [46].

In the physiological range of kinetic parameters [51], $K=10^{-4.2\pm 1.1}$ and $\kappa =10^{2.1\pm 1.3}\;{\text{h}}^{-1}$ (unit system of mass fractions specified in Methods and S1 Appendix), our model predicts broad characteristics of cell metabolism in accordance with empirical data. First, the overall mass ratio of metabolome and proteome, $\eta \equiv M_{R}/M_{P}$, is predicted to be of order η~0.1, in line with experimental values [16,59–61]. This ratio is related to the cost of metabolite dilution; i.e., the relative difference of the physiological growth rate at high nutrient supply, $\lambda^{hi}$, and the hypothetical maximum growth rate in the absence of metabolite dilution, $\lambda^{hi}=\lambda^{\text{max}}(1-2\eta /3)$ (Methods). This limit corresponds to fully saturated enzymes governed only by their catalytic rate. Second, dilution fluxes are predicted to be small in comparison to metabolite production and consumption, in agreement with observations [46,59,60]. Third, most enzymes operate near saturation at high nutrient supply ($\sigma_{i}^{hi}\gg 1$). While this result is in broad agreement with experimental data [16,46], more complex models that account for reaction reversibility and regulatory constraints can incorporate lower substrate saturation for specific enzymes.

**Fig 2:**
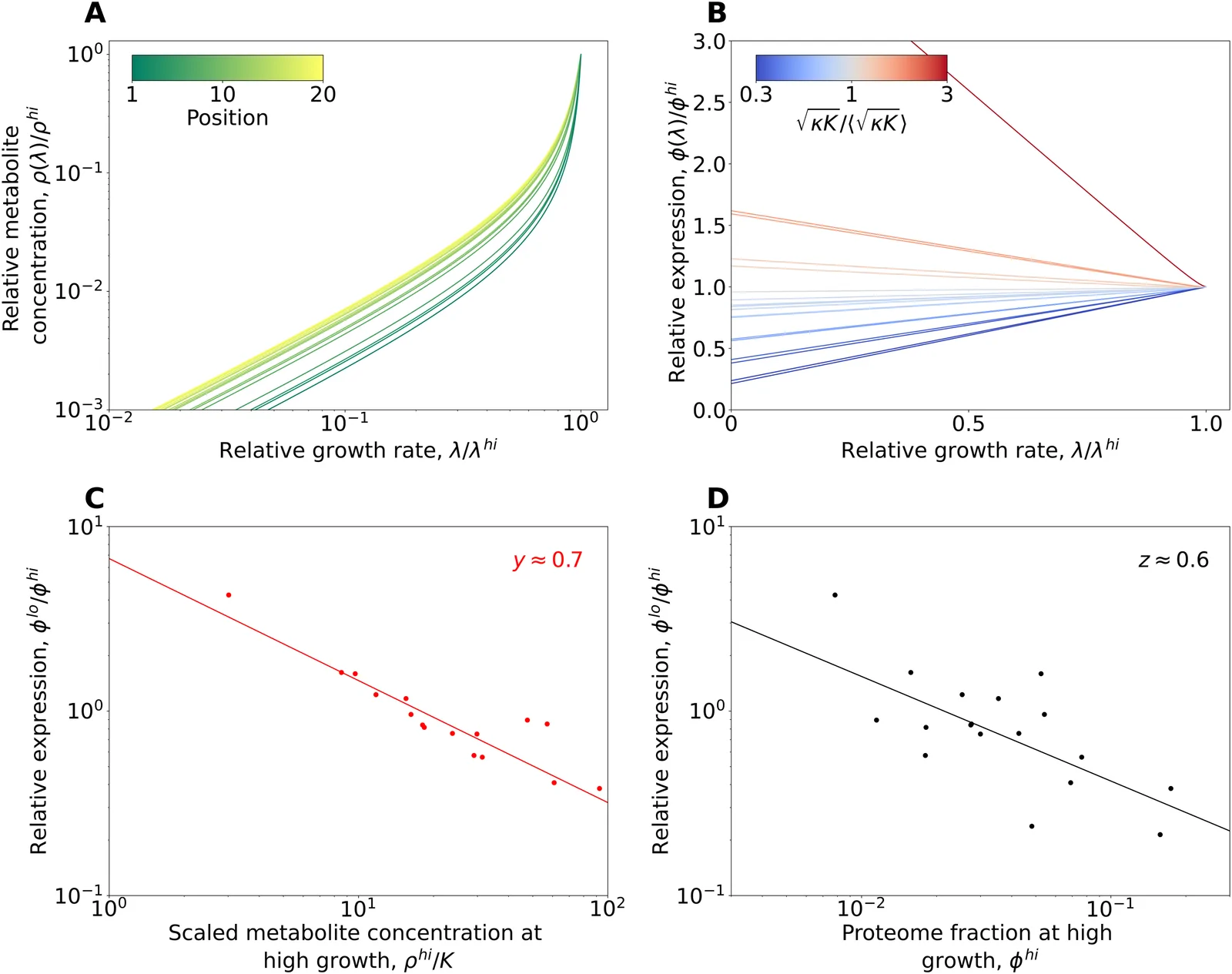
Model predictions for metabolome and proteome response to nutrient limitation. **(A)** Nonlinear growth response of metabolites in a pathway. All metabolite levels decrease with growth rate; upstream metabolites in a pathway are suppressed more strongly than downstream metabolites (green shading). The overall pattern (without the position dependence) is given by equation (5). **(B)** Heterogeneous growth response of enzymes in a pathway. Protein levels are linear functions of λ with an enzyme-specific growth response factor $q=\phi_{i}^{lo}/\phi_{i}^{hi}$ predicted by equation (3). **(C, D)** Enzyme-specific growth laws link growth response to resource allocation at high nutrient supply. **(C)** Growth response is anticorrelated with enzyme saturation at high growth, $q_{i}\sim \sigma_{i}^{-y}$ (solid line: power-law fit with y≈0.7). **(D)** Growth response is anticorrelated with expression at high growth, $q_{i}\sim (\phi_{i}^{hi}{)}^{-z}$ (solid line: power-law fit with z≈0.6). Graphs show a specific realization of a pathway of length ℓ=20 with kinetic parameters drawn from log-normal distributions $\kappa =10^{1.6\pm 0.4}\;{\text{h}}^{-1}$, $K=10^{-4.2\pm 1.1}$ with α≡Cov(logκ,logK)/Var(logκ)≈0.2 and ξ=1. The robustness of predicted correlations (y≈0.7, z≈0.6) across pathway realizations is shown in S5 Fig, the dependence on α in S4 Fig.

Under nutrient limitation, our model predicts generically linear growth responses of protein expression levels. We find response factors $Q_{i}(\lambda )\equiv \phi_{i}(\lambda )/\phi_{i}^{hi}$ of the form


$$\begin{array}{c} Q_{i}(\lambda )=\frac{\lambda }{\lambda^{hi}}+\frac{c_{i}}{\bar{c}}\;\xi_{s}\left(1-\frac{\lambda }{\lambda^{hi}}\right), \end{array}$$ (3)

as shown in Fig 2B and derived in Methods. This pattern depends on two families of network parameters. First, the kinetic factors


$$\begin{array}{c} c_{i}=\sqrt{\frac{K_{i}\kappa_{i}}{B_{i}}} \end{array}$$ (4)

capture the local reaction biochemistry weighted by pathway importance, $B_{i}$, and $\bar{c}$ denotes an appropriately weighted system average (34). The parameter $c_{i}$ is positively correlated with the catalytic rate κ, which governs the reaction flux at high substrate saturation, and negatively correlated with the specificity κ/K, which governs the flux in the unsaturated regime. Hence, it takes high values for rate-optimized enzymes (high κ and low κ/K) and low values for specificity-optimized enzymes (low κ and high κ/K). Second, the restriction factors $\xi_{s}$ characterize the local action of nutrient limitation, which curbs the metabolic flux per unit of proteome mass fraction. These factors depend only on broad *restriction sectors* of the metabolic network (labeled by an index *s*) but not on individual enzymes or pathways within the same sector. In the case of glucose uptake limitation, we use a minimal model with two restriction sectors (*s* = *R*, *U*), which partition the network into restricted enzymes (with $\xi_{R}=1$) and unrestricted enzymes (with $\xi_{U}=0$), respectively. The relation of restriction sectors to functional proteome sectors [10,14,62] is discussed below. In Methods, we give a mathematical definition of restriction factors, equations (33), (35), and we show that two broad restriction sectors emerge from the solution of a network model under nutrient limitation.

The predicted linear proteome growth response is in remarkable correspondence with experimentally observed expression changes of individual enzymes (Fig 1B and 1C). Equation (3) integrates two distinct aspects of optimal resource allocation: flux-balancing allocation tuned to an enzyme’s catalytic rate is proportional to growth rate, metabolite-minimizing allocation depends on the enzyme’s specificity and increases linearly with decreasing growth rate. We note that in a growth-optimal metabolic state, the pattern of enzyme-specific resource allocation is uniquely related to that of metabolic efficiency defined as reaction mass flux per unit of enzyme mass, $v_{i}=j_{i}/\phi_{i}$.

Specifically, the efficiency response factors $Q_{i}^{v}(\lambda )\equiv v_{i}(\lambda )/v_{i}(\lambda^{hi})$ take the form $Q_{i}^{v}(\lambda )=(\lambda /\lambda^{hi})\;Q_{i}^{-1}(\lambda )$ (Methods).

The growth response of the metabolome predicted by the model is strongly nonlinear in the growth rate but homogeneous within restriction sectors. We find response factors $Q_{i}^{\rho }(\lambda )=\rho_{i}(\lambda )/\rho_{i}^{hi}$ of the form


$$\begin{array}{c} Q_{i}^{\rho }(\lambda )≃\frac{\eta \;\lambda /\lambda^{hi}}{\eta \;\lambda /\lambda^{hi}+\xi_{s}(1-\lambda /\lambda^{hi})}; \end{array}$$ (5)

details are given in Methods, equations (20) and (29). This pattern, illustrated in Fig 2A, reflects that under nutrient limitation, the nutrient flux decreases despite up-regulation of uptake proteins. In this regime, minimizing metabolite loss by dilution becomes more important; therefore, optimal adaptation to nutrient limitation generates a global decrease of metabolite concentrations with decreasing growth rate. This minimization generates the heterogeneous response pattern of the proteome: strongly saturated enzymes retain their efficiency despite decreasing metabolite concentrations and are down-regulated in proportion to catalyzed flux. In contrast, enzymes with subaverage saturation decrease in efficiency under nutrient limitation and are up-regulated.

Together, we obtain a consistent joint pattern of nonlinear metabolome growth response and nonlinear reaction kinetics, which partly compensate each other to produce a linear, enzyme-specific growth response of the proteome. This joint response pattern is shown in S2 Fig.

The linearity of enzyme-specific growth response, although ubiquitous in the model and in the data at hand, may have limitations. In the model, linearity persists under somewhat relaxed assumptions, including altered enzyme kinetics and other optimization criteria (e.g., conversion efficiency of nutrients into protein biomass) (S1 Appendix). Empirically, linearity is also preserved for enzymes in several cyclic pathways (Fig 1), in line with model predictions over a wide range of growth rates for most enzymes in such pathways (S3 Fig). Yet, deviations from linear response are expected for reactions close to thermodynamic equilibrium, and for reactions with multiple substrates derived from the growth-limiting nutrient (S3 Fig).

The empirical evidence for growth-dependent metabolite levels is scattered at present. Available data suggest that some, but not all, metabolites are depleted by nutrient limitation [19,63,64], in line with our predictions for differential nutrient limitation of individual pathways. Similarly, regulatory responses matching increased activity of reporters of lowered amino acid concentration Lrp [65] and ArgR [66], such as increased expression of OppA, LivJ and ArtJ [14], indicate a reduction in key metabolite concentrations under carbon limitation.

### Enzyme-specific and pathway-specific growth laws for resource allocation

Our model predicts three specific correlation patterns of growth response that can be tested by matched proteome and metabolome data. A first growth law relates enzyme-specific expression growth response and saturation at high growth,


$$\begin{array}{c} q_{i}\sim \xi_{s}(\sigma_{i}^{hi}{)}^{-y}, \end{array}$$ (6)

with *y* > 0, where $q_{i}\equiv Q_{i}(0)$ denotes the response factor (3) extrapolated to zero growth, $\phi_{i}^{lo}/\phi_{i}^{hi}$. This law describes a resource shift from highly saturated to less saturated enzymes in nutrient-restricted proteome sectors ($\xi_{s}\neq 0$) (Fig 2C). To derive this law, we recall that by equations (1) and (4), saturation determines the kinetic coefficient $c_{i}=A_{i}B_{i}^{-1/2}\sigma_{i}^{-1}$, which enters the response coefficient $Q_{i}$ given by equation (3). Our full solution of individual pathways (S1 Appendix) shows that fluctuations of the position-dependent amplitude $A_{i}$ weaken this anti-correlation, generating a reduced exponent y≈0.7 (Figs 2C and S4).

A second growth law relates expression growth response and expression at high growth,


$$\begin{array}{c} q_{i}\sim \xi_{s}(\phi_{i}^{hi}{)}^{-z}, \end{array}$$ (7)

with *z* > 0. This law describes a resource shift from highly expressed to less expressed enzymes in restricted sectors ($\xi_{s}\neq 0$). For derivation, we start from the form $c_{i}\sim (K_{i}\kappa_{i}{)}^{1/2}$ and eliminate the often unknown parameter $K_{i}$ by substituting a conditional expectation value ${\bar{K}}_{i}(\kappa_{i})\sim (\kappa_{i}{)}^{\alpha }$. This expresses a well-known correlation observed in metabolic networks [51]: enzymes with larger $\kappa_{i}$ tend to have reduced target affinity; i.e., larger $K_{i}$. We obtain $q_{i}\sim c_{i}\sim \kappa_{i}^{z}$ with z=(1+α)/2. We then use equation (2) to rewrite $\kappa_{i}$ in terms of the high-growth expression level. Evaluating available data on kinetic constants in *E. coli* gives system-wide estimates α≈0.2 and z≈0.6 (S4 Fig); however, the values of these exponents may depend on individual pathways. A simple argument suggests that the underlying affinity-rate tradeoff in enzyme performance is a generic feature of evolved metabolic networks: in a fitness landscape defined by our network growth model, selection on binding affinity is weaker for enzymes with high catalytic constant $\kappa_{i}$ than for enzymes with low $\kappa_{i}$ (Methods, S4 Fig).

A third growth law operates at the level of pathways, relating the pathway mean response ${\bar{q}}_{p}$ and the rms (root mean squared) intra-pathway response variation $\Delta_{p}$,


$$\begin{array}{c} {\bar{q}}_{p}\sim \Delta_{p}, \end{array}$$ (8)

where both quantities are computed from the distribution of response factors $q_{i}$ for all enzymes in a given pathway *p* with suitable mass weights (Methods). For these enzymes, we decompose the response factors (3) in the form $q_{i}={\bar{q}}_{p}+\delta q_{i}$ with ${\bar{q}}_{p}={\bar{c}}_{p}\xi_{s}/\bar{c}$ and $\delta q_{i}=(c_{i}-{\bar{c}}_{p})\xi_{s}/\bar{c}$. In most pathways, all enzymes belong to the same restriction sector and share the same restriction factor $\xi_{s}$. Hence, our model predicts both larger ${\bar{q}}_{p}$, corresponding to a net up-regulation, and larger $\Delta_{p}$ in more restricted pathways.

### Empirical enzyme-specific growth laws in *E. coli*

**Fig 3:**
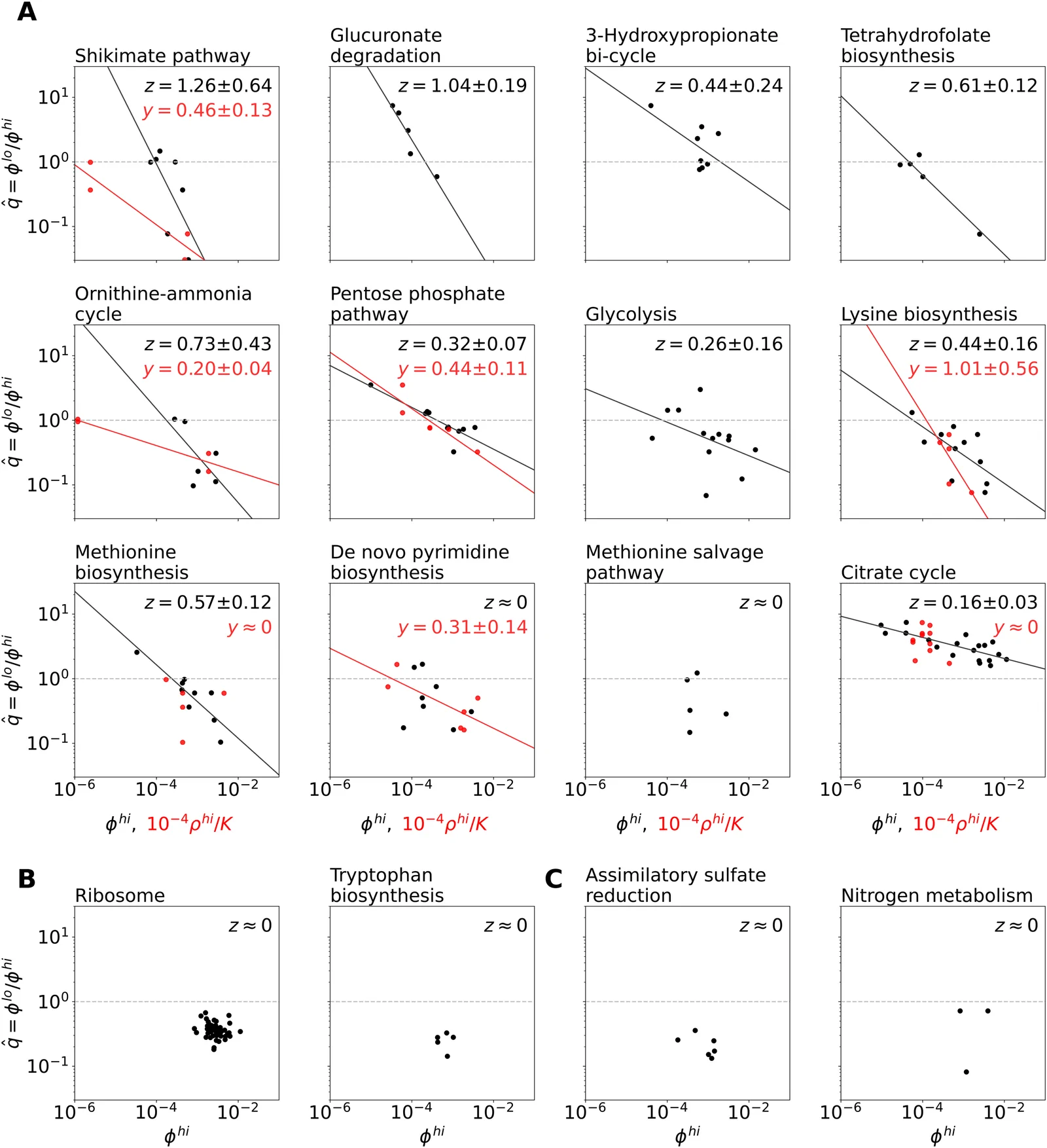
Empirical growth laws. Growth response factors, ${\hat{q}}_{i}$, of individual enzymes (from Fig 1) are plotted against the high-growth substrate saturation σ (red data points) and against the high-growth expression level, $\phi^{hi}$ (black data points). Power-law fits to the data of a given pathway, $\hat{q}\sim \sigma^{-y}$ (red lines) and $\hat{q}\sim (\phi^{hi}{)}^{-z}$ (black lines) are shown where the fit exponents indicate significant (anti-)correlations (y≠0, z≠0). The remaining cases are marked by y≈0, z≈0. **(A)** Extended pathways with heterogeneous growth response (as in Fig 1), mapped to the *R* sector. These pathways show significant (anti-)correlations of the form (7) with *z* > 0; see also Figs 2C and 2D. **(B, C)** Pathways with homogeneous growth response. **(B)** Complexes and operons; **(C)** unrestricted pathways, mapped to the *U* sector. These pathways show no significant (anti-)correlations (z≈0), consistent with predictions (see text). The full set of metabolic pathways is shown in S6 Fig and listed in S1 Table. Proteomics data from Mori et al. [14], metabolomics data from Bennett et al. [16].

To test the enzyme-specific growth laws (6) and (7), we evaluate empirical growth response parameters as follows: We define $\phi^{lo}$ by extrapolation of a linear fit to λ=0 and compute relative expression levels ${\hat{q}}_{i}\equiv \phi_{i}^{lo}/\phi_{i}^{hi}$ (the intercept of the fit lines in Figs 1B and 1C with the vertical axis), which are to be compared with the prediction of equation (3), $q_{i}=c_{i}\xi_{s}/\bar{c}$. In Fig 3, we plot these changes for the enzymes and pathways shown in Fig 1 against the saturation at high growth, σ (red) and against the expression at high growth, $\phi^{hi}$ (black). Using saturation data from ref. [16], we can test the relation (6) for 59 enzymes in 9 pathways (S1 Table). As expected, pathway-specific exponents are in the range 0 < *y* < 1 with an average $\bar{y}=0.23<0.7$, consistent with the fact that not all pathways are directly affected by nutrient limitation (this point is further discussed below). Across all pathways, the anti-correlation between protein growth response and saturation at high growth is statistically significant (*p* < 10^-4^; Fig S6 and S1 Appendix).

The second growth law, equation (7), requires only proteomics data and can be tested across all curated pathways. In 18 of 34 pathways, we infer substantial pathway-specific exponents *z* (Fig 3A, S1 Table). These exponents scatter around an average value $\bar{z}=0.4\pm 0.3$, consistent with pathway-specific nutrient limitation and differential affinity-rate trade-offs α. Across all pathways, the anti-correlation between protein growth response and expression at high growth is highly significant (*p* < 10^-15^, Fig S6). We note that empirical anti-correlations are only significant between $\hat{q}$ and $\phi^{hi}$ but not between ${\hat{q}}^{-1}$ and $\phi^{lo}$ (Fig S7). This test excludes several alternative hypotheses for their origin, such as stochastic condition-dependent expression requirements.

### Growth response of complexes and operons

The predicted growth laws (6) and (7) apply to extended pathways with enzymes regulated to growth-optimized levels independently of one another. Clear counterexamples are protein complexes forming a single enzyme with a fixed stoichiometry of subunits. Such groups are represented in the model by a single response factor *Q*; no intra-group variation of growth response is expected (y≈0, z≈0). This pattern is consistently observed in our dataset. Subunits of protein complexes in metabolic modules such as ribosome and NADH:quinone oxidoreductase (Fig 3B and S6) appear tightly co-regulated and show no anti-correlations (y≈0, z≈0; Fig 3B). Co-regulation in operons may explain the similar patterns observed for the tryptophan- and histidine-biosynthesis pathways (Fig 3B and S6). These examples serve as a negative control for the growth laws.

### Graded growth response of pathways

**Fig 4:**
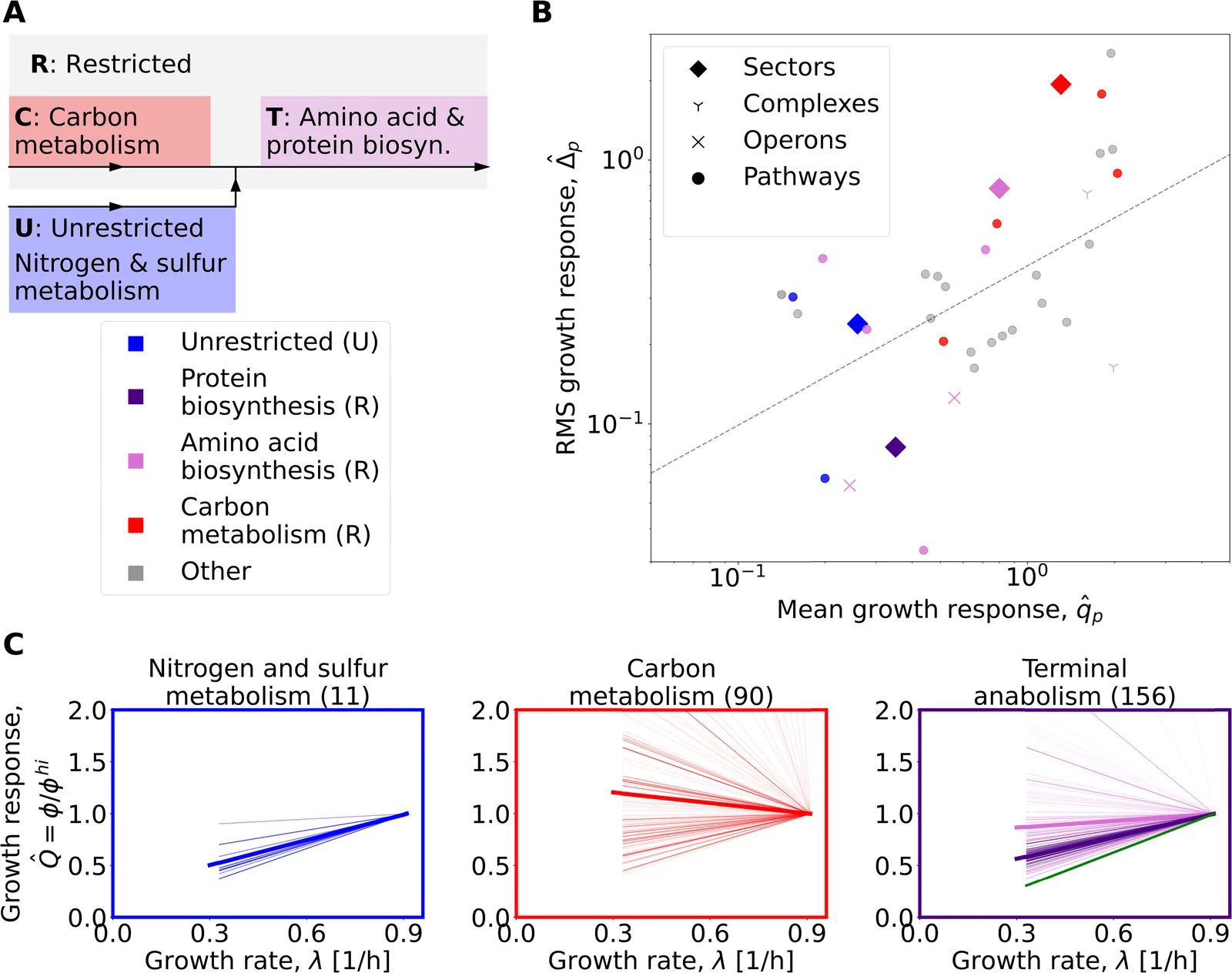
Growth response of pathways and functional sectors. **(A)** Metabolic sectors under carbon limitation. Functional sectors: *C* (carbon metabolism), *N* (nitrogen and sulfur metabolism), *T* (amino acid and protein synthesis). Restriction sectors: *R* (restricted, $\xi_{R}=1$), *U* (unrestricted, $\xi_{U}=0$). **(B)** Correlation of mean and standard deviation of growth response, ${\hat{q}}_{p}$ and ${\hat{\Delta }}_{p}$, for classes of functional units: operons, complexes, pathways, and functional sectors. To be compared with equation (8). **(C)** Growth response of the functional sectors (*C*, *N*, *T*). Linear fits of empirical response factors for individual enzymes, $Q_{i}(\lambda )$ (thin lines), and for sector averages ${\bar{Q}}_{s}(\lambda )$ (thick lines). Specific components of the *T* sector with strong depletion are the ribosome (purple) and the enzyme MetE (green). Proteomics data from Mori et al. [14]; higher line opacity indicates higher $\phi_{i}^{hi}$; sectors are defined by the respective KEGG terms eco:01200, eco:00910 and M00176, eco:01230 and eco:03010 [49].

In extended pathways, what determines the emergence of empirical growth laws? Mechanistically, broad restriction sectors arise in our model from restricted nutrient uptake flux in combination with constrained uptake enzyme expression (Methods). In the minimal network model with two restriction sectors *R* and *U*, correlations of the form (6) and (7) are predicted to occur in the restricted sector *R* (with $\xi_{R}=1$) downstream of the constrained uptake reaction but not in the unrestricted sector *U* (with $\xi_{U}=0$), which uses exclusively abundant nutrients. Remarkably, 10 of the 18 pathways with significant correlations (Fig 3A) can be mapped to known functional sectors affected by carbon limitation, including the carbon metabolism (*C*) and the terminal sector (*T*) with its sub-sectors of amino acid synthesis and protein synthesis [49] (Fig 4A). Other pathways in this group, including tetrahydrofolate biosynthesis and pyridoxal phosphate biosynthesis, are not part of central metabolism and the impact of carbon limitation is unknown; our analysis predicts these pathways to be restricted and maps them to the *R* sector (Figs 3A and S6).

Our model assigns the complementary set of extended pathways without significant correlations or known co-regulation mechanisms to the *U* sector (Fig 3C). Consistently, this set contains the nitrogen uptake sector, which is known to remain replete under carbon limitation [14] and assimilatory sulfate reduction, a pathway processing carbon-free metabolites. Other pathways in this group are degradation-associated pathways such as methionine salvage, which are likely unaffected by carbon limitation (Figs 3 and S6).

Within one restriction sector, the restriction factor $\xi_{s}$ is constant, and our model associates growth response differences with variation of the local kinetic parameter *c*. This is again in accordance with available data for specific pathways. For example, enzymes of the catabolic citrate cycle appear biased toward high catalytic constants [51], consistent with the observed growth response (${\hat{q}}_{\text{Cit}}=2.46$, Fig 3A and Fig 4). In contrast, the ribosome has a small aggregate catalytic rate per unit mass [1], $\kappa_{\text{Rib}}/\bar{\kappa }\approx 10^{-1}$, and a small binding constant [67,68], $K_{\text{Rib}}/\bar{K}<10^{-1}$, generating a sub-average kinetic parameter, $c_{\text{Rib}}/\bar{c}\sim 0.1$. Hence, equation (3) predicts strong down-regulation under growth limitation, in agreement with experimental data (${\hat{q}}_{\text{Rib}}=0.37$, Fig 3B). Similarly, methionine synthase (MetE) has high molecular mass and, hence, a small catalytic constant κ per unit of mass. This enzyme is among those with the strongest depletion observed in our dataset (${\hat{q}}_{\text{MetE}}\approx 0$, Fig 4C).

The pathway growth law (8) describes the resulting variation of growth response across the entire set of pathways. To test this prediction of our model, we determined the empirical mass-weighted mean response and rms variation, ${\hat{q}}_{p}$ and ${\hat{\Delta }}_{p}$, for all curated pathways in our dataset. In broad agreement with the model prediction, the empirical data show a strong correlation of mean and intra-pathway variation of growth response for this set, ${\hat{\Delta }}_{p}\sim ({\hat{q}}_{p}{)}^{0.67\pm 0.18}$ (*p* = 0.00076) (Fig 4B).

Together, we conclude that two key quantities determine the growth response of individual enzymes and metabolic pathways: the broadly varying restriction factor $\xi_{s}$ and the local kinetic parameter $c_{i}$.

### Growth response of proteome sectors

Our model predicts three key features of growth-dependent resource allocation to broad functional sectors, here the uptake sectors *C* and *N* and the terminal sector *T*. Here we compare these features with empirical patterns of sector mean and rms response, ${\hat{q}}_{s}$ and ${\hat{\Delta }}_{s}$ (*s* = *C*, *N*, *T*), in our dataset (Figs 4B and 4C).

First, the variance of growth response is graded by restriction sector. As predicted by the model, the restricted sectors *C* and *T* show substantially higher overall variation of response (${\hat{\Delta }}_{C}=1.92$ and ${\hat{\Delta }}_{T}=0.59$) than the unrestricted sector *N* (${\hat{\Delta }}_{N}=0.30$) (Fig 4B). This grading is consistent with the variance pattern of individual pathways.

Second, the carbon metabolism *C* is up-regulated under carbon limitation (${\hat{q}}_{C}=1.31$), while nitrogen metabolism *N* is down-regulated (${\hat{q}}_{N}=0.29$) (Fig 4B). Our model explains this resource shift by a difference in restriction factors (C⊂R with $\xi_{R}=1$, N⊂U with $\xi_{U}=0$), in line with the rationale of previous sector models [10,14,62].

Third, the terminal sector *T* is down-regulated under carbon limitation (${\hat{q}}_{T}=0.57$), as well as under other types of growth limitation [10,14]. The growth-dependent resource shift from *T* to *C* cannot be explained by graded restriction, because both sectors are strongly affected by nutrient limitation (T,C⊂R). Instead, our model explains this pattern by a large-scale bias in enzyme kinetics (${\bar{c}}_{T}/\bar{c}\approx 0.53$). This bias is largely generated by two key components that are heavy and slow: the ribosome and the enzyme MetE, which is involved in amino acid biosynthesis, specifically the final step of methionine biosynthesis ($\phi_{Rib}^{hi}/\phi_{T}^{hi}=0.52$, $\phi_{MetE}^{hi}/\phi_{T}^{hi}=0.2$ and ${\bar{c}}_{Rib}/\bar{c}\approx 0.1$, ${\bar{c}}_{MetE}/\bar{c}\approx 0$, see above). Consistently, the observed down-regulation is particularly strong for these two components (${\hat{q}}_{Rib}=0.37$, ${\hat{q}}_{MetE}\approx 0$).

### Metabolite-mediated regulation can approximate optimal growth response

**Fig 5:**
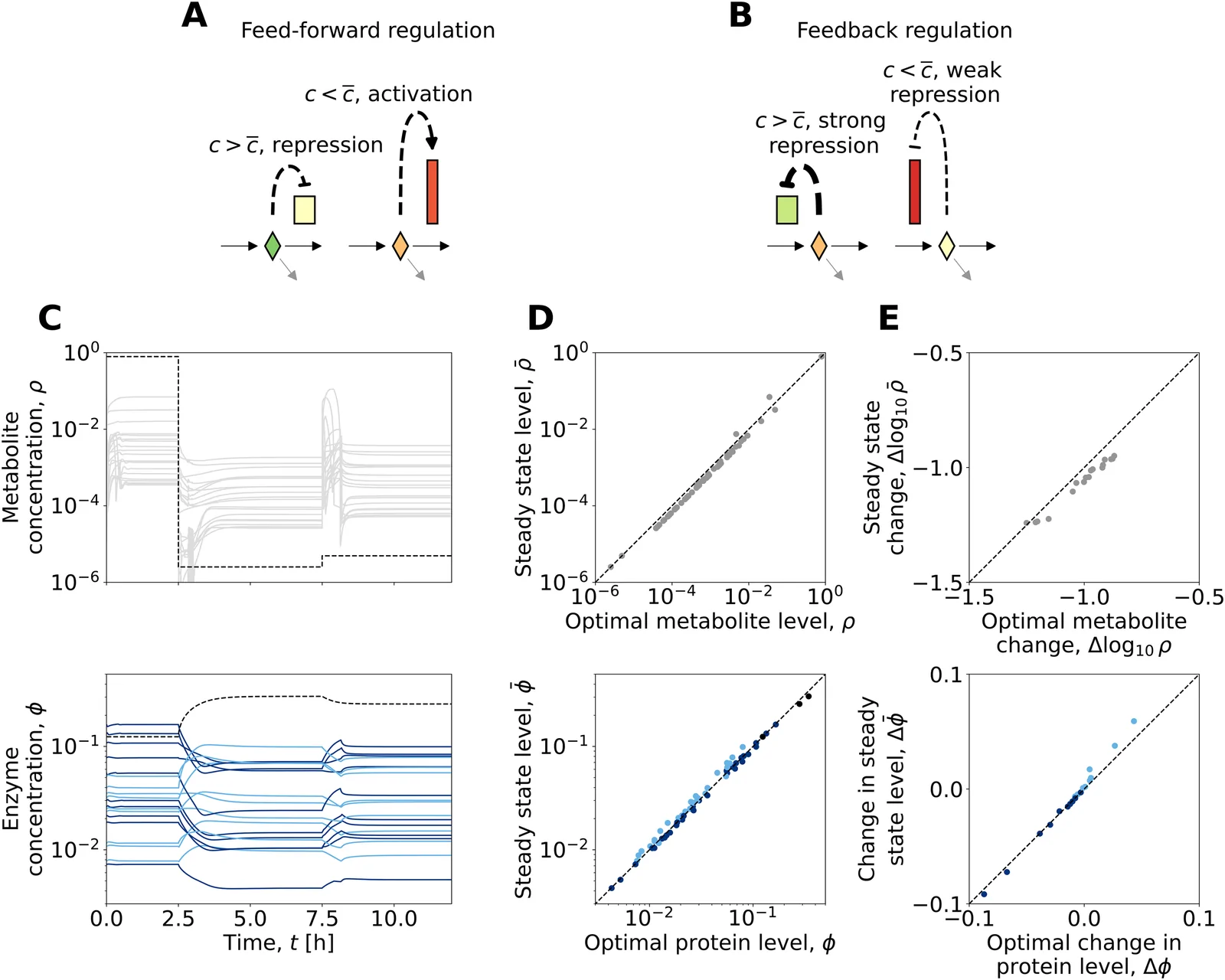
Metabolite-mediated regulation. **(A, B)** Local regulatory mechanisms. **(A)** Feed-forward regulation. Metabolites regulate the transcription of their cognate enzymes, inducing repression for rate-optimized enzymes ($c_{i}/\bar{c}>1$) and activation for specificity-optimized enzymes ($c_{i}/\bar{c}<1$). **(B)** Feedback regulation. Metabolites regulate the transcription of their producing enzymes, inducing differential repression for rate-optimized and specificity-optimized enzymes. **(C)** Time-dependent metabolite levels for feed-forward regulation in a linear pathway, $\rho_{i}(t)$ (gray), and regulated protein concentrations $\phi_{i}(t)$ (dark blue: specificity-optimized enzymes, $c/\bar{c}<1$; light blue: rate-optimized enzymes, $c/\bar{c}>1$, dashed: uptake enzyme) under time-dependent nutrient limitation, $\rho_{0}(t)$ (top, dashed). **(D)** Regulated late-time stationary levels for feed-forward regulation, ${\bar{\rho }}_{i}$ and ${\bar{\phi }}_{i}$, plotted against growth-optimal levels. **(E)** Comparison of the regulated response between steady states at *t* = 2 h and at *t* = 7 h with the respective growth-optimal response. See S8 Fig for time-dependent and stationary levels under feedback regulation.

Previous work has shown that metabolite-based regulation can induce metabolite and enzyme concentrations that converge from a perturbed state to a near-optimal stationary state at a given growth rate [69]. Here we show that the optimal state of our model – i.e., growth response factors $Q_{i}(\lambda )$ and $Q_{i}^{\rho }(\lambda )$ given by equations (3) and (5) – can be reached by simple, local regulation of enzymes mediated by metabolites (Fig 5A) with analytically accessible regulatory parameters and under concurrent optimization of growth rate. In a minimal model of transcriptional regulation, the stationary level ${\bar{\phi }}_{i}=\phi_{i}^{0}+a_{i}/(1+K_{i}^{\text{reg}}/\rho )$ depends on the basal level $\phi_{i}^{0}$ and the equilibrium binding of a transcription factor present at density ρ, which is parameterized by the amplitude $a_{i}$ and the binding constant $K_{i}^{\text{reg}}$. We consider two kinds of regulatory mechanisms: feed-forward regulation, where the substrate density sets the transcription signal ($\rho \sim \rho_{i}$), and feedback regulation, where the reaction product takes this role ($\rho \sim \rho_{i+1}$). For both mechanisms, we find that already the minimal model can realize near-optimal stationary levels, given parameters $\phi_{i}^{0},a_{i},K_{i}^{\text{reg}}$ that are simple functions of the local kinetic constants $K_{i}$ and $\kappa_{i}$ (Methods, S1 Appendix). For feed-forward regulation, parameters follow directly from the functions linking growth-optimal metabolite and enzyme concentrations (grid lines in S2 Fig); that is, rate-optimized enzymes ($c_{i}>\bar{c}$) are repressed, specificity-optimized enzymes ($c_{i}<\bar{c}$) are activated by their cognate metabolite, in accordance with equation (3). The regulated steady-state resource allocation sets a growth rate within ~2%, enzyme concentrations within ~5%, and metabolite concentrations within ~50% relative to the growth-optimal state at all levels of nutrient supply (Figs 5D and S8B). Perturbed metabolic states quickly settle to the stationary levels (Figs 5C and S8A). *In vivo*, more complicated regulatory mechanisms are at play [70], and enzymes may not all be regulated by their own substrates or products. Instead, broad regulatory circuits with target-specific effects [65] may form the mechanistic basis of enzyme-specific growth responses. Our result suggests that multiple architectures of metabolite-mediated regulation, including feed-forward activation and feedback inhibition, can closely approximate growth-optimal resource allocation.

## Discussion

Microbes regulate their system-wide metabolism in response to nutrient limitation. Growth-dependent proteomics data for *E. coli* [10,14] reveal a striking pattern: despite pervasive nonlinearities in the underlying mechanisms, specifically regulation and reaction kinetics, the expression of most enzymes is a linear function of the growth rate. The direction and amplitude of these changes are strongly heterogeneous, generating growth-dependent resource shifts even within a single pathway. Importantly, such heterogeneous proteome response is inextricably tied to growth-dependent metabolite levels. In a typical pathway with homogeneous changes of metabolic flux, reaction kinetics must change heterogeneously to match heterogeneous expression changes.

The metabolic model developed in this paper proposes a simple ordering scheme of proteome resource reallocation: the system-wide pattern follows a two-dimensional gradation, by restriction factor and by kinetic parameter. Restriction factors measure the differential pressure of growth limitation and are broadly distributed across the network, taking homogeneous values $\xi_{s}$ within large restriction sectors of the proteome. Metabolite levels mediate restriction pressures; they decrease with growth rate and respond particularly strongly in restriction sectors with large $\xi_{s}$. Kinetic parameters $c_{i}\sim (\kappa_{i}K_{i}{)}^{1/2}$ quantify the susceptibility of specific reactions to restriction pressure, capturing local biochemical biases of the metabolic network; the susceptibility is higher for rate-optimized enzymes than for specificity-optimized enzymes. The pattern of enzyme-specific growth response emerging from our model, $q_{i}\sim c_{i}\xi_{s}$, integrates the variation of restriction pressure and of susceptibility: (up-)regulation increases with restriction pressure and with local susceptibility to pressure.

The integration of broadly varying restriction pressure and local susceptibility to pressure distinguishes our approach from previous models of metabolic growth response [27,43,47,71]. These models compute broad distributions of expression changes depending on growth-dependent efficiencies – metabolic fluxes per unit of enzyme mass, $v_{i}$ – used as a measure of differential restriction. However, they do not map the local variation in enzyme kinetics to differential changes of $v_{i}$ under nutrient limitation. Our model shows that in a biochemically structured network, the growth response of efficiencies is enzyme-specific and double-graded: $v_{i}$ depends on the restriction factor and on the local biochemical bias. Hence, the system-wide pattern of efficiencies is as heterogeneous as that of enzyme-specific growth response. In other studies, the net growth response of functional sectors has been derived from differential flux restriction [10,14,21,72], again without accounting for local variation in enzyme kinetics. These studies have used empirical enzyme-specific growth response factors to partition enzymes into functional sectors. Our finding of a double-graded response pattern with strong heterogeneity within pathways and functional groups alters this inference.

The data analysis presented in this paper shows evidence for our model across different scales of the metabolic network. For *E. coli* under carbon limitation, we find a ubiquitous linear growth response for individual enzymes, metabolic pathways, and large functional sectors (Fig 1). The amplitudes of change are clearly graded by restriction sectors, which closely align with known functional sectors [49], and show multiple signatures of local biochemical biases. Three growth laws, which describe statistical correlations derived from the predicted double-graded response pattern, can be validated in this dataset (Figs 3 and 4). Notably, the double gradation includes the large-scale resource allocation (Fig 4). Different uptake sectors, here carbon and nitrogen metabolism, respond differently because of differential restriction. In contrast, the resource shift from terminal metabolism to carbon uptake, which are both strongly restricted, follows from a biochemical bias generating lower susceptibility to growth limitation in the terminal sector. More broadly, our model aligns with systems-level observations of regulatory heterogeneity – the same Gene Ontology terms often encompass proteins with divergent regulatory behaviors [14] – and of biochemical biases, suggesting that kinetic parameters are broadly correlated with proteome sectors [51,73].

Other predictions of our model remain to be tested in future work. Most importantly, the model predicts a strong and homogeneous decrease of metabolite levels with decreasing growth rate. This prediction addresses the joint organization of proteome and metabolome under diverse growth conditions – a longstanding open question [61]. Currently, our predictions are consistent with available metabolomics data at high growth (Fig 3) and with limited evidence for growth-dependent metabolite levels under nutrient limitation [19,63–65]. A more comprehensive test of the model requires system-wide growth-dependent metabolomics data, which are not available at present. Other open questions are the extension of the model to complex nonlinear pathways, growth-dependent switches in pathway usage [74] (e.g., between fermentation and respiration), and reaction kinetics beyond the Michaelis-Menten approximation, especially for reactions close to thermodynamic equilibrium [75]. Finally, the model remains to be tested for other nutrient limitations and for other microbes, e.g., natively slow-growing bacteria with a metabolic network architecture that may follow different optimization criteria [5].

Our key result of growth-dependent, near-optimal resource allocation at the level of individual enzymes raises an immediate question: can such optimization be realized by realistic regulatory mechanisms? Here we have shown that growth-optimality is accessible by simple, metabolite-based regulation mechanisms (Fig 5). We hypothesize that microbes have evolved such regulatory mechanisms to optimize their growth even when nutrient uptake is constrained, likely because nutrient limitation is common in microbial growth environments. *In vivo*, enzyme concentrations are presumably not all optimized individually. Instead, few master regulators may suffice to signal the nutrient supply state [70], and co-regulation in operons may approximate the joint growth-optimal response of several enzymes. Our model suggests that enzymes can efficiently be co-regulated in an operon if they are affected by the same nutrient limitations and share similar growth-optimal responses to nutrient limitation by their kinetic constants.

Computable gene-specific growth response patterns may also enable new interventions into microbial growth that are targeted to a particular growth environment. Empirical principles describing resource reallocation, combined with flux-balance analysis [8] and coarse-grained models [5,21], have already enabled predictions of dose responses to ribosome-targeting antimicrobials [76] and resistance evolution under antibiotic pressure [77]. However, generic response patterns for enzymes beyond the ribosome remain elusive. As a result, predicting dose responses for antibiotics targeting more deeply embedded metabolic enzymes has remained challenging [78,79]. Enzyme-specific growth laws fill this gap. More broadly, elucidating the interplay among growth environment, physiology, and evolution is essential for the rational design of antimicrobial strategies that minimize resistance evolution – an important direction for future research.

## Methods

### Networks of metabolites and enzymes

We consider a metabolic pathway comprising ℓ enzymes. Each enzyme catalyzes a reaction that follows Michaelis-Menten kinetics; as estimated in S1 Appendix, metabolic reactions in *E. coli* are, on average, driven far from equilibrium, thereby facilitating rapid growth. Out of equilibrium, reaction rates depend predominantly on substrate and enzyme concentrations.

In a cell of volume *V*, an enzyme present at mass $M_{i}$ acts on a metabolite present at mass concentration $\rho_{i}$ with catalytic constant $\kappa_{i}$ (scaled here per unit of enzyme mass) and substrate-binding affinity $K_{i}^{-1}$, generating the mass flux


$$\begin{array}{c} J_{i}=\frac{\kappa_{i}M_{i}}{1+\frac{K_{i}}{\rho_{i}}}. \end{array}$$ (9)

At finite substrate concentrations, the metabolite mass flux is the product of enzyme mass, catalytic constant per unit mass, and the enzyme’s fractional saturation with its substrate. To streamline notation, we assign a single index *i* to each reaction step, so that $M_{i}$ denotes the enzyme, $\rho_{i}$ its substrate, and $J_{i}$ the resulting flux. This bookkeeping is natural for linear chains, which we analyze first before assembling them into more complex, branched, or cyclic networks. Here we consider pathways that are required for the conversion of environmental nutrients, present at concentrations $\rho_{i}$, into protein biomass.

The efficiency of the metabolic process, defined as reaction mass flux per unit of enzyme mass, can be evaluated at different levels of the network. For individual Michaelis-Menten reactions, the efficiency $v_{i}\equiv J_{i}/M_{i}$ follows directly from equation (9),


$$\begin{array}{c} v_{i}=\frac{\kappa_{i}}{1+\frac{K_{i}}{\rho_{i}}}. \end{array}$$ (10)

For pathways, the efficiency is given in a similar way by


$$\begin{array}{c} v_{p}=\frac{J_{p}}{M_{p}}, \end{array}$$ (11)

where $M_{p}$ is the total enzyme mass of the pathway and $J_{p}$ denotes the pathway’s output flux that contributes to biomass production. Pathways contribute to biomass production with weight factors $B_{p}$; constraint-based models, such as flux-balance analysis, determine these weights in complex metabolic networks [8]. At the systems level, the total rate of biomass production (i.e., protein biosynthesis), $J_{P}$, is to be compared to the total proteome mass $M_{P}$. Therefore, the net metabolic efficiency of the cell equals the growth rate,


$$\begin{array}{c} \lambda =\frac{J_{P}}{M_{P}}. \end{array}$$ (12)

In line with empirical data from microbial systems, we neglect protein degradation relative to protein dilution [15,80] and assume that the total intracellular protein concentration remains constant at all times [1,13,52–54]; hence, cell volume is proportional to total protein biomass $M_{P}$. Accordingly, we write concentrations as proteome mass fractions, $\rho_{i}=R_{i}/M_{P}$ and $\phi_{i}=M_{i}/M_{P}$; we use mass-normalized fluxes, $j_{i}=J_{i}/M_{P}$, and dimensionless affinities $K_{i}^{-1}$ (i=1,...,ℓ) (normalized by the intracellular protein mass concentration). Details are given in S1 Appendix. A list of variables is provided as S2 Table.

Mass conservation for pathway metabolites takes the form


$$\begin{array}{c} \frac{d\rho_{i}}{dt}=\frac{J_{i-1}-J_{i}}{M_{P}}-\lambda \rho_{i}\;(i=1,...,\ell ). \end{array}$$ (13)

### Optimal balanced growth states

We consider balanced exponential-growth states [58] ($\rho_{i}=\text{const.}$ for i=1,...,ℓ) that maximize growth rate under given nutrient conditions, i.e., a defined supply flux *j*_0_, $S^{⋆}=(\rho^{⋆},\phi^{⋆})=\text{arg}\max(\lambda (𝒮)){|}_{j_{0}}$, averaging over stochastic and deterministic fluctuations. Importantly, balanced growth states sustain a flux mismatch $\lambda \rho_{i}$ between metabolite influx and outflux at each step, reflecting dilution of metabolites by growth [29,40,44,45,55–57]. While experimentally observed protein expression levels often closely match their growth-optimal steady-state values [43,81], this assumption is not a priori obvious [5] and is tested by comparison of model results with empirical data.

By equation (12), maximal growth is achieved if the protein mass necessary to produce a given protein production flux $J_{P}$ is minimal [48]. We use the Michaelis-Menten relation (9) and the continuity equation (13) in steady state for each reaction to express the enzyme masses $M_{k}$ in terms of the current vector $J=(J_{1},\;...,\;J_{\ell })$, and the set of kinetic constants (κ,K). Additionally, we account for the mass cost $M_{r}(J_{0})$ of sustaining the supply flux $J_{0}=M_{P}j_{0}$. Thus, we can formulate the maximum-growth condition in differential balance equations [44],


$$\begin{array}{c} \frac{\partial M_{r}(J_{0})+\sum_{k=1}^{\ell }M_{k}(J,\kappa ,K)}{\partial J_{i}}{|}_{J^{⋆},j_{0}}=0\;(i=1,\;...,\;\ell -1). \end{array}$$ (14)

Equation (14) corresponds to Enzyme Cost Minimization [48] – the minimization of the enzyme mass required for a given pathway output – in a pathway with metabolite dilution. Thus, our model assumptions on enzyme kinetics and metabolite dilution match those of Growth Balance Analysis [44] applied to a metabolic pathway. Importantly, we here assume no constraint on the joint mass of proteome and metabolome. Instead, we find that such a constraint arises from the optimization of growth rate, when we rewrite equation (14) for intensive variables,


$$\begin{array}{c} \rho_{i}^{⋆}=\underset{\rho_{i}}{\text{arg}\min}(\beta_{i}^{⋆}\rho_{i}+\phi_{i}(\rho_{i},j_{i}^{⋆},\kappa_{i},K_{i})){|}_{j_{i}^{⋆},\beta_{i}^{⋆}} \end{array}$$ (15)

with


$$\begin{array}{c} \beta_{i}^{⋆}\equiv \beta_{0}^{⋆}(j_{0})+\sum_{k=1}^{i-1}\frac{\lambda^{⋆}}{v_{k}}. \end{array}$$ (16)

The *metabolite cost* parameter $\beta^{⋆}$ accounts for the upstream proteome resources required to sustain an increased focal metabolite concentration against metabolite dilution, and constitutes a key difference of our model in comparison to previous models of resource allocation to individual enzymes and their substrates [46]. In particular, $\beta_{0}$ corresponds to proteome resources for increasing the supply flux *j*_0_, whereas the sum corresponds to additional proteome resources in the pathway of interest. At high nutrient supply, this cost is typically of order one (S1 Appendix); to optimize growth, resource allocation must minimize metabolite mass fractions with a similar stringency as proteome mass fractions.

### Metabolite-enzyme relations

Although the full analytical solution (S1 Appendix) depends on all kinetic parameters and the global network context, robust local properties of growth-optimal resource allocation nonetheless emerge. Combining the optimality condition (15) with the kinetic rate law (9), we obtain remarkably simple constitutive relations for cellular resource allocation,


$$\begin{array}{ccc} \rho_{i}^{⋆} & = & \sqrt{\frac{K_{i}}{\kappa_{i}}}\sqrt{\frac{j_{i}^{⋆}}{\beta_{i}^{⋆}}}, \end{array}$$ (17)

$$\begin{array}{ccc} \phi_{i}^{⋆} & = & \beta_{i}^{⋆}\rho_{i}^{⋆}\left(1+\frac{\rho_{i}^{⋆}}{K_{i}}\right)\;(i=1,\;...,\;\ell ). \end{array}$$ (18)

The first relation determines $\rho_{i}$ as a function of the local kinetic parameters $K_{i}$, $\kappa_{i}$ and the network-dependent variables $j_{i}$, $\beta_{i}$. The second relation links enzyme and metabolite mass fractions. Equation (18) generalizes a scaling relation derived by Dourado et al. [46] from a mass minimization condition for an individual metabolite-enzyme pair; the constitutive relations (17, 18) include an additional cost parameter $\beta_{i}$, which encodes the influence of both nutrient environment and network structure on optimal resource allocation as given by equation (15). In the main text, the influence of variables other than the kinetic constants is summarized by the pathway- and position-dependent amplitude factors, $A_{i}\equiv \sqrt{j_{i}^{⋆}/\beta_{i}^{⋆}}$ and $B_{i}\equiv j_{i}^{⋆}$ evaluated at high growth. Specifically, the amplitude factors depend on the fraction of biochemical flux carried by the pathway, γ, via $j_{i}^{⋆}\approx \gamma \lambda^{⋆}$, and on an enzyme’s position in the pathway via costs $\beta^{⋆}$.

The resulting resource allocation patterns are organized by two key parameters: the metabolite cost $\beta_{i}$ and the growth response parameter $c_{i}$ (grid lines in S2 Fig). Here, $c_{i}\sim \sqrt{K_{i}\kappa_{i}}$ summarizes the effect of a reaction’s kinetic parameters on optimal resource allocation. In a given environment, optimal enzyme concentrations and optimal metabolite concentrations are correlated. Reactions with low substrate saturation $\rho_{i}/K_{i}$ also exhibit low scaled expression levels $\phi_{i}/K_{i}$ [46]. Variation in the growth response parameter $c_{i}$ induces a crossover from a linear enzyme-metabolite relation at low saturation ($\phi_{i}\sim \rho_{i}$ for $\rho_{i}/K_{i}≲1$) to a quadratic relation at high saturation ($\phi_{i}\sim {\rho_{i}}^{2}$ for $\rho_{i}/K_{i}≳1$).

We derive in S1 Appendix that costs in nutrient-rich environments are of order 1. To identify physiological parameter regimes, we determine the geometric mean kinetic constants for enzymes of *E. coli* in our mass-fraction-based unit system. Specifically, we normalize previously reported kinetic constants [51] by the respective enzyme and metabolite mass (S1 Appendix) and we determine the correlation between matched values of logK and logκ as


$$\begin{array}{c} \alpha \equiv \frac{Cov(\log K,\log\kappa )}{Var(\log\kappa )}. \end{array}$$ (19)

This yields the distributions $K=10^{-4.2\pm 1.1}$ and $\kappa =10^{2.1\pm 1.3}\;{\text{h}}^{-1}$ and the correlation α≈0.2. Together, they set the corresponding affinity-scaled metabolite and protein levels predominantly in the high-saturation regime, $\rho_{i}^{hi}/K_{i},\phi_{i}^{hi}/K_{i}≳1$ (S2 Fig), consistent with empirical data [16] and previous analytical modeling [46]. Therefore, optimal proteome fractions at high nutrient supply are predominantly set by catalytic constants, $\phi_{i}^{hi}\sim 1/\kappa_{i}$.

### Systems-level consequences of metabolite dilution

We apply the pathway model derived above to investigate the systems-level consequences of metabolite dilution on optimal resource allocation at high nutrient supply and under nutrient limitation. If the pathway represents the complete metabolic network, we can solve equations (14) analytically for the full network under variable nutrient input (S1 Appendix). We find that the effects of metabolite dilution can be included by a power-series expansion around the strict flux-balance limit in the parameter


$$\begin{array}{c} \epsilon =\sum_{k}{\left(\frac{K_{k}B_{p(k)}}{\kappa_{k}}\right)}^{1/2}, \end{array}$$ (20)

which we term *dilution strength*. The weight factors $B_{p}$ measure the contributions of pathways to biomass production, as introduced in the main text. The distribution of kinetic parameters (S4 Fig) [51] sets the dilution strength ϵ≈0.05, and determines how strongly growth-optimal resource allocation deviates from standard flux-balance solutions. In the limit of vanishing metabolite dilution, here ϵ→0, our model reproduces the result of standard flux-balance analysis at high nutrient supply: fluxes are conserved throughout the network ($j_{i}=\lambda^{\text{max}}$), all enzymes run at full saturation with rates $\kappa_{i}$ and optimal expression levels proportional to $\left(1/\kappa_{i}\right)$, and metabolites are neglected ($\rho_{i}=0$). For small ϵ, we find a self-consistent solution


$$\begin{array}{ccc} \rho_{i}^{hi} & ≃ & \epsilon \frac{s_{i}}{\sqrt{\beta_{i}}}, \end{array}$$ (21)

$$\begin{array}{ccc} \phi_{i}^{hi} & ≃ & \frac{B_{p(i)}}{\kappa_{i}}(1+2\epsilon (1-\sqrt{\beta_{i}}))+\epsilon s_{i}\sqrt{\beta_{i}}, \end{array}$$ (22)

$$\begin{array}{ccc} \lambda^{hi} & ≃ & \left(1-\frac{4}{3}\epsilon \right)\;\lambda^{\max} \end{array}$$ (23)

with $s_{i}=\sqrt{K_{i}B_{p(i)}/\kappa_{i}}/(\sum_{k}\sqrt{K_{k}B_{p(k)}/\kappa_{k}})$, $\lambda^{\text{max}}=(\sum_{k}(1/\kappa_{k}){)}^{-1}$, and $B_{p}=\lambda^{hi}$ in this case (S9 Fig). The factor $1+2\epsilon (1-\sqrt{\beta_{i}})$ in (22) accounts for intra-pathway variation in flux.

Using a mean-field approximation for metabolite costs, we estimate the fraction of nutrient uptake lost by dilution, $\sum_{i}(\lambda \rho_{i})/j_{0}=M_{R}/(M_{R}+M_{P})$, which is directly linked to the metabolome-proteome mass ratio at high nutrient supply,


$$\begin{array}{c} \frac{M_{R}}{M_{P}}≃2\epsilon , \end{array}$$ (24)

with corrections of order $O(\epsilon^{2},\;\epsilon /\ell )$ (S1 Appendix). The predicted value, $\eta \equiv M_{R}/M_{P}\sim 10^{-1}$, agrees with empirical estimates [16,59–61], supporting our assumption that metabolite dilution is a dominant determinant of growth-optimal metabolite levels.

### Growth response of restricted pathways

Our model of system-wide growth response to nutrient limitation involves two components: the resource allocation within pathways restricted by the nutrient and the allocation between pathways with differential degree of restriction. In a first step, consider a pathway downstream of the uptake enzyme processing a limited nutrient. Cells respond to a reduced density of external nutrients by up-regulating the cognate uptake machinery. We model this response as a nutrient-dependent expression of a regulated uptake enzyme, given by $\phi_{\nu }(\rho_{\nu })=\phi_{\nu }^{0}+(\phi_{\nu }^{\text{max}}-\phi_{\nu }^{0})/(1+\rho_{\nu }/K^{\text{reg}})$, which varies between a basal level $\phi_{\nu }^{0}$ at high nutrient concentrations ($\rho_{\nu }\gg K^{\text{reg}}$) and a maximal level $\phi_{\nu }^{\text{max}}$ at low concentrations. The upper bound $\phi_{\nu }^{\text{max}}$ may arise from various biological constraints, including spatial limitations on the cell membrane, diffusive limits on nutrient uptake at low supply [82], and a maximum relative expression change achievable by regulatory mechanisms (S1 Appendix). Limited over-expression of the uptake machinery has been observed under glucose limitation in chemostat experiments [11]. Such regulated uptake mechanisms constrain the metabolic flux of the uptake step compared to the high-growth state, as measured by a flux reduction factor


$$\begin{array}{c} Q^{j}=\frac{j_{0}}{j_{0}^{hi}}. \end{array}$$ (25)

Hence, nutrient limitation generates a broadly constrained optimal metabolic state, ${𝒮}^{⋆}(\rho_{\nu },\phi_{\nu }^{\text{max}})$. Because *j*_0_ is bounded, the pathway output $j_{p}=j_{0}-\lambda \sum_{k\in p}\rho_{k}$ can increase only through a reduction of metabolite dilution. Hence, in equation (17), the limitation-induced reduction of the fluxes $j_{i}^{⋆}$ goes along with an increase of the metabolite costs $\beta_{i}^{⋆}$. Here we parametrize these changes by a single excess cost parameter $\beta_{0}$, as given by equation (16). In S1 Appendix, we identify $\beta_{0}$ with a Lagrange multiplier applicable to all reactions in the pathway. The growth-optimal value $\beta_{0}^{⋆}(Q^{j})$ can be computed by a self-consistent closure of the constitutive relations (17) and (18), using the constraint $\phi_{p}=\sum_{i\in p}\phi_{i}$. Given $\beta_{0}^{⋆}\gg \beta_{i}^{hi}$ over a wide range of flux reductions ($1-j_{p}/j_{p}^{hi}≳\epsilon$), we can self-consistently approximate $\beta_{i}^{⋆}≃\beta_{0}$ and $j_{i}≃Q^{j}j_{p}^{hi}$. We obtain a uniform excess metabolite cost


$$\begin{array}{ccc} \beta_{0}(Q^{j}) & ≃ & \frac{(\phi_{p}(Q^{j})-\phi_{\nu }(Q^{j})-Q^{j}\sum_{k}B_{k}/\kappa_{k}{)}^{2}}{(\sum_{k}\sqrt{B_{k}Q^{j}K_{k}/\kappa_{k}}\;{)}^{2}} \end{array}$$ (26)

$$\begin{array}{ccc} & \approx & \frac{(Q_{p}-Q^{j}{)}^{2}}{Q^{j}{\bar{c}}_{p}^{2}}, \end{array}$$ (27)

which describes the effect of nutrient limitation on metabolite levels in the constrained optimal state ${𝒮}^{⋆}(\rho_{\nu },\phi_{\nu }^{\text{max}})$. In the approximation (27), we have used the high-growth mass fractions $\phi_{i}^{hi}=B_{i}/\kappa_{i}+O(\epsilon )$ with the pathway weight $B_{i}≃B_{p(i)}=j_{p}^{hi}$, as given by (22), and the kinetic factors $c_{i}=\sqrt{K_{i}\kappa_{i}/B_{i}}$, equation (4), with the pathway average ${\bar{c}}_{p}\equiv \sum_{k\in p}c_{k}\phi_{k}^{hi}/\phi_{p}^{hi}$.

This broad-acting, environment-dependent metabolite cost differentiates our model from resource allocation models that predict homogeneous, flux-proportional resource allocation for all metabolic enzymes except uptake enzymes [27,43,47,71]: without a constraint on the over-expression of uptake enzymes, growth-optimal intracellular metabolite concentrations largely decouple from catalyzed flux and fluxes per unit mass remain approximately constant. In contrast, growth-optimal metabolite concentrations in our model are reduced even far downstream in response to nutrient limitation, resulting in broad, enzyme-specific resource reallocation. We focus here on a regime of intermediate growth-rate reductions, not exceeding 70% of the maximal growth rate, for which experimental data indicate well-defined metabolic resource reallocation [10,14] (Fig 3).

Next, we compute the joint growth response factors of enzyme levels $Q_{i}$ and metabolite levels $Q_{i}^{\rho }$ by combining the high-growth solution (21, 22) and the constitutive relations (17, 18) with the cost (27) (for simplicity, we assume the uptake enzymes remain at a small fraction of the pathway proteome, $\phi_{\nu }^{\max}\ll \phi_{p}$). As a first step, we express the local response factors $Q_{i}$ and $Q_{i}^{\rho }$ as functions of the net pathway factors $Q_{p}=\phi_{p}/\phi_{p}^{hi}$ and $Q_{p}^{v}\equiv v_{p}/v_{p}^{hi}$,


$$\begin{array}{ccc} Q_{i}(Q_{p},Q_{p}^{v}) & ≃ & Q_{p}\left(Q_{p}^{v}+\frac{c_{i}}{{\bar{c}}_{p}}\left(1-Q_{p}^{v}\right)\right), \end{array}$$ (28)

$$\begin{array}{ccc} Q_{i}^{\rho }(Q_{p}^{v}) & ≃ & \frac{Q_{p}^{v}\eta_{p}}{(1-Q_{p}^{v})+Q_{p}^{v}\eta_{p}}, \end{array}$$ (29)

In (29), we have used the metabolite mass fractions $\rho_{i}^{hi}=\sqrt{K_{i}B_{i}/\kappa_{i}}$ (as given by (17) without the position-dependent gradation), which sets the pathway metabolome-proteome mass ratio at high growth, $\eta_{p}\equiv \sum_{k\in p}\rho_{k}^{hi}/\phi_{p}^{hi}$. Matching the strong-constraint approximation ($\beta_{i}≃\beta_{0}$) with the high-growth metabolite levels (1) introduces the correction term $Q_{p}^{v}\eta_{p}$ in the denominator of (29).

### System-wide growth response

In a second step, we compute the differential resource allocation between pathways with different degrees of restriction, which yields an explicit relation between the response factors and the growth rate. To ensure homogeneous reduction of metabolic fluxes across the network, enzyme growth response has to be inversely proportional to efficiency response,


$$\begin{array}{c} \frac{j_{i}}{j_{i}^{hi}}=Q_{i}Q_{i}^{v}≃\frac{\lambda }{\lambda^{hi}}, \end{array}$$ (30)

which also implies $Q_{p}Q_{p}^{v}≃\lambda /\lambda^{hi}$. These relations are consistent with the global efficiency relation (12).

Importantly, the pathway response relations (28) and (29) and the global flux balance relation (30) afford a self-consistent closure that contains the main result of this study: generic, enzyme-specific linear growth response in a multi-pathway network. Specifically, we obtain


$$\begin{array}{cc} Q_{i}(\lambda ) & ≃\frac{\lambda }{\lambda^{hi}}+\frac{c_{i}}{{\bar{c}}_{p}}\left(Q_{p}(\lambda )-\frac{\lambda }{\lambda^{hi}}\right)\\ & =\frac{\lambda }{\lambda^{hi}}+\frac{c_{i}\xi_{p}}{\bar{c}}\left(1-\frac{\lambda }{\lambda^{hi}}\right) \end{array}$$ (31)

$$\begin{array}{cc} Q_{i}^{\rho }(\lambda ) & ≃\frac{\eta_{p}\lambda /\lambda^{hi}}{\eta_{p}\lambda /\lambda^{hi}+(Q_{p}(\lambda )-\lambda /\lambda^{hi})}\\ & ≃\frac{\eta \lambda /\lambda^{hi}}{\eta \lambda /\lambda^{hi}+\xi_{p}(1-\lambda /\lambda^{hi})}. \end{array}$$ (32)

In the last transformations we have used that, given the linearity of $Q_{i}(\lambda )$, the term $Q_{p}-\lambda /\lambda^{hi}$ must be proportional to $\left(1-\lambda /\lambda^{hi}\right)$. This proportionality introduces the restriction factors $\xi_{p}$, which can be defined by the relation


$$\begin{array}{c} \frac{{\bar{c}}_{p}}{\bar{c}}\;\xi_{p}=\frac{Q_{p}(\lambda )-\lambda /\lambda^{hi}}{1-\lambda /\lambda^{hi}} \end{array}$$ (33)

with the system-averaged kinetic factor


$$\begin{array}{c} \bar{c}\equiv \sum_{i}\phi_{i}^{hi}c_{i}\xi_{p(i)}. \end{array}$$ (34)

In (32), we have also used the rescaling $\eta /\bar{c}≃\eta_{p}/{\bar{c}}_{p}$. This follows from comparison of equations (24) and (34), using equations (17) and (22), up to pre-factors of order 1 related to position-dependent metabolite costs at high growth and unrestricted parts of the metabolic network.

The restriction factors (33) measure pathway-specific biases in growth response that cannot be accounted for by biases in reaction kinetics. From equation (27), we obtain an equivalent interpretation: restriction factors measure the pathway-specific excess metabolite cost induced by nutrient limitation, $\beta_{0,p}$, up to a global prefactor,


$$\begin{array}{c} \xi_{p}^{2}=\frac{{\bar{c}}^{2}\lambda /\lambda^{hi}}{(1-\lambda /\lambda^{hi}{)}^{2}}\;\beta_{0,p}. \end{array}$$ (35)

Here, the average kinetic factor $\bar{c}=O(\epsilon )$ sets the mass scale of metabolites in accordance with equations (21) and (24). Relative costs between pathways are independent of the growth rate and depend only on restriction factors, $\beta_{0,p}/\beta_{0,p^{\prime }}=\xi_{p}^{2}/\xi_{p^{\prime }}^{2}$. In the examples below and in our data analysis, the restriction factors depend only on broad restriction sectors, $\xi_{p}=\xi_{s(p)}$, which yields equations (3) and (5) of the main text.

In the simplest case of a uniformly restricted network, the restricted pathway *p* covers the entire network. This determines $\phi_{p}=1,B_{p}=\lambda^{hi}$, $Q_{p}^{v}=\lambda /\lambda^{hi}$ by equation (12), demonstrating enzyme-specific linear growth response for this system. This system is described by equations (28) and (29). These are equivalent to equations (3) and (5) with a system-wide restriction factor ξ=1, corresponding to a system-wide metabolite restriction cost $\beta_{0}\sim (1-\lambda /\lambda^{hi}{)}^{2}/(\epsilon^{2}\lambda /\lambda^{hi})$ by equation (35). The resulting resource reallocation patterns are shown in Figs 2, S2, and S5 together with exact analytical solutions for individual parameterizations (S1 Appendix). The minimal restriction model for nutrient limitation used in the main text is detailed in the next subsection. In S1 Appendix, we present a simplified derivation of linear proteome reallocation and discuss which model assumptions can be relaxed.

### Network model for differential nutrient uptake

In what follows, we apply our model to a simple branched network: two input pathways (*C* and *N*) process different nutrients, a focal reaction produces a compound metabolite from their respective outputs (following a Michaelis-Menten relation for both of its substrates; S1 Appendix), and a downstream pathway (*T*) further processes the compound metabolite (Figs 4 and S3D–S3F). This topology is a minimal model for a network with three functional sectors: enzymes depending directly on nutrient 1 (*C*), on nutrient 2 (*N*), and on both nutrients (*T*). An explicit solution is given in S1 Appendix.

The growth-dependent resource allocation between and within restriction sectors can be computed from this solution. Specifically, under limitation of nutrient 1, pathway *N* converts an abundant nutrient, which implies $\beta_{0,N}\sim \lambda$ via equations (10) and (16). Equations (16) and (18) then yield $\phi_{N}\sim \lambda$ and constant metabolite concentrations throughout the unrestricted sector in this model system. The junction enzyme, which links *N* with the restricted pathways *C* and *T*, exhibits Michaelis-Menten kinetics in both of its substrates and remains saturated in its substrate derived from nutrient 2. As a result, pathways *C* and *T* can be described as a continuous linear pathway *R* with single-substrate Michaelis-Menten kinetics. Together, the system-wide response pattern follows a sector-specific pattern, as given by equations (3) and (5) and can be consistently captured by just two broad restriction sectors: the restricted sector R=C∪T with $\xi_{R}=1$ and the unrestricted sector *U* = *N* with $\xi_{U}=0$. This solution supports the empirical classification of pathways into restriction sectors presented in the main text. The system-wide response pattern can be summarized as follows:

(i) The growth response factors of enzyme levels given in equation (31), $q_{i}\equiv Q_{i}(\lambda \;=\;0)$ follow a double gradation: they depend on the local kinetic parameter $c_{i}$ and on the restriction factor of the embedding sector *s*(*i*),
  $$\begin{array}{c} q_{i}=\frac{c_{i}}{\bar{c}}\xi_{s(i)}, \end{array}$$ (36)
  generating a fine-grained pattern that is heterogeneous within pathways and sectors. The kinetic parameters $c_{i}$ are defined in equation (4), the network average $\bar{c}$ is given by (34). We note that ratios within the same pathway depend only on the intra-pathway enzyme kinetics,
  $$\begin{array}{c} \frac{q_{i}}{q_{j}}=\frac{c_{i}}{c_{j}}=\frac{\sqrt{\kappa_{i}K_{i}}}{\sqrt{\kappa_{j}K_{j}}}, \end{array}$$ (37)
  because the common factors $B_{p}$ and $\xi_{s(p)}$ cancel.
(ii) The cognate growth response of enzyme-specific metabolic efficiencies, $Q_{i}^{v}(\lambda )$, follows a similar fine-grained pattern,
  $$\begin{array}{cc} Q_{i}^{v}(\lambda ) & =\frac{\lambda }{\lambda^{hi}}\;Q_{i}^{-1}(\lambda )\\ & ={\left(1+\frac{c_{i}}{\bar{c}}\xi_{s(i)}\left(\frac{\lambda^{hi}}{\lambda }-1\right)\right)}^{-1}. \end{array}$$ (38)
(iii) The restriction factors $\xi_{s}$ govern the growth response of metabolic efficiencies for all enzymes *i* in restriction sector *s*,
  $$\begin{array}{c} \frac{c_{i}}{\bar{c}}\xi_{s}=\frac{\lambda /\lambda^{hi}}{Q_{i}^{v}(\lambda )}\left(\frac{1-Q_{i}^{v}(\lambda )}{1-\lambda /\lambda^{hi}}\right). \end{array}$$ (39)
  This relation expresses the efficiency response, $Q_{i}^{v}(\lambda )$, in terms of sector-specific restriction, $\xi_{s}$, and local susceptibility to restriction, $c_{i}$. The restriction factors can self-consistently be expressed in terms of the enzyme response factors. Parameterizing $q_{i}=Q_{s}(c_{i})$ for all enzymes in sector *s*, we obtain
  $$\begin{array}{c} \xi_{s}=\bar{c}\;\frac{dQ_{s}(c)}{dc}. \end{array}$$ (40)
  That is, although the growth response factors follow a fine-grained pattern, the expressions (39) and (40) are constant within each restriction sector.
(iv) The growth response factors (36) of individual enzymes can be consistently aggregated over pathways and functional sectors. Here we define mass-weighted mean and rms pathway response factors by
  $$\begin{array}{ccc} {\bar{q}}_{p} & \equiv & \frac{\phi_{p}(\lambda \;=\;0)}{\phi_{p}^{hi}}=\sum_{i\in p}\frac{\phi_{i}^{hi}}{\phi_{p}^{hi}}q_{i},\\ \Delta_{p} & \equiv & (\sum_{i\in p}\frac{\phi_{i}^{hi}}{\phi_{p}^{hi}}(q_{i}-{\bar{q}}_{p}{)}^{2}{)}^{1/2}, \end{array}$$ (41)
  where $\phi_{p}(\lambda )=\sum_{i\in p}\phi_{i}(\lambda )$ is the growth-dependent aggregate mass fraction of pathway *p*. For pathways contained in a single restriction sector *s*(*p*), we obtain the double-graded growth response
  $$\begin{array}{ccc} {\bar{q}}_{p} & = & \frac{\xi_{s(p)}}{\bar{c}}\sum_{i\in p}\frac{\phi_{i}^{hi}}{\phi_{p}^{hi}}c_{i}, \end{array}$$ (42)
  $$\begin{array}{ccc} \Delta_{p} & = & \frac{\xi_{s(p)}}{\bar{c}}(\sum_{i\in p}\frac{\phi_{i}^{hi}}{\phi_{p}^{hi}}c_{i}^{2}-(\sum_{i\in p}\frac{\phi_{i}^{hi}}{\phi_{p}^{hi}}c_{i}{)}^{2}{)}^{1/2}. \end{array}$$ (43)
  The common dependence of ${\bar{q}}_{p}$ and $\Delta_{p}$ on the pathway weight factor $B_{p}$ and on the restriction factor $\xi_{s(p)}$ generates the correlation described by the third growth law, equation (8). The empirical pathway growth response factors ${\hat{q}}_{p}$ and ${\hat{\Delta }}_{p}$, defined in analogy to equations (41), are reported in Fig 4B and S1 Table. In line with equation (43), we use the intra-pathway response variation to classify pathways into restriction sectors (Fig 3). Sector-specific growth response factors, ${\bar{q}}_{s}$ and $\Delta_{s}$, and their empirical counterparts, ${\hat{q}}_{s}$ and ${\hat{\Delta }}_{s}$ are computed in the same way; the inferred values for functional sectors are shown in Fig 4B.
(v) To compute the growth-dependent resource allocation between restriction sectors, we evaluate the average kinetic parameter $\bar{c}=\phi_{R}^{hi}{\bar{c}}_{R}$ from equation (34). Equation (3) then predicts
  $$\begin{array}{c} {\bar{q}}_{R}=\frac{1}{\phi_{R}^{hi}},\;\;{\bar{q}}_{U}=0, \end{array}$$ (44)
  consistent with a broad resource shift from *U* to *R*.

### Proteomics data analysis

We analyze a proteomics dataset published by Mori et al. [14], which contains absolute proteome fractions of 1,647 *E. coli* proteins at six different carbon-limited growth rates induced by inhibition of glucose uptake in up to three replicates. Among all 217 annotated metabolic modules in the KEGG database [49] (accessed on September 1st, 2025), we identified all for which at least five proteins were consistently detected across all growth conditions (55 modules). To eliminate redundancy, we excluded strongly overlapping modules, resulting in 34 curated pathways. To assess whether protein expression scales linearly with growth rate, we fitted a linear model. Of 1,647 detected proteins, 549 were metabolic (belonging to the KEGG pathway *path:eco01100*, ’Metabolic pathways’); 516 (94%) of these were well described by the linear model within an error margin of three standard deviations. Overall, the distribution of residuals from linear fits was indistinguishable from the distribution of expression-fold differences between biological replicates (p≈0.2, Kolmogorov-Smirnov test). For each protein, we extracted the expression at high growth ($\lambda^{hi}=0.91\;/h$) and the protein growth response factor $\hat{q}$ under nutrient limitation from fit lines, using scipy’s linregress function. We note that $\hat{q}$ is given by the intercept of a linear fit to data obtained at intermediate levels of growth reduction. The actual expression at zero growth may differ due to non-linearities at very low growth rate (S3 Fig). Figs 1 and S1 show the growth responses of individual proteins stratified by pathway and normalized by the extracted expression at high growth. The sum of a pathway’s protein mass fractions evaluated at all measured growth rates was used to fit the pathway average ${\hat{q}}_{p}$ and its standard deviation. A linear fit to log-transformed fit values for individual proteins was used to infer *z* and its standard error in pathways (Figs 3 and S6). Outliers with poor linear fits or negative fit values $\hat{q}$ were excluded from these analyses. A detailed description is given in S1 Appendix. Response heterogeneity was analyzed by comparison of a homogeneous pathway response model (shared $\hat{q}$ among all pathway proteins) to an enzyme-specific response model (enzyme-specific fit of $\hat{q}$) using the Akaike Information Criterion [83] (AIC). Specifically, we computed AIC for each pathway (reported in S1 Table) as twice the difference in the sum of squared residuals between both models, corrected for the difference in the number of fit parameters. We further quantified both the mass-weighted mean response ${\hat{q}}_{p}$ of each pathway, and the mass-weighted standard deviation ${\hat{\Delta }}_{p}$. As predicted by our model, both quantities are significantly correlated, ${\hat{\Delta }}_{p}\sim ({\hat{q}}_{p}{)}^{x}$. Deviations between the empirical exponent, *x* = 0.67, and our model prediction of *x* = 1 for restricted pathways are explained by the unclear assignment of several analyzed pathways to restriction sectors, the resulting inclusion of unrestricted pathways and enzymes with dual functions in two sectors.

### Integrative analysis of proteome and metabolome data

We analyze measurements of absolute metabolite concentrations and absolute substrate affinities published by Bennett et al. [16]. We convert measurements to our unit system (S1 Appendix) and match published measurements with proteomics data. Of 549 metabolic proteins, substrate concentrations (excluding cofactor concentrations) were found for 221. In 9 of 34 pathways, metabolite concentrations were measured for at least five proteins. If more than one metabolite concentration was measured for a given enzyme, we used the geometric mean substrate saturation for further analysis. A linear fit to log-transformed data was used to infer *y* and its standard error in these pathways (Fig 3).

Expression data and metabolomics data used for inference of *y* and *z* in individual pathways were corrected for the respective pathway-specific geometric mean expression level, protein growth response, or metabolite saturation, and then aggregated into a common dataset to determine *p*-values for a power-law relation with nonzero exponent between these variables (S6 Fig).

### Natural selection generates correlations of kinetic parameters

Equations (12, 13), and (32) define a biophysical fitness landscape [78,84–89] for mutations affecting the kinetic parameters of a focal enzyme in a linear pathway that represents the entire cell,


$$\begin{array}{c} f(K_{i},\kappa_{i})\approx \frac{{\bar{j}}_{0}}{1+\rho_{i}^{⋆}(K_{i},\kappa_{i})+\sum_{k\neq i}^{\ell }\rho_{k}^{⋆}}, \end{array}$$ (45)

as shown in S4 Fig. Here, we vary only the kinetic parameters of one enzyme while keeping a fixed set of physiological kinetic parameters for the remainder of the pathway constant. Growth rate is used as a measure of fitness, and ${\bar{j}}_{0}$ denotes the long-term average nutrient supply flux, which we assume to be at intermediate rates below $\lambda^{hi}$. Selection coefficients associated with typical mutational changes affecting substrate binding or catalysis are given by $\varsigma_{i}=-(K_{i}/\lambda )(\partial \lambda /\partial K_{i})$ and ${\tilde{\varsigma }}_{i}=(\kappa_{i}/\lambda )(\partial \lambda /\partial \kappa_{i}){|}_{K_{i}/\kappa_{i}}$. We find


$$\begin{array}{cc} \varsigma_{i} & \sim \rho_{i}^{⋆}\sim \frac{\lambda }{\left(\lambda^{\text{max}}-\lambda \right)}\sqrt{\frac{K_{i}\lambda^{\text{max}}}{\kappa_{i}}}\\ {\tilde{\varsigma }}_{i} & \sim \frac{\lambda^{3}}{(\lambda^{\text{max}}-\lambda {)}^{2}}\frac{1}{\kappa_{i}} \end{array}$$ (46)

The selection coefficient associated with degrading substrate affinity, ς, is larger in enzymes with low catalytic constant κ (S4 Fig), explaining correlations in kinetic parameters. Typical selection coefficients on catalytic constants exceed those on substrate affinities at high nutrient supply but decrease more rapidly with decreasing supply ($\tilde{\varsigma }\sim \lambda^{3}$). At intermediate and low growth, selection on specificities dominates.

### Metabolite-mediated regulation can approximate growth-optimal resource reallocation

We construct a dynamical model of enzyme expression and metabolite turnover. Metabolite concentrations are governed by equation (13), fluxes are determined by Michaelis-Menten kinetics. Enzyme concentrations are governed by synthesis and dilution $d\phi_{i}/dt=b_{i}-\lambda \phi_{i}$, with synthesis rates $b_{i}$ determined by metabolite-mediated transcriptional regulation $b_{i}^{t}(\rho )$. For each enzyme, transcription is parameterized by a basal level $\phi_{i}^{0}$, regulatory amplitude $a_{i}$, and binding constant to a metabolite, *K*_reg_. Protein synthesis rates $b_{i}$ are given by $b_{i}^{t}$ normalized by total translational capacity, ensuring global resource competition and conservation of overall protein production at rate λ.

We explore two canonical regulatory motifs: feed-forward regulation, where each substrate metabolite controls the expression of its consuming enzyme, and feedback regulation, where the product metabolite regulates the preceding enzyme. For each motif, we evaluate the growth-optimal relation between metabolite and protein concentrations (S2 Fig, S1 Appendix) to determine a candidate basal transcription level, regulatory amplitude, and binding constant for each enzyme. We find regulatory parameters that depend only on local kinetic parameters. For feed-forward regulation, they are given by


$$\begin{array}{ccc} \phi_{i}^{0} & = & D_{i}\sqrt{K_{i}/\kappa_{i}} \end{array}$$ (47)

$$\begin{array}{ccc} a_{i} & = & E_{i}/\kappa_{i}-D_{i}\sqrt{K_{i}/\kappa_{i}} \end{array}$$ (48)

$$\begin{array}{ccc} K_{i}^{\text{reg}} & = & D_{i}\epsilon^{2}\sqrt{K_{i}/\kappa_{i}}. \end{array}$$ (49)

$D_{i}$, $E_{i}$ denote pathway-specific suitable normalizations (S1 Appendix). Rate-optimized enzymes ($E_{i}^{-1}K_{i}\kappa_{i}>Z^{2}$) are repressed, specificity-optimized enzymes ($E_{i}^{-1}K_{i}\kappa_{i}>Z^{2}$) are activated by their cognate metabolite, in accordance with equation (3). Details and parameter values for the feedback regulation motif are given in S1 Appendix.

We simulate the coupled dynamics of enzyme synthesis according to these regulatory functions, metabolite fluxes (13), and dilution using differential equations, with the cellular growth rate defined by flux through the terminal reaction. At any nutrient supply level, resource allocation settles to a stable stationary state that mimics growth-optimal resource allocation (Fig 5). Simulated nutrient supply levels correspond to $\lambda \approx \lambda^{hi}$, and growth reductions by 50% and 33% in this order.

## Supporting information

S1 Appendix Additional information. Construction of growth-optimal states, model comparison to experimental data, detailed description of the regulatory and evolutionary model, and comparison to related modeling approaches.
(PDF)

S1 TableEmpirical data of pathways and enzymes.Curated pathways (top) and sectors (bottom) of *E. coli* used in this study are listed by decreasing variance in log growth response ($\log\hat{q}$). From left to right: (1) Pathway name. (2) KEGG ID. (3) Enzymes measured in all growth conditions [14], ordered by decreasing $\hat{q}$ (top to bottom in Figs 1, 3, S1, and S6). (4) Mass-weighted average expression growth response, ${\hat{q}}_{p}$ (± standard error). (5) Standard deviation ${\hat{\Delta }}_{p}$ of intra-pathway response coefficients $\hat{q}$. (6) Fit exponent *y* (± standard error) of the empirical correlation between growth response and saturation at high growth (Figs 3 and S6), cf. equation (6). Power-laws are marked in bold face and shown in Fig 3 if the exponent differs by > 1.5 standard deviations from zero. (7) Fit exponent *z* (± standard error) of the empirical correlation between growth response and expression at high growth (Figs 3 and S6), cf. equation (7). Power-laws are marked in bold face and shown in Fig 3 if exponents differ by more than 1.5 standard deviations from zero. Outlier proteins excluded from fits are marked by a star (Methods). (8) Akaike Information Criterion [83] of the comparison between a joint linear response model for the entire pathway and an enzyme-specific linear response model. Negative values indicate that the data is best explained by an enzyme-specific response model, positive values indicate a homogeneous pathway response. (9) Sector association based on metabolic module association with Nitrogen metabolism (*N*), Terminal anabolism (*T*) or Carbon metabolism (*C*) according to the KEGG classification [49]. Terminal anabolism and carbon metabolism are restricted (*R*). Nitrogen metabolism and Sulfur metabolism are unrestricted (*U*). (10) Notes indicate pathways in specific classes (see main text): [**c**] (protein complex), [**o**] (operon), [**b-**] (kinetic parameter bias towards specificity-optimization), [**b+**] (kinetic parameter bias towards rate-optimization). Abbreviations: pathway (pw), biosynthesis (bs).(PDF)

S2 Table Table of variables and parameters. List of mathematical symbols, abbreviations, and notation together with their definitions.
(PDF)

S1 FigProtein expression changes under nutrient limitation in all analyzed pathways.(A) Relative expression changes under nutrient limitation sorted by metabolic pathway for all modules. Colored dots represent individual measurements. Linear fits to protein expression data are shown as solid lines if they explain the data within an error margin of three standard deviations on the root-mean-square deviation. Expression data from [14]. Pathways are individual modules as annotated in the KEGG database [49] for which at least five proteins were detected in all carbon-limitation conditions of the expression dataset. Modules were sorted by decreasing variance in $\log\hat{q}$. In addition to these metabolic modules, growth responses of ribosomal proteins are shown for comparison. Labels denote a subset of individual proteins, a complete list of proteins included for each pathway is given in S1 Table. (B) Distribution of expression deviations between biological replicates, and from different fit models. The distribution of deviations from a linear model matches the distribution between biological replicates. In contrast, a constant expression model does not explain the data and a quadratic model over-fits the data. *p*-Values were computed using a Kolmogorov-Smirnov test comparing the distribution of expression deviations from models to the distribution of expression deviations between biological replicates.(EPS)

S2 FigCorrelations between scaled metabolite and protein concentrations.Predicted scaling relation between metabolite and enzyme concentrations under nutrient-rich and nutrient-limited growth. Points show affinity-scaled mass fractions of simulated metabolite-enzyme pairs, $\left(\rho_{i}/K_{i},\phi_{i}/K_{i}\right)$, in growth-optimal metabolic states under high nutrient supply (green) and nutrient limitation (orange). These concentrations are determined by two key parameters (solid lines): the metabolite cost $\beta_{i}$ and the growth response parameter $c_{i}\sim (\kappa_{i}K_{i}{)}^{1/2}$, which reflects local enzyme kinetics (see S1 Appendix for precise definitions). At high nutrient supply, costs depend on network position *i*, leading to elevated concentrations of upstream metabolites (darker green shading), while protein expression levels are primarily shaped by local kinetic constants; see also S9A and S9B Figs. Nutrient limitation globally elevates metabolic costs, suppressing metabolite concentrations and inducing a heterogeneous protein response: high-*c* enzymes are up-regulated, low-*c* enzymes are down-regulated. Parameters: chain length ℓ=20; log-normally distributed kinetics with $\kappa =10^{1.6\pm 0.4}\;{\text{h}}^{-1}$ and $K=10^{-4.2\pm 1.1}$, $\phi_{0}^{\text{max}}\ll 1$. Points and grid lines for nutrient limitation correspond to dilution strength ϵ=0.05 (as given by equation (20), corresponding to metabolite mass fractions around 10% at high nutrient supply). Here, high nutrient supply refers to $\beta_{0}^{⋆}=0$, nutrient limitation ($\beta_{0}^{⋆}=40$) corresponds to a 50% growth rate reduction.(EPS)

S3 FigModel predictions for growth response of complex metabolic pathways.Left: schematic of pathway topology. Center left: protein expression levels, $\phi_{i}(\lambda )/\phi_{i}^{hi}$, as functions of the growth rate by enzyme class (rate-optimized, $c/\bar{c}>1$, or specificity-optimized, $c/\bar{c}<1$) and metabolic function. Here, $\lambda^{hi}$ denotes the maximal growth rate at high nutrient supply (eq. 23). Center right: metabolite levels, $\rho_{i}(\lambda )/\rho_{i}^{hi}$, as functions of the growth rate. Samples of individual enzymes and metabolites belonging to the pathway (dashed lines) are shown together with pathway averages (thick lines). Right: pathway-aggregate proteome and metabolome resource allocation relative to the proteome fraction of the pathway at $\lambda^{hi}$ (metabolites are upscaled by a factor 2 for better visibility). (A - C) Linear pathway under nutrient limitation. Linear, heterogeneous expression changes go together with a homogeneous, nonlinear change of metabolite levels (the example shown has ${\bar{c}}_{p}/\bar{c}<1$, leading to a net decrease of expression with growth). (D - F) Branched pathway with three sectors processing two nutrients. Sector *N* is rich (green), *C* and *T* are nutrient-limited (blue). The growth response of proteins and metabolites is sector-specific. (G - I) Cyclic pathway under nutrient limitation. The focal reaction *y* requires an input metabolite produced in reaction *y* + *k*. The model predicts a nonlinear expression increase of enzyme *y* with decreasing growth. Model parameters: The pathway examples shown have $\phi_{0}^{\text{max}}\ll 1$ and kinetic parameters as in S2 Fig with a bias towards specificity-optimized enzymes (${\bar{c}}_{p}=0.7\pm 0.1$). Network sizes: (A - C) $\ell_{\text{pw}}=10$ (pathway); ℓ=20 (embedding network). (D - F) $\ell_{\text{pw},A}=\ell_{\text{pw},C}=\ell_{\text{pw},N}=5$ (pathway); $\ell_{A}=\ell_{C}=\ell_{N}=10$ (embedding network). (G - I) *k* = 10 (pathway); ℓ=25, *y* = 11 (embedding network).(EPS)

S4 FigAffinity-rate trade-offs shape the growth laws.(A) Empirical affinity-rate trade-off. The physiological parameter distribution [51] is shown in blue, together with a linear fit to *K* as a function of κ with slope α≈0.2. (B) Average anti-correlation patterns between resource allocation at high nutrient supply, $\phi^{hi}$ and response *q* in simulated pathways with different affinity-rate trade-offs. Stochastic parameters as in S2 Fig and subsequently selected for the respective correlation pattern in kinetic parameters. (C) Predicted fitness landscape based on growth-optimal resource allocation for modifying the kinetic parameters of a single enzyme embedded in a physiologically parameterized 20-enzyme pathway. The physiological parameter distribution is shown in blue, its center of mass is marked by a dot and denotes the reference state with maximal growth rate $\lambda_{\text{ref}}^{hi}$. The landscape is shown for an intermediate median nutrient supply of ${\bar{j}}_{0}=\lambda_{\text{ref}}^{hi}/2$. Contour lines denote growth relative to the growth rate of a mutant with $\kappa_{i}\to \infty$. The arrow indicates the selective gradient on an enzyme with typical parameters. Selection acts on *K* and κ in a correlated manner. (D) Selection on the Michaelis-Menten constant of an enzyme with average $K_{i}$ as a function of its catalytic constant $\kappa_{i}$. An enzyme with higher catalytic constant experiences lower selection on *K*, explaining affinity-rate trade-offs.(EPS)

S5 FigAnalytical model predictions are applicable to growth-optimal responses in individual pathways.Five realizations (rows) of pathways of length ℓ=20, with $B_{p}=\lambda^{hi}$ and ξ=1; kinetic parameters drawn from log-normal distributions $\kappa =10^{1.6\pm 0.4}\;{\text{h}}^{-1}$, $K=10^{-4.2\pm 1.1}$ with correlation α≡Cov(logκ,logK)/Var(logκ)≈0.2. Left column: Growth-optimal response of metabolites. Center left: Growth-optimal response of proteins. Center right: Anticorrelation of protein growth response with saturation at high growth, $q_{i}\sim \sigma_{i}^{-y}$ (solid line: power-law fit). Right column: Anticorrelation of protein growth response with expression at high growth, $q_{i}\sim (\phi_{i}^{hi}{)}^{-z}$ (solid line: power-law fit).(EPS)

S6 FigEmpirical growth laws in all analyzed pathways.(A) Correlations between $\hat{q}=\phi^{lo}/\phi^{hi}$ and $\phi^{hi}$ as extracted from the fit-lines shown in S1 Fig. In a majority of metabolic modules, these data show a negative correlation (solid black line). Poor linear fits and fits intersecting at $\phi^{lo}<0$ were excluded. Red dots and fit lines indicate correlations between $\hat{q}$ and $\rho^{hi}/K$ in pathways for which metabolomic data were available. Power-law fits to the data of a given pathway are shown where the fit exponents indicate significant (anti-)correlations. Expression data from [14], metabolomics data from [16]. Pathways are individual modules as annotated in the KEGG database [49] for which at least five proteins were detected in all carbon-limitation conditions of the expression dataset. Modules were sorted by decreasing variance in $\log\hat{q}$. In addition to these metabolic modules, growth responses of ribosomal proteins are shown for comparison. A complete list of proteins included for each pathway is given in S1 Table. (B, C) Aggregated relative expression changes under nutrient limitation, corrected for the pathway mean growth response ${\hat{q}}_{p}$, as a function of $c/\bar{c}$ estimated from a protein’s pathway-mean-corrected saturation as $c\sim K/\rho^{hi}$ (B) or the protein’s pathway-mean-corrected expression in a nutrient-rich environment as $c\sim \sqrt{1/\phi^{hi}}$ (C). Pathways were included in the metabolome analysis if substrates of at least five proteins were quantified by metabolomics. *p*-Values denote the likelihood of obtaining a power-law fit with the given or more extreme slope under the null hypothesis of no correlation between protein growth response and either saturation (B) or expression (C) in a nutrient-rich environment. Points are colored by individual pathway. *N* denotes the total number of analyzed proteins. All metabolic pathways – including protein complexes, co-regulated operons and pathways not affected by the nutrient limitation – were included in this analysis.(EPS)

S7 FigExpression at low growth does not predict expression changes with increasing nutrient supply.Relative expression changes under nutrient limitation sorted by metabolic pathway. In comparison to the panels shown in Figs 3 and S6, $\phi^{hi}$ and $\phi^{lo}$ were exchanged. Each panel thus shows correlations between ${\hat{q}}^{-1}=\phi^{hi}/\phi^{lo}$ and $\phi^{lo}$ as extracted from the fit-lines shown in S1 Fig. Power-law fits to the data of a given pathway are shown where the fit exponents indicate significant (anti-)correlations. In a majority of metabolic modules, these data do not show a negative correlation. Expression data from [14].(EPS)

S8 FigMetabolite-mediated feedback regulation.Metabolites regulate the transcription of their producing enzymes, inducing variable levels of repression for rate-optimized enzymes and weakly specificity-optimized enzymes ($\theta c_{i}/\bar{c}≳1$) and activation for strongly specificity-optimized enzymes ($\theta c_{i}/\bar{c}≲1$). The regulatory logic of feedback regulation is illustrated in Fig 5B. (A) Time-dependent metabolite levels for feedback regulation in a linear pathway, $\rho_{i}(t)$ (gray) nutrient concentration (dashed), and regulated protein concentrations $\phi_{i}(t)$ (dark blue: specificity-optimized enzymes, $c/\bar{c}<1$; light blue: rate-optimized enzymes, $c/\bar{c}>1$, dashed: uptake enzyme) under time-dependent nutrient limitation, $\rho_{0}(t)$ (top, dashed). (B) Regulated late-time stationary levels, ${\bar{\rho }}_{i}$ and ${\bar{\phi }}_{i}$, plotted against growth-optimal levels. All panels use θ=5 to ensure convergence to the stationary state. For θ≤1, the regulatory scheme can maintain a close-to-optimal state, but does not converge to it. The uptake reaction and the final protein production reactions are under feed-forward control as described in the main text.(EPS)

S9 FigModel predictions on metabolite effects at high nutrient supply.(A) Specificity-scaled metabolite and enzyme catalytic constant-scaled protein levels, $\rho_{i}/s_{i}$ and $\phi_{i}/r_{i}$, as functions of relative network position, i/ℓ (with $r_{i}=1/\sum_{k}(\kappa_{i}/\kappa_{k})$). Points are individual enzyme-substrate pairs in physiologically parameterized pathways carrying the entire biosynthetic flux. Lines are analytical predictions of equations (20–22). (B) Position- and catalytic constant-scaled metabolite and protein levels, $\rho_{i}(\beta_{i}/r_{i}{)}^{1/2}$ and $\phi_{i}/r_{i}$, as functions of inverse substrate affinity, $K_{i}$. (C) Growth rate degradation, $\lambda /\lambda^{\text{max}}-1$ in comparison to the maximal growth rate expected from strict flux balance, $\lambda^{\text{max}}$, and metabolome-proteome mass ratio, $M_{R}/M_{P}$, as functions of the dilution strength ϵ. Points are complete, physiologically parameterized pathways carrying the entire biosynthetic flux evaluated at high nutrient supply. Stochastic parameters as in S2 Fig.(EPS)
