# Supplementary material for: Enzyme kinetics shapes the growth response of metabolic networks
Source: PLoS Comput Biol. 2026 Aug 19;22(8):e1014642. doi: 10.1371/journal.pcbi.1014642 (PMC13533438; doi:10.1371/journal.pcbi.1014642)
Supplement: Supplementary file 1 [file pcbi.1014642.s001.pdf]

Appendix to ‘Enzyme kinetics shapes the growth response of metabolic networks’

Leon Seeger[1], Fernanda Pinheiro[2], Michael Lässig[1*]

1 Institute for Biological Physics, University of Cologne, Cologne, Germany
2 Computational Biology Research Centre, Human Technopole, Milan, Italy

* mlaessig@uni-koeln.de

Maximization of balanced growth rate by resource allocation

Scaling of variables. Bacteria reallocate metabolic resources under environmental challenges. We explore growth-optimal resource allocation in a structured network of biochemically heterogeneous enzymes (Fig 1) in variable environments. In contrast to previous proteome sector models[26], our model concurrently accounts for both metabolites and enzymes. As a result, our model introduces second-order rate constants that describe reactions with multiple reaction partners, such as enzyme-substrate interactions. The rates and concentrations in our model exhibit a different scaling with the reaction volume when enzyme and substrate amounts are held constant. To include this scale in our model, we introduce the parameter

$$\nu \equiv \frac{V}{M_P}. \tag{50}$$

Here, V is the total intracellular volume, and M_P is the total mass of protein. Empirical observations suggest that this fraction remains approximately constant throughout the cell cycle and across different environments[21]. The parameter ν enables converting mass concentrations m/V into dimensionless mass fractions m/M_P. A metabolite mass fraction ρ is defined as $\rho = M_S \nu c$, with the classical concentration c, in units of $\mathrm{mol\,L^{-1}}$, and the molar mass of the metabolite M_S. For simplicity, we apply identical unit conversions to extracellular nutrient concentrations. Similarly, a protein mass fraction ϕ corresponds to $\phi = M_E \nu c_E$ with the classical concentration c_E (in units of $\mathrm{mol\,L^{-1}}$) and the molar mass of the protein M_E. In this unit system, the protein mass fractions in a cell of ℓ proteins must sum up to 1,

$$\sum_{i=1}^{\ell} \phi_i = 1. \tag{51}$$

Flux units are accordingly chosen to directly express the time derivatives of mass fractions as flux summations, $d\rho/dt = j_{\mathrm{production}} - j_{\mathrm{consumption}} - j_{\mathrm{dilution}}$. The conversion also extends to kinetic rate constants. For instance, a Michaelis-Menten reaction depicts an enzyme with a mass fraction ϕ consuming a metabolite ρ at a rate $j = \kappa\phi/(1 + K/\rho)$. Here K is a dimensionless Michaelis constant, linked to the classical Michaelis constant $\tilde{K}_M$ (in units of $\mathrm{mol\,L^{-1}}$) via $K = M_S \nu \tilde{K}_M$. κ is the enzyme's turnover number in this unit system and relates to the classical turnover number k_{cat} via $\kappa = (M_S/M_E) \cdot k_{\mathrm{cat}}$. In this unit system, the growth rate is compactly represented as the mass-normalized flux through the protein biosynthesis reaction,

$$\lambda \equiv \frac{1}{M_P}\frac{dM_P}{dt} = j_P. \tag{52}$$

Furthermore, we define the following extensive variables to simplify notation throughout: $R_i \equiv \rho_i M_P$, $M_i \equiv \phi_i M_P$, and $J_i \equiv j_i M_P$. This unit conversion allows us to write a compact and (apart from time) dimensionless derivation.

Irreversible Michaelis-Menten kinetics. The rate at which an enzyme-catalyzed reaction proceeds depends on the enzyme's concentration, its kinetic constants, and its substrate and product concentrations. If the reaction only proceeds through catalysis, its rate is proportional to the enzyme's concentration. Further, as catalysis does not affect the thermodynamic equilibrium between substrates and products, the rate must vanish at thermodynamic equilibrium. These properties are captured by

$$j = \phi f(\vec{\rho}) \left(1 - \exp \frac{\Delta G(\vec{\rho})}{k_B T}\right) \qquad \textbf{(53)}$$

with $\vec{\rho}$ denoting the concentrations of all metabolites participating in the reaction, and $\Delta G(\vec{\rho})$ the resulting change in Gibbs free energy [90]. The change in Gibbs free energy determines the magnitude of the reverse flux in comparison to the forward flux

$$j_{\text{reverse}} \approx j_{\text{forward}} \exp \frac{\Delta G(\vec{\rho})}{k_B T} \qquad \textbf{(54)}$$

Under physiological conditions, E. coli oxidizes about a third of its carbon uptake to generate energy [91]. Complete oxidation of glucose yields approximately 40 phosphorylations of ADP. The hydrolysis of each resulting ATP molecule dissipates an energy of about 10 $k_B T$. At typical pathway lengths of 20 reactions from carbon uptake to macromolecule synthesis (see below), and under the assumption that the Gibbs free energy of macromolecular structures is not markedly different from the Gibbs free energy of their precursors, this corresponds to a dissipation of about 10 $k_B T$ on average per conversion step - the cell operates strongly out of equilibrium to facilitate fast growth. Direct measurements of free energy differences are available for a few metabolic reactions, and indicate on average lower, but often still substantial deviations from thermodynamic equilibrium [92]. While reversibility and resulting thermodynamic constraints on metabolite concentrations affect individual reactions, we thus assume that most reactions in the cell are strongly driven out of equilibrium and can be described accurately as irreversible. To make $f(\vec{\rho})$ explicit, we here chose Michaelis-Menten kinetics in a single or two substrates to model individual, enzyme-catalyzed interconversion steps for their widespread use in enzyme biochemistry.

$$f(\rho) = \frac{\kappa}{\prod(1 + \frac{K}{\rho})}. \qquad \textbf{(55)}$$

Gibbs free energy differences are linear functions of the chemical potentials $\mu(\rho) \propto \log(\rho)$ of metabolites involved in the reaction. Below, we show that our model predicts that substrate and product concentrations change by a similar factor under nutrient limitation, leaving Gibbs free energy differences largely unaltered. This result is self-consistent with our assumption of strong thermodynamic disequilibrium even under nutrient limitation. Using Michaelis-Menten kinetics further allows us to integrate kinetic data [16,51], which is often reported using the same kinetic approximation. We use kinetic datasets for physiological parameterization of our model and model validation.

Independent variables: metabolite fluxes. We restrict our analysis to balanced growth states. This is exponentially growing, metabolic steady-states that obey metabolite mass conservation in each metabolic reaction. In balanced exponential growth (averaging over cell-cycle dependent and stochastic variation), no intracellular metabolite

concentration may change over time. Furthermore, all reactions must fulfill their kinetic rate law. As a result, only a specific subset of all possible metabolic states, denoted by $\mathcal{S}$, is viable during balanced growth. Given a network of enzymes with kinetic parameters κ and K, any metabolic state during balanced growth is fully determined by its metabolite fluxes J: Differences between metabolite production and consumption fluxes determine all dilution fluxes, and thereby metabolite concentrations. Kinetics, fluxes, and metabolite concentrations determine enzyme masses. We take the final flux j_ℓ of pathways and sub-networks to be proportional to growth rate with a proportionality constant γ. If the pathway represents the complete metabolic network and its final reaction represents protein biosynthesis, the proportionality constant is one. In a linear pathway of ℓ reactions, this relation can be made explicit by inserting $\lambda = J_P/M_P$ into the balanced growth condition, $(J_{i-1} - J_i)/M_P - \lambda\rho_i = 0$, and solving for ρ_i. All but external metabolite concentrations can be expressed as a function of the fluxes,

$$\rho_i(J) = \frac{\gamma(J_{i-1} - J_i)}{J_\ell} \qquad (i = 1, \ \ldots, \ \ell). \tag{56}$$

By summation over pathway steps starting from $i = \ell$, this yields

$$J_i = J_P\left(\gamma + \sum_{k=i+1}^{\ell} \rho_k\right). \tag{57}$$

Although the dilution flux of any single metabolite is minor compared with the enzyme-catalyzed flux through that metabolite, the summed dilution across the metabolome is appreciable. The fraction of flux diverted to dilution equals the cellular mass fraction of metabolites and is only about one order of magnitude smaller than the total protein-synthesis flux.[16,59,60] Similarly, on a per-component basis, a metabolite's dilution flux differs little from the corresponding enzyme's production flux, implying that dilution can influence growth almost as strongly as protein synthesis. We quantify this effect below.
We next compute each individual protein mass according to the kinetic rate laws. In case of a linear pathway, each reaction only has one substrate, and we find

$$M_i(J, K, \kappa) = \frac{J_i}{\kappa_i}\left(1 + \frac{K_i}{\rho_i(J)}\right). \tag{58}$$

By summation, the total protein mass of the pathway can easily be computed, added to the protein mass required for other cellular functions and production of the pathway's substrate $M_r(J_0)$, and subsequently R_i from ρ_i, ϕ_i from M_i, j from J. Taken together, the growth rate can be expressed as a function of all metabolic fluxes, the nutrient environment (setting $M_r(J_0)$), and the kinetic constants.

$$\lambda(J, K, \kappa) = \frac{\gamma J_\ell}{M_r(J_0) + \sum_{i=1}^{\ell} M_i(J, K, \kappa)}. \tag{59}$$

Optimality of metabolic states. Akin to flux-balance analysis[8], we employ constraints to identify physiological solutions. A key constraint is the constant intracellular protein density ν^{-1}, which is well established empirically[1,13,52–54]. Due to this constraint, we can identify a metabolic state $\mathcal{S}^\star$ that optimizes growth rate λ: if an enzyme's concentration is too low, it limits growth through a metabolic bottleneck. If its concentration is too high, it demands a reduction of other enzymes' concentrations, which also impairs growth.
We focus only on the regulatory problem of finding optimal metabolic states $\mathcal{S}^\star$ for a given set of kinetic constants (κ, K). We assume that kinetic constants evolve on a slower timescale, in contrast to simultaneous evolutionary optimization of kinetic

constants and expression levels. To shorten the notation, we drop the kinetic constants from all arguments below. Optimal balance growth states are defined as

$$\mathcal{S}^\star \equiv \arg\max(\lambda(\mathcal{S}))\Big|_{M_r(J_0),\ \frac{d\rho}{dt}=0}. \qquad (\mathbf{60})$$

As shown in the preceding paragraph, metabolic states that fulfill balanced growth and the kinetic rate laws are fully determined by their fluxes J (**59**). We thus rewrite the previous equation,

$$J^\star \equiv \arg\max(\lambda(J))\Big|_{M_r(J_0)}. \qquad (\mathbf{61})$$

By construction, this optimal state can grow exponentially with time-independent metabolite concentrations. For any system size M_P, at least one optimal state exists. We here set the system size implicitly by choosing a certain J_ℓ. Maximal growth rate is achieved if the protein mass necessary to produce this flux J_ℓ is minimal. Thus, we can simplify the optimality condition further and obtain Eq (**14**) - optimality is achieved only if

$$\frac{\partial M_r(J_0) + \sum_{k=1}^{\ell} M_k(J)}{\partial J_i}\Bigg|_{J^\star} = 0 \qquad (i = 0,\ \ldots,\ \ell - 1). \qquad (\mathbf{62})$$

This principle resembles Enzyme Cost Minimization[48], which has been used to numerically find growth rate maximizing resource allocation when considering reversible kinetic rate laws but no dilution of metabolites. Here, we instead focus on growth maximization in a model that explicitly takes metabolite dilution into account, but no reversibility. We proceed analytically. A restricted analysis of this kind was previously pursued by Ehrenberg and Kurland[40]. Beyond their analysis, we quantify the relative influences of differences in enzyme kinetics and network properties on resource allocation to individual enzymes and metabolites and analyze how these influences change under environmental challenges.

Iterative construction of the optimal metabolic states in longer pathways

Optimal resource allocation in coarse-grained metabolic models. A cell model consisting of just two proteome sectors ($\ell = 2$) - with catabolic enzymes M_1 and anabolic enzymes M_2 - has been employed successfully to infer proteome resource reallocation in response to changes in the nutrient concentration ρ_1 and the catalytic constant of the anabolic sector κ_2[21]. For such a short chain, the optimality condition (**61**) has also been solved analytically, including both the proteome and the metabolome[57].

Producing the first substrate of a pathway from a new precursor. Longer pathways feature many non-linearities and feedback loops, and can not easily be solved analytically anymore. Partially numerical solutions for pathways[40], mathematical properties of optimal solutions for metabolic networks[44,45], as well as an analytical solution of a pathway with two enzymes[57] were studied previously with a focus on the growth response of the ribosome. Here we start from a linear pathway, directly converting an environmental nutrient present at a fixed concentration ρ_0 into protein biomass (i.e. $\gamma = 1$). This nutrient concentration sets $M_r(J_0)$ according to Michaelis-Menten kinetics of the uptake reaction (Eq **58** for $i = 0$). To find the optimal metabolic states $\mathcal{S}^\star(J_\ell, \rho_0)$ for longer pathways, we identify an iterative scheme for constructing optimal metabolic

states of a chain with $\ell + 1$ enzymes from the optimal metabolic states of a chain with ℓ enzymes. Suppose the optimal metabolic state $\mathcal{S}^\star_\ell(J_\ell, \rho_0)$ is known for a given chain defined by its 2ℓ kinetic constants in an environment ρ_0. We extend the given chain by a single enzyme with its kinetic constants, a protein mass M'_{-1}, catalyzed flux J'_{-1}, and a new environmental precursor concentration ρ'_{-1} instead of ρ_0. The new enzyme produces the first substrate of the shorter chain (A1 Fig). To simplify the notation in this section, we shift all indices by 1. We further indicate variables of the extended chain by a dash $\cdot\ '$, and drop the superscript $\cdot^\star$. Below, all state variables denote their growth-optimal values.

Recursion relations for state variables. An extension that adds one additional flux J'_0 but conserves all fluxes of the shorter chain $J'_{i\geq 1} = J_i$, also conserves all downstream concentrations $\rho'_{i\geq 2} = \rho_{i\geq 2}$ and thus all downstream protein masses $M'_{i\geq 2} = M_{i\geq 2}$, according to Eqs (**56**) and (**58**). An extension of the chain by a single enzyme with arbitrary flux therefore only changes ρ_1 to a new ρ'_1 for which mass conservation now applies, correspondingly M_1 to a new M'_1, and adds ρ'_0, M'_0, and J'_0.
These five new or modified state variables are linked by five equations: two kinetic rate laws (Eq (**58**) for $i = 0$ and $i = 1$), a mass conservation constraint (Eq (**56**) for $i = 1$), and two conditions on optimality (Eq (**62**) for $k = 0$ and $k = 1$). These five equations can be solved for the five state variables. The solution is jointly given by

$$\begin{aligned}
\rho'_0 &= \frac{\frac{\kappa_1}{\kappa_0} K_0 \rho_1 \left(2\frac{J_1}{J_\ell}(K_1 - \frac{\kappa_1}{\kappa_0}\rho_1) + K_1(\rho_1 + \sqrt{4\rho_1 \frac{J_1}{J_\ell} + \rho_1^2}) \right)}{2\frac{J_1}{J_\ell}\left(K_1 - \frac{\kappa_1}{\kappa_0}\rho_1\right)^2 - 2\frac{\kappa_1}{\kappa_0} K_1 \rho_1^2}, \\
J'_0 &= J_1\left(1 + \sqrt{\frac{J_\ell}{J_1}\frac{\kappa_0}{\kappa_1}\frac{K_1}{(1 + \frac{K_0}{\rho'_0})}}\right), \\
\rho'_1 &= \frac{J'_0 - J_1}{J_\ell}, \\
M'_0 &= \frac{J'_0}{\kappa_0}(1 + \frac{K_0}{\rho'_0}), \\
M'_1 &= \frac{J_1}{\kappa_1}(1 + \frac{K_1}{\rho'_1}).
\end{aligned} \tag{63}$$

A single step of this iteration is illustrated in A1 Fig. All other conditions on optimality (Eq (**62**) for $k \geq 2$) are fulfilled by the extended chain if they were fulfilled by the shorter chain because the protein masses M'_0 and M'_1 do not depend on downstream fluxes $J'_{k\geq 2}$ in a system of irreversible enzymes ($\partial M'_0/\partial J'_{k\geq 2} = 0$, $\partial M'_1/\partial J'_{k\geq 2} = 0$), while the required protein mass downstream $\sum_{i=2}^{\ell} M_i$ only depends on downstream fluxes in a manner independent of the extension ($\partial \sum_{i=2}^{\ell} M_i/\partial J'_0 = 0$). Precisely this property of the model allows for a local construction of a new chain start that again yields an optimal chain, rather than requiring a full adjustment of the whole chain.

Direct iteration of fluxes. Computationally, it is practical to calculate the concentration of an external nutrient just once. Instead of iteratively determining the five modified state variables in each step, fluxes alone can be iterated according to the optimality criterion of Eq (**62**). To derive an explicit formula, we consider a variation of a single flux $J_i + \delta J_i$ (with $i = 1, \ \ldots, \ \ell - 1$), while keeping all other fluxes constant, as required by the optimality criterion. Among all mass conservation criteria (**56**), only those for ρ_i and ρ_{i+1} depend on J_i. We find $\rho_i + \delta\rho_i = (J_{i-1} - (J_i + \delta J_i))/J_\ell$ and $\rho_{i+1} + \delta\rho_{i+1} = (J_i + \delta J_i - J_{i+1})/J_\ell$. Together, these changes in metabolite concentrations and the change in flux affect enzyme masses according to the Michaelis-Menten

relation (**58**). Inserting the changes derived above yields mass changes under variation of a single flux around the optimal state,

$$\delta M_i = \frac{\delta J_i}{\kappa_i}\left(1+\frac{K_i}{\rho_i}\right)+\frac{J_i}{\kappa_i}\frac{K_i}{\rho_i^2}\frac{\delta J_i}{J_\ell}, \qquad \text{and} \qquad \delta M_{i+1} = -\frac{J_{i+1}}{\kappa_{i+1}}\frac{K_{i+1}}{\rho_{i+1}^2}\frac{\delta J_i}{J_\ell}. \qquad (\mathbf{64})$$

To summarize, for $i \geq 2$, only ρ_i and ρ_{i+1} (see Eq (**56**)) depend on J_i. Only M_i and M_{i+1} depend on either J_i, ρ_i or ρ_{i+1}. The respective changes are illustrated in A1C Fig. The optimality criterion (**62**) can now be drastically simplified to $(\partial(M_i(J,\rho_1)+M_{i+1}(J,\rho_1))/\partial J_i|_{J^\star,\rho_1} = 0$ for $i = 1, \ \ldots, \ \ell-1$. By inserting the derivatives (**64**), and expressing metabolite concentrations through fluxes (**56**), we find an iteration condition on fluxes,

$$J_{i-1}(J_i, J_{i+1}, J_\ell) = J_i + \frac{J_\ell K_i \kappa_{i+1} + \sqrt{J_\ell K_i \kappa_{i+1}\left(\frac{4 J_i J_\ell J_{i+1}\kappa_i K_{i+1}}{(J_i - J_{i+1})^2} + (J_\ell K_i - 4 J_i)\kappa_{i+1}\right)}}{\frac{2 J_\ell J_{i+1}\kappa_i K_{i+1}}{(J_i-J_{i+1})^2} - 2\kappa_{i+1}}. \qquad (\mathbf{65})$$

Note that we derive the remaining equations of the main text below. The result only depends on downstream fluxes $J_{m\geq i}$ and kinetic constants. From a starting condition J_ℓ and $J_{\ell-1}$, all fluxes in a chain can be determined iteratively. Only in the last iteration step, a matching nutrient concentration is computed. It is given analogously to Eq (**63**) by

$$\rho_0(J) = \frac{\frac{\kappa_1}{\kappa_0} K_0 \frac{J_0-J_1}{J_\ell}\left(2\frac{J_1}{J_\ell}\left(K_1 - \frac{\kappa_1}{\kappa_0}\frac{J_0-J_1}{J_\ell}\right) + K_1\left(\frac{J_0-J_1}{J_\ell} + \sqrt{4\frac{J_0-J_1}{J_\ell}\frac{J_1}{J_\ell} + \left(\frac{J_0-J_1}{J_\ell}\right)^2}\right)\right)}{2\frac{J_1}{J_\ell}\left(K_1 - \frac{\kappa_1}{\kappa_0}\frac{J_0-J_1}{J_\ell}\right)^2 - 2\frac{\kappa_1}{\kappa_0}K_1\left(\frac{J_0-J_1}{J_\ell}\right)^2}. \qquad (\mathbf{66})$$

At this point, Eqs (**56**) and (**58**) can again be used to compute all other metabolic state variables of $\mathcal{S}^\star$.

Matching the environmental nutrient concentration. Starting from a minimal chain that consists of only two enzymes, and all its possible optimal metabolic states $\mathcal{S}_2^\star(\rho_0, J_1)$, we can construct optimal metabolic states of a three-enzyme chain. By shifting indices, we can iteratively determine optimal metabolic states $\mathcal{S}_\ell^\star(\rho_0, J_\ell)$ for any longer chain.
In many biologically relevant scenarios, a boundary condition to be fulfilled is the environmental precursor concentration ρ_0. However, we here construct a suitable environmental nutrient concentration ρ_0 iteratively from the environmental nutrient concentration of the initial two-enzyme chain, to maintain the same mass requirement for increasing the flux through the first enzyme in a chain of two. It can easily be seen from Eq (**63**) that if any positive ρ_0 was possible for the shorter chain, any positive ρ_0' can be constructed for the elongated chain. Thus, the iterative method we present here can construct an optimal resource allocation for any environmental nutrient concentration. Unfortunately, it is not clear from the outset of the iteration, which environmental precursor concentration of the two-enzyme chain yields the environmental precursor concentration for the ℓ-enzyme-chain set by the biological scenario of interest. We solve this question numerically whenever a defined nutrient environment is required.

Uniqueness of optimal resource allocation. Any local extremum of growth rate found in this manner is a global optimum because the solution of the chain of two enzymes contains all optima of the chain of two enzymes. In each step, only one

physically meaningful solution (with all metabolic state variables larger than zero) of the set of five equations solved for the extension of the chain exists. Thus, the optimal states $J^\star$ determined here are indeed the only sets of fluxes that fulfill Eq (**62**). No physiological growth maxima exist at extremal values of any J_i. Consequently, the optimal metabolic state $\mathcal{S}^\star_\ell(\rho_0, J_\ell)$ is unique, and all optimal metabolic states of linear pathways can be constructed by the iterative method.

Local properties of optimal resource allocation

Metabolite costs increase along the metabolic chain. Above we have derived an analytical solution for optimal metabolic states of pathways, which represent the entire metabolic network - converting environmental nutrients into protein biomass. We next show how optimal resource allocation in an arbitrary linear pathway relates to the individual enzyme-substrate pair. To this end, we switch from a variation of a single flux J_i, which we used to construct growth-optimal balanced growth states above, to a variation of a single metabolite concentration $\rho_i + \delta\rho_i$. The optimality criterion (**14**) takes the form

$$\Big(\frac{\partial M_r(J_0)}{\partial J_0}\frac{\partial J_0}{\partial \rho_i} + \sum_{k=1}^{i-1}\frac{\partial M_k(J_k,\rho_k,\kappa,K)}{\partial J_k}\frac{\partial J_k}{\partial \rho_i} + \frac{\partial M_i(J_i,\rho_i,\kappa,K)}{\partial \rho_i}\Big)\Big|_{J_\ell,\rho^\star} = 0 \qquad (\mathbf{67})$$

We again derive the mass changes under such a variation. Eq (**57**) connects metabolite concentrations and fluxes in balanced growth states. It indicates that a variation of a single flux can be constructed if all fluxes upstream of reaction i are increased by a small δJ. We insert all changes in fluxes into the Michaelis-Menten relationship to obtain changes in enzyme masses,

$$\delta J_{k<i} = J_P \delta\rho_i, \qquad \text{and} \qquad \delta M_{k<i} = \frac{J_P M_k \delta\rho_i}{J_k}. \qquad (\mathbf{68})$$

This variation is illustrated in A1D Fig for the simplified case discussed above. Here we implicitly assumed that the uptake flux J_0 can be increased to balance the additional dilution flux $J_P\delta\rho_i$. This may in general not be true under nutrient limitation. We discuss the effect of nutrient limitation in more detail below. The focal reaction is also affected by the variation in ρ_i - not by a change in flux, but by the change in substrate concentration. We will investigate δM_i in more detail below. For a constant protein production flux J_ℓ, the total protein mass in the cell must also be minimal under variation of a single metabolite concentration (that is under a linear combination of changes in fluxes (**68**)). We simplify based on the changes in masses derived above to obtain

$$\left(\frac{\partial M_r(J_0)}{\partial J_0} J_P + \sum_{k=1}^{i-1}\frac{M_k}{J_k} J_P + \frac{\partial M_i(J_i,\rho_i,\kappa,K)}{\partial \rho_i}\right)\Bigg|_{J_\ell,\rho^\star} = 0, \qquad (\mathbf{69})$$

where $\partial M_k/\partial J_k = 1/v_k$ by the Michaelis-Menten relation (**9**) and $\partial J_k/\partial \rho_i = \lambda M_P$ by mass conservation (**57**). Thus, a small increase in concentration of the focal metabolite, $\delta\rho_i > 0$, is generated by an expression decrease of its cognate enzyme, $\delta M_i < 0$, and an increase of all upstream enzymes, $\delta M_k > 0$ for $k < i$. All metabolic currents $J_i, J_{i+1}, \ \ldots, \ J_\ell$ and all masses $M_{i+1}, \ \ldots, \ M_\ell$ remain constant to first order. In the optimal state, $M_P^\star$ must be a constant to first-order approximation in any of the variations discussed here. We can therefore equivalently write

$$\frac{\partial}{\partial \rho_i}\left(\beta_i^\star \rho_i + \phi_i(j_i,\rho_i,\kappa,K)\right)\Bigg|_{j_i^\star} = 0, \qquad \text{with} \qquad \beta_i^\star = \beta_0 + \sum_{k=1}^{i-1}\frac{\lambda\phi_k^\star}{j_k^\star}. \qquad (\mathbf{70}\text{a, b})$$

$\beta_0 \equiv (\lambda/M_P)\ (\partial M_r(M_P j_0)/\partial j_0)$ reflects the mass cost of increasing j_0 and depends on the growth environment and the remaining metabolic network. Eq (**70**) corresponds to Eq (**15**). We define the constant β_0 explicitly as independent of the variation in ρ_i. We obtain a result similar to the minimization of the joint mass concentration of an enzyme and its substrate at constant flux proposed by Dourado et al.[46], $\left(\frac{\partial}{\partial \rho}\right)_J(\rho + \phi) = 0$, which explains experimentally observed relations between enzyme and substrate mass concentrations. In contrast to Dourado et al. we here minimize the total *protein* mass at a fixed *protein* production flux. Our optimization directly follows from growth-optimality: In balanced exponential growth, each constituent of the cell must double at the same rate. Applied to protein, this means that growth rate is given by the protein production flux J_ℓ divided by the total protein mass M_P of the cell. At fixed J_ℓ, M_P must be minimal to achieve optimality. From the outset, it is not clear how such a minimization of the protein mass relates to the metabolite level. We here show that a joint minimization of protein and weighted metabolite mass follows from the optimization of growth rate. The weight $\beta_i^\star$ must be derived for a given metabolic network, and is generally different from 1. If j_0 describes an unconstrained uptake reaction of an extracellular nutrient ρ_ν, the cost β_0 becomes λ/v_ν, which approximates the proteome fraction of the uptake enzyme, ϕ_ν. For reactions situated deeper within the metabolic network and under nutrient-limited conditions, this cost reflects the influence of the global metabolic network and nutrient environment on the local balance between substrate and enzyme masses. We retrace the derivation of the scaling law discovered by Dourado et al.[46] with the additional cost $\beta_i^\star$. Eq (**58**) can be rewritten intensively as $\phi_i = (j_i/\kappa_i)\cdot(1 + K_i/\rho_i)$, and inserted into Eq (**70**a). Evaluating the derivative and rearranging yields Eq (**18**), shown in S2 Fig, and by re-inserting into Eq (**58**), we find Eq (**17**).

$$\rho_i^\star = \sqrt{\frac{K_i}{\kappa_i}}\sqrt{\frac{j_i^\star}{\beta_i^\star}}, \tag{71}$$

$$\phi_i^\star = \beta_i^\star \rho_i^\star \left(1 + \frac{\rho_i^\star}{K_i}\right) \qquad (i = 1,\ \ldots,\ \ell). \tag{72}$$

Physiological model parameters

Growth costs of metabolites and dilution strength ϵ. We next consider the impact of dilution on the systems level. To this end, we again consider the pathway as a simplified representation of the complete metabolic network. In this case, we can use the analytical solution derived above (**63**) for any given set of kinetic parameters. Beyond such explicit solutions, we here derive patterns of resource allocation below, which apply broadly, and are valid beyond individual parameterizations. The central scaling relations (**71**) and (**72**) must ensure that all proteome mass fractions sum to one. This property can be used to establish a parameter scale, which distinguishes between physiological parameterizations that approximately obey a production-consumption balance in each reaction and non-physiological parameterizations that are dominated by dilution. We insert Eq (**71**) into (**72**) and solve for $\phi_i^\star$,

$$\phi_i^\star = \frac{j_i^\star}{\kappa_i} + \beta_i^\star \rho_i^\star. \tag{73}$$

Summing over all proteome mass fractions (**51**), and inserting mass conservation (**57**), we derive

$$1 = \sum_{i=0}^{\ell} \phi_i^\star = \sum_{i=0}^{\ell} \frac{\lambda^\star (1 + \sum_{k=i+1}^{\ell} \rho_k^\star)}{\kappa_i} + \sum_{i=1}^{\ell} \beta_i^\star \rho_i^\star. \tag{74}$$

	$\tilde{K}_M$ *	k_{cat} *	$K = M_S \nu \tilde{K}_M$	$\kappa = \frac{M_S}{M_E} k_{\text{cat}}$
All enzymes	$10^{2.0\pm1.2}$ μM	$10^{1.1\pm1.4}$ s^{-1}	-	-
E. coli	$10^{2.0\pm1.1}$ μM	$10^{1.1\pm1.3}$ s^{-1}	$10^{-4.2\pm1.1}$	$10^{2.1\pm1.3}$ h^{-1}
Model cell	-	-	$10^{-4.2\pm1.1}$	$10^{1.6\pm0.4}$ h^{-1} **

Table A1. Distributions of enzyme kinetic parameters
To convert to the described unit system, we estimate $M_S \approx 10^2\,\text{g}\,\text{mol}^{-1}$, $\nu \approx 6\,\text{mL}\,\text{g}^{-1}$ and $M_E \approx 3.6 \cdot 10^4\,\text{g}\,\text{mol}^{-1}$. We parameterize our model according to these distributions, assuming that K and κ are independent random variables. Experimental data indicates that $\tilde{K}_M$ and k_{cat} are correlated [51]. Correlated distributions derived from rescaled data reported by ref. [51] are illustrated in S4 Fig.
*: based on data from Bar-Even et al. [51].
**: reduced mean and variance used in simulations to account for the reduced number of enzymes in the model, see below.

We insert the definition of metabolite costs (**70**b) (again in the nutrient-rich regime, where $\beta_i = \sum_{k=0}^{i-1} \lambda\phi_k/j_k$) to derive $\sum_{i=1}^{\ell} \beta_i^\star \rho_i^\star > \sum_{i=0}^{\ell} \frac{\lambda^\star}{\kappa_i} \sum_{k=i+1}^{\ell} \rho_k^\star$, and establish two bounds on growth rate,

$$1 > \sum_{i=0}^{\ell} \frac{\lambda^\star}{\kappa_i} > 1 - 2\sum_{i=1}^{\ell} \beta_i^\star \rho_i^\star. \qquad (\mathbf{75})$$

Growth rate must be smaller than the inverse of the sum of all inverse catalytic constants, termed $\lambda^{\max}$, which would be the growth rate if all enzymes could work at maximal saturation without dilution losses. At the same time, scaled growth rate is reduced by less than $2\sum_{i=1}^{\ell} \beta_i^\star \rho_i^\star$. By employing again the definition of metabolite costs, and $1 - \sum_{i=1}^{\ell} \beta_i^\star \rho_i^\star > \sum_{i=0}^{\ell} \frac{\lambda^\star}{\kappa_i}$ (**74**), we find that ϵ (eq. **20**) is an upper bound for $\sum_{i=1}^{\ell} \beta_i \rho_i$. Below, we show that physiologically parameterized cells exhibit a small value of $\epsilon \approx 10^{-1.3}$. Thus, their growth rate in nutrient-rich environments is predominantly determined by catalytic constants.

Physiological kinetic parameters of enzymes. To estimate the value of ϵ we use available data for kinetic rate constants. Bar-Even et al. [51] found that enzyme kinetic parameters across numerous species tend to follow a log-normal distribution. Restricting their analysis to *E. coli* enzymes yields distributions of similar mean and variance (see A1 Table). We leverage their results to approximate K and κ distributions for model parameterization. To convert units as described above, we estimate the average molecular weight of substrates M_S based on the molecular weight of glucose and amino acids (BNID: 104877); the volume per mass ratio ν (**50**) as the inverse of the protein mass per volume density in *E. coli* computed from the product of the dry mass protein fraction of *E. coli* (BNID: 101955) and its dry mass density (BNID: 109049); and the average protein mass M_E using the average protein length (BNID: 108986) and the average molecular weight of amino acids in *E. coli* (BNID: 104877). We disregarded any molar mass variability for substrates and enzymes on the grounds they are minor relative to the intrinsic variation of the kinetic parameters $\tilde{K}_M$ and k_{cat} and potentially correlated with one another and/or kinetic parameters.

Variability and central metabolism dynamics. In comparison to a real cell, our models incorporate a reduced spectrum of enzyme types, namely those of a single pathway converting environmental nutrients to new protein biomass. In a real cell, enzymes unaccounted here convert metabolites through parallel, interconnected pathways and generate a variety of intermediary metabolites. In addition to a larger variety in protein precursors, these pathways also synthesize other cellular biomass components such as

nucleic acids and lipids. Given the large spectrum of enzymes, the average proteome mass fraction of an individual enzyme type remains small. No singular enzyme mass fraction takes over the entire proteome - neither in nutrient-rich environments nor under nutrient limitation - despite large variability in their kinetic constants.

To achieve physiological growth rates and prevent a singular enzyme from completely dominating mass allocation in our model, we use a distribution of catalytic constants κ and binding constants K with lower mean and variance in comparison to the values found by Bar-Even et al. This choice addresses the reduced spectrum of enzyme types and is further motivated by evolutionary arguments: when used to represent the whole cell, our model roughly corresponds to central metabolism, which covers the conversion of environmental nutrients into new protein biomass. Central metabolism enzymes likely undergo more stringent selection than enzymes in secondary metabolism, resulting in decreased variance in their catalytic constants. In contrast, we maintained the natural variance in substrate affinities and thereby maintained a near-physiological variance in enzyme qualities.

To estimate the chain length of the metabolic network in a living cell, we focus on the conversion of an environmental carbon source into new protein biomass. This pathway likely contributes most to the overall protein biomass production of the cell. Environmental carbon sources are converted into different amino acids via different pathways. To estimate the physiological pathway length, we use the production of glutamine from glucose, which consists of 17 enzymatic steps connecting the following metabolites:

Glucose → Glucose-6-phosphate → Fructose-6-phosphate → Fructose-1,6-biphosphate → GADP/DHAP → 1,3-Biphosphoglycerate → 3-Phosphoglycerate → 2-Phosphoglycerate → Phosphoenolpyruvate → Pyruvate → Oxaloacetate/Acetyl-CoA → Citrate → Isocitrate → 2-Oxo-glutarate → Glutamate → Glutamine → Glutamineacyl-tRNA → Protein biomass

Other amino acid biosynthesis pathways include fewer (e.g. Serine), equally many (e.g. threonine), or more enzymatic steps (e.g. arginine). Alternative carbon sources may require a different number of enzymatic steps to produce pyruvate. Some enzymes may require additional modifications after protein synthesis. We here use $\ell \approx 20$ as an order of magnitude estimate for growth in amino-acid-free growth media.

Stochastic model parameterization. While kinetic properties of individual enzymes become increasingly available, only a minority has been measured to date. Additionally, enzymes with multiple substrates or otherwise complicated reaction kinetics may not exhibit a clearly defined substrate affinity. For these reasons, we do not parameterize our model based on available data for the specific enzymes. Instead, we parameterize our model stochastically, drawing both substrate affinities and kinetic constants independently from the log-normal distributions given in A1 Table. We thereby also disregard any bias in biochemical qualities along the metabolic cascade in our model.

Parameter estimation for the ribosome. Ribosomes exhibit a particularly high molecular mass ($\sim 10^6\,\mathrm{Da}$) but only an average catalytic rate constant $k_{\mathrm{cat}} \approx 20\,\mathrm{aa\,s^{-1}}$[1]. Together with the average molecular weight of amino acids of $M_S \approx 10^2\,\mathrm{Da}$, these properties yield a distinctly low catalytic constant of $\kappa = 10^{0.9}\,\mathrm{h^{-1}}$. Furthermore, they operate close to their maximal speed in the nutrient-rich regime[67,68] despite typical aminoacyl-tRNA concentrations in the µM-concentration regime (BNID: 105275). We therefore estimate a strong ribosomal substrate affinity $K^{-1} \sim 10^5$. Both properties are

the sign of a specificity-optimized protein. We estimate the predicted growth response of the ribosome as $c_{\mathrm{Rib.}}/\bar{c} \approx \sqrt{\kappa_{Rib.} K_{\mathrm{Rib.}}/\langle \kappa K \rangle}$, using the system-wide average kinetic constants stated in A1 Table. This yields $q_{\mathrm{Rib.}} \approx 10^{-1}$. As outlined further in the main text, we hypothesize that the ribosome is part of a coherently regulated group of specificity-optimized enzymes that lie downstream of most nutrient limitations.

Environmental nutrient concentrations. Amino-acid-free laboratory media for bacterial growth such as M9 typically contain between $0.1\,\mathrm{g\,L^{-1}}$ and $1\,\mathrm{g\,L^{-1}}$ glucose. The glucose concentration $\tilde{K}_{\mathrm{Monod}}$ at which the growth rate of *E. coli* is half-maximal is on the order of $1\,\mathrm{mg\,L^{-1}}$ (BNID: 111048). We here assume - and later show that this assumption is in line with our model's predictions - that the environmental nutrient concentration at which growth is half-maximal is on the order of the substrate affinity of the nutrient uptake enzyme, $\tilde{K}_{\mathrm{Monod}} \approx \tilde{K}_1$. This suggests that nutrient uptake enzymes are typically highly saturated in nutrient-rich laboratory media. Based on these typical values, we specifically use $\rho_1 = 1000\,K_1$ to describe nutrient-rich media. This value reflects all properties of the limit $\rho_1 \to \infty$ well but is numerically more easily accessible than the limit itself.

Global properties of optimal resource allocation

Physiological enzyme saturation. We compute the inactive proteome fraction ϕ_{inactive} (i.e. enzymes not currently engaged in catalysis due to a lack of substrates) as $\phi_{\mathrm{inactive}} \equiv \sum_{i=1}^{\ell} \phi_i^\star (1 - v_i/\kappa_i)$. Inserting the definition of v_i and Eq (**72**), this yields

$$\phi_{\mathrm{inactive}} = \sum_{i=1}^{\ell} \beta_i^\star \rho_i^\star < \epsilon \qquad \Big(\frac{\rho_0}{K_0} \gtrsim 1\Big). \tag{76}$$

Enzymes of physiologically parameterized cells in nutrient-rich environments are typically highly saturated - on average to a level larger than $1 - \epsilon$. Our model thus explains solely from the physiological distribution of enzyme kinetic parameters, why enzymes are typically well-saturated in nutrient-rich environments[16].

The cellular metabolite mass fraction in the homogeneous chain. Consider a homogeneous chain (which consists of enzymes with equal kinetic constants κ and K only) in the nutrient-rich regime. In the physiological parameter regime, such a chain fulfills $\epsilon \le \sqrt{\ell K} \ll 1$. In the limit of small dilution strength ϵ, Eqs (**56**), (**58**), (**70**b), (**71**), and (**72**) are then jointly solved by

$$\begin{aligned}
\rho_i^\star &\approx 0 + \epsilon\sqrt{\frac{1}{\ell i}} + \mathcal{O}(\epsilon^2),\\
\phi_i^\star &\approx \frac{1}{\ell}(1 + \epsilon\Big(\frac{2}{3} - \sqrt{\frac{i}{\ell}}\Big) + \mathcal{O}(\epsilon^2)),\\
j_i^\star &\approx \frac{\kappa}{\ell}(1 + \epsilon\Big(\frac{2}{3} - 2\sqrt{\frac{i}{\ell}}\Big) + \mathcal{O}(\epsilon^2)),\\
\beta_i^\star &\approx \frac{i}{\ell}(1 + \epsilon\Big(\frac{2}{3}\sqrt{\frac{i}{\ell}} - \frac{4}{3}\Big) + \mathcal{O}(\epsilon^2)),\\
\lambda^\star &\approx \frac{\kappa}{\ell}(1 - \epsilon\frac{4}{3} + \mathcal{O}(\epsilon^2)),\\
\epsilon &\approx \sqrt{\ell K}.
\end{aligned} \tag{77}$$

We here use $\sum_{k=i+1}^{\ell} k^{-1/2} \approx 2(\sqrt{\ell}-\sqrt{i})$ and $\sum_{i=1}^{\ell}\sqrt{i} \approx 2\ell\sqrt{\ell}/3$, which are motivated by the corresponding integrals. With these approximations, equations (**77**) form a consistent solution of microbial resource allocation to first order in ϵ, and the limit of vanishing ϵ constitutes the limit of vanishing dilution fluxes. In such a homogeneous chain, we can then compute the cellular mass fraction as $M_R/M_P = (j_0 - j_\ell)/j_\ell \approx 2\epsilon$ (**24**), which is itself on the order of ϵ. At the same time, the growth effect of metabolite dilution is estimated (**77**) to be on the order of $4\epsilon/3$, which stems approximately equally from proteome resources required for unsaturated enzymes and proteome resources required to balance metabolite dilution fluxes (**75**).

Resource allocation in heterogeneous chains. To extend our analysis to heterogeneous chains, we replace position by the continuous variable β, and sums over position by integrals over $\ell d\beta$. For a compact notation, we define the relative specificity s_β,

$$s_\beta \equiv \frac{\sqrt{\frac{K_\beta}{\kappa_\beta}}}{\ell \int_0^1 \sqrt{\frac{K_{\beta'}}{\kappa_{\beta'}}}\ d\beta'}. \tag{78}$$

The central equations for ρ, j and ϕ then yield a self-consistent solution even in heterogeneous chains (under the additional assumptions that β is not correlated with other variables and fluctuations in other variables average out efficiently even across small intervals in β - i.e. any integral $\ell \int_L s_\beta d\beta \approx L$):

$$\begin{aligned}
\rho_i^\star &\simeq \epsilon \frac{s_i}{\sqrt{\beta_i}}, \\
\phi_i^\star &\simeq \frac{\lambda}{\kappa_i}\Big(1 + 2\epsilon(1-\sqrt{\beta_i})\Big) + \epsilon s_i \sqrt{\beta_i}, \\
\lambda^\star &\simeq \Big(\sum_{k=0}^{\ell} \frac{1}{\kappa_i}\Big)^{-1} (1 - \frac{4}{3}\epsilon) \qquad \Big(\frac{\rho_\nu}{K_\nu} \gtrsim 1\Big).
\end{aligned} \tag{79}$$

In the limit of $\epsilon \to 0$, our model reproduces the result of standard flux-balance analysis: fluxes are conserved throughout the network ($j_i = \lambda$), all enzymes run at full saturation with rates κ_i and optimal expression levels proportional to $(1/\kappa_i)$, and metabolites are neglected ($\rho_i = 0$). The effect of metabolite dilution is given by a first-order expansion in ϵ. For sufficiently small variations of individual catalytic constants and affinities around their respective mean, this derivation – together with the mean-field approximation for β_i (**77**) – provides a reasonable mean-field approximation of heterogeneous chains (Figs S9 and A4). Systematic deviations introduced by physiological enzyme heterogeneity are small, as well as typical stochastic deviations. We note, however, that this is not true for all heterogeneous parameterizations in the case of the cellular metabolite mass fraction. In heterogeneous chains, no upper limit on the metabolite mass fraction can be established: if the nutrient uptake enzyme has an exceptionally high catalytic constant, its proteome fraction nearly vanishes. Then, the metabolite cost $\beta_1^\star$ nearly vanishes, and $\rho_1^\star$ can reach arbitrary values, which on their own may exceed 2ϵ drastically.

Comparison to experimental data. We compute several observables regarding the whole cell under the assumption of optimal resource allocation in our model. For physiological kinetic parameter distributions, they are in good agreement with those observed empirically (A2 Fig), apart from a slight overestimation of the cellular metabolite mass fraction. The metabolic resource allocation patterns identified here are generic and independent of the mean of κ. They are also largely independent of the variance in κ, as long as individual enzymes do not dominate the proteome. Overall, higher catalytic

constants solely lead to higher growth rates and fluxes. Higher values of K, on the other hand, lead to higher dilution relative to growth, creating more drastic flux gradients. This would also result in stronger position-dependent gradients in optimal metabolite saturation.

Optimal balanced growth states under nutrient limitation

In the case of a metabolic pathway embedded in a metabolic network, we have already posited that a proteome mass requirement $M_r(J_0)$ governs the metabolite cost of increasing the uptake flux J_0, resulting in a cost term β_0. Here we explore how such an additional cost arises if $M_r(J_0)$ indeed represents a nutrient uptake reaction from the environment – as in the case discussed above in which the pathway represents the entire metabolic network.

To this end, we consider a constraint on the nutrient uptake flux from the environment (which was left unconstrained in the nutrient-rich regime, and jointly optimized with the rest of the metabolic state of the cell), next to the constraint on the cellular protein concentration. We then identify the metabolic state that maximizes growth rate at the given nutrient uptake flux. In the absence of such a constraint on nutrient uptake flux at low nutrient concentrations, the complete calculus presented above would remain valid at low nutrient concentrations. Metabolite production costs for all intracellular metabolites would remain on the order of 1, and all $\phi_{i\geq 1}$ would converge to zero with decreasing flux j_0 according to Eqs (**72**), (**71**) and $j_i < j_0$. This implies that the uptake protein would become a macroscopic fraction of the total proteome, and eventually the whole proteome. At the same time, other proteome fractions would respond homogeneously in a flux-proportional manner, as predicted by several existing models[27,43,47,71]. Instead, for the reasons outlined in the main text, we specifically assume that uptake protein expression is constrained and thereby limits the nutrient uptake flux. Consider any constraint on $\phi_0(\rho_0)$. To find optimal resource allocation under the constraint, we use the method of Lagrange multipliers,

$$\mathcal{L}(J, \rho_0, \mu) \equiv M_P(J, \rho_0) + \mu\left(\frac{M_0(J, \rho_0)}{M_P(J, \rho_0)} - \phi_0(\rho_0)\right), \qquad (80)$$

with a Lagrange multiplier μ. The optimal resource allocation must now fulfill $\partial\mathcal{L}/\partial J_i|_{J^\star,\rho_1} = 0$ for $i = 0, \ \ldots, \ \ell{-}1$, in analogy to Eq (**62**), and additionally $\partial\mathcal{L}/\partial\mu = 0$. For $i \geq 1$ the uptake mass M_0 does not depend on J_i, and the partial derivatives are

$$\frac{\partial\mathcal{L}}{\partial J_i} = (1 - \phi_0\frac{\mu}{M_P})\frac{\partial M_P}{\partial J_i} \qquad (i = 1, \ \ldots, \ \ell - 1). \qquad (81)$$

They are zero in the optimal state if Eq (**62**) is fulfilled. Thus, the iterative computation of optimal fluxes (**65**) is still valid. $J^\star$ and all $M_i^\star$ for i in $(1, \ \ldots, \ \ell)$ follow from the iterative computation of optimal fluxes. A different boundary condition ρ_0 now matches a certain starting condition of the iteration $(J_\ell^\star, \ J_{\ell-1}^\star)$ in comparison to unconstrained optimality. For a given constraint $\phi_0(\rho_0)$, this boundary condition can be found by solving

$$J_0^\star = \frac{1}{1 - \phi_0(\rho_0)} \sum_{k=1}^{\ell} M_k^\star \frac{\kappa_0\phi_0(\rho_0)}{1 + \frac{K_0}{\rho_0}} \qquad (82)$$

for ρ_0, which corresponds to solving $\partial\mathcal{L}/\partial\mu = 0$. The last remaining condition on optimality is $\partial\mathcal{L}/\partial J_0|_{J^\star,\rho_0} = 0$, which could be solved for the remaining undetermined variable μ. However, μ is not required for describing the optimal state, and equations given above (**65**, **82**) fully suffice to compute optimal balanced growth states under nutrient limitation. The iterative construction of optimal balanced growth states is applicable under nutrient uptake constraints.

Metabolite costs under nutrient limitation. To put this result into the context of metabolite costs, we again consider the variation of a single metabolite concentration (and drop the superscripts $\cdot^\star$ below). Based on Eq (**57**), we derive

$$j_i = j_1 \frac{1+\sum_{k=i+1}^{\ell}\rho_k}{1+\sum_{k=1}^{\ell}\rho_k}. \tag{83}$$

This means that under a constraint on nutrient uptake, which defines a fixed flux $j_1(\rho_1)$ in a given nutrient environment, variation of a single metabolite concentration violates growth optimality, $\partial j_\ell/\partial\rho_k \neq 0$. A variation of a single metabolite concentration that maintains all other metabolite concentrations is ill-defined. Instead, we reformulate the optimality condition. We observe that in an optimal balanced growth state under a nutrient uptake constraint, the sum of metabolite concentrations derived from the growth-limiting nutrient must be minimal to maximize growth (**83**). This criterion can be reformulated further: all variations of any single metabolite concentration must have the same effect on the sum of proteome mass fractions $\sum_{k=1}^{\ell}\phi_k$,

$$\frac{\partial\sum_{k=1}^{\ell}\phi_k}{\partial\rho_i} = \frac{\partial\sum_{k=1}^{\ell}\phi_k}{\partial\rho_{i'}} \qquad (i,i'=1,\ \ldots,\ \ell). \tag{84}$$

To verify, suppose that this statement was incorrect in a growth-optimal state. A joint variation of ρ_i and $\rho_{i'}$ by the same $\delta\rho$ in opposite directions could reduce the required sum of proteome fractions. Adding the freed proteome fraction to any reaction would reduce dilution fluxes and thereby improve growth rate at fixed nutrient uptake.
We insert the kinetic growth law $\phi_k(j_k,\rho_k,\kappa_k,K_k)$, and find

$$\frac{\partial\sum_{k=1}^{\ell}\phi_k}{\partial\rho_i} = \left.\frac{\partial\phi_i}{\partial\rho_i}\right|_{j_i} + \sum_{k=1}^{\ell}\left(\frac{\phi_k}{j_k}\frac{\partial j_k}{\partial\rho_i}\right). \tag{85}$$

We insert (**83**) and subtract Eq (**84**) for $i'=1$. By comparison with equation (**69**) we can directly identify β_0,

$$0 = \left.\frac{\partial\phi_i}{\partial\rho_i}\right|_{j_i} + \beta_0^\star + \sum_{k=1}^{i-1}\frac{\lambda\phi_k}{j_k} \qquad \text{with} \qquad \beta_0^\star \equiv -\left.\frac{\partial\phi_0}{\partial\rho_0}\right|_{j_1}. \tag{86}$$

This additional cost associated with nutrient uptake affects all costs of metabolites derived from the growth-limiting nutrient equally as an additive constant. We obtain the functional form $\beta_i^\star = \beta_0^\star(\rho_0) + \sum_{k=1}^{i-1}\lambda^\star\phi_k^\star/j_k^\star$, which is given in Eq (**15**).
Again, local metabolite production costs are not suitable for determining optimal resource allocation, as they depend on the optimal resource allocation towards other reactions. Instead, we use them to interpret patterns in optimal resource allocation. We therefore do not solve for $\beta_0^\star$ analytically, but simply identify the additional summand as an arbitrary offset, which depends on the nutrient environment and the nutrient uptake constraint. Under variation of ρ_0, we biologically expect to find a one-parametric family of optimal states that fulfill the constraint.
Instead of characterizing optimal states by their nutrient concentration ρ_0, we choose to characterize them by their growth rate $\lambda^\star$. $\beta_0^\star$ is an arbitrary function of $\lambda^\star$ in this scenario. We can analogously to the case of unconstrained optimality derive $\rho_i^\star = \arg\min((\beta_i^{\mathrm{rich}} + \beta_0^\star(\lambda^\star))\rho_i + \phi(\rho_i,J_i))|_{J_i,\beta_i^{\mathrm{rich}},\beta_0^\star}$. Here, β_i^{rich} denotes the expression found for $\beta_i^\star$ in the nutrient-rich regime ($\beta_i^{\mathrm{rich}} \equiv \sum_{k=0}^{i-1}((\lambda^\star\phi_k^\star)/j_k^\star)$). The shape of the equation, including an additive $\beta_0^\star$, is independent of the exact nature of the constraint limiting nutrient uptake, as long as it only affects the flux j_0. Notably, a

diffusive constraint on nutrient uptake leads to the same result: in this case, the mass required for increasing the nutrient uptake flux j_0 would be larger than $\delta j_0/v_0$, because the local nutrient concentration at the cell surface would decrease with increasing j_0. This also yields an additional cost $\beta_0^\star$, which affects all reactions equally. Based on this argument, we here assume that the additional cost $\beta_0^\star$ vanishes at high nutrient supply. At high nutrient supply, the balance between diffusion and uptake has a vanishingly small effect on the concentration at the cell surface. Additionally, uptake protein expression was observed to increase under nutrient limitation, making it unlikely that the uptake protein expression is constrained below its unconstrained-optimal value in nutrient-rich environments. The effect of a regulatory nutrient uptake constraint on uptake expression, growth, and metabolite cost is illustrated in A3 Fig.

The limit of vanishing nutrient uptake. Above, we derived that a nutrient uptake limitation affects all following reactions equally by an additive cost component $\beta_0^\star$. Solving for $\beta_0^\star(\rho_0)$ for a given uptake constraint $\phi_0(\rho_0)$ is challenging due to the same feedback loops concerning optimal balanced growth states discussed earlier. However, its form as an additive constant allows for a simplified computation based on the summation constraint on proteome fractions,

$$1 = \sum_{i=0}^{\ell} \phi_i^\star = \phi_0(\rho_0) + \sum_{i=1}^{\ell} \left(\frac{j_i^\star}{\kappa_i} + (\beta_i^{\text{rich}} + \beta_0^\star)\rho_i^\star \right). \tag{87}$$

In the limit of strong nutrient limitation $j_0 \to 0$, this constraint can be solved directly for $\beta_0^\star(j_0)$: By Eq (**57**), all fluxes fulfill $j_i < j_0$ and vanish in the limit. Thus, each ϕ_i is well approximated by the linear term $((\beta_i^{\text{rich}} + \beta_0^\star)\rho_i^\star)$ of Eq (**72**). We denote $\lim_{j_1 \to 0} \phi_0$ with $\phi_0^{\max}$. By inserting Eqs (**71**) and (**86**), we find

$$1 - \phi_0^{\max} = \lim_{j_0 \to 0} \left(\sum_{i=1}^{\ell} \sqrt{\frac{K_i}{\kappa_i}} \sqrt{j_i^\star(\beta_0^\star + \sum_{k=1}^{i-1} \frac{\lambda^\star}{v_k})} \right). \tag{88}$$

For the reasons outlined in the main text, we assume that the uptake enzyme never constitutes the whole biosynthetic proteome; even in the limit of vanishing j_0, there is $\phi_0^{\max} < 1$. Similarly, we assume based on available experimental data that a pathway's expression does not vanish even as its catalyzed flux vanishes, $M_r(J_0) < M_P$. Thus, the right-hand side must also remain finite in the limit. For a given j_0, growth is maximized if the metabolite mass fraction M_R/M_P is minimal (**83**). We show below by construction that $M_R/M_P \to 0$ in the limit of $j_0 \to 0$. We expand in small j_0 and drop all terms of order j_0. We specifically use $j_i = \gamma_i j_0 + \mathcal{O}(j_0^2)$, and that all terms $\beta_i^{\text{rich}} = \sum \lambda^\star/v_k < \sum \phi_k^\star \leq 1$ remain finite in the limit,

$$1 - \phi_0^{\max} = \lim_{j_0 \to 0} \left(\sum_{i=1}^{\ell} \sqrt{\frac{\gamma_i K_i}{\kappa_i}} \sqrt{j_0 \beta_0^\star} \right). \tag{89}$$

Under a strong constraint on nutrient uptake, $\sqrt{j_0\beta_0^\star}$ must therefore converge to a suitable normalization constant Z, and $\beta_0^\star$ must diverge as $Z^2 \cdot j_0^{-1}$. This means that

resource allocation in the limit of vanishing nutrient uptake is given by

$$\begin{aligned}
\lim_{j_0\to 0}\phi_i^\star &= \sqrt{\frac{\gamma_i K_i}{\kappa_i}}Z,\\
\rho_i^\star &\approx \frac{j_0}{Z}\sqrt{\gamma_i\frac{K_i}{\kappa_i}}(1+\mathcal{O}(j_0)), \qquad\qquad (\mathbf{90})\\
j_i^\star &\approx \gamma_i j_0(1+\mathcal{O}(j_0)) \qquad \text{with } Z=\frac{(1-\phi_0^{\max})}{\sum_{i=1}^{\ell}\sqrt{\frac{\gamma_i K_i}{\kappa_i}}} \qquad \text{for } (i=1,\ \ldots,\ \ell).
\end{aligned}$$

As assumed above, metabolite concentrations vanish in the limit of vanishing nutrient uptake. In this limit, optimal proteome fractions are no longer set by catalytic constants, but by enzyme specificities.

Expansion to small nutrient uptake flux. An optimal balanced growth state in the limit of vanishing nutrient uptake is experimentally unattainable. For this reason, we compute optimal balanced growth states at small but finite growth rates by a first-order expansion of Eq (**87**) in small $j_0 = \lambda^\star(1+\mathcal{O}(\epsilon))$,

$$1-\phi_0(\lambda^\star)-\sum_{i=1}^{\ell}\frac{\gamma_i\lambda^\star}{\kappa_i}(1+\mathcal{O}(\epsilon)) = \sum_{i=1}^{\ell}\sqrt{\frac{\gamma_i K_i}{\kappa_i}}\sqrt{\lambda^\star\beta_0^\star}(1+\mathcal{O}(\epsilon))(1+\frac{\beta_i^{\text{rich}}}{2\beta_0^\star}). \qquad (\mathbf{91})$$

As long as $\beta_i^{\text{rich}} \ll \beta_0^\star$, we obtain

$$\sqrt{\beta_0^\star} \approx \frac{1-\phi_0(\rho_0)-\sum_{i=1}^{\ell}\frac{\gamma_i\lambda^\star}{\kappa_i}(1+\mathcal{O}(\epsilon))}{\sum_{i=1}^{\ell}\sqrt{\frac{\lambda^\star\gamma_i K_i}{\kappa_i}}(1+\mathcal{O}(\epsilon))}. \qquad (\mathbf{92})$$

We specifically find for ρ_i by Eq (**71**), and for ϕ_i by Eq (**87**),

$$\rho_i^\star \approx \lambda^\star\sqrt{\frac{\gamma_i K_i}{\kappa_i}}\frac{\sum_{i=1}^{\ell}\sqrt{\frac{\gamma_i K_i}{\kappa_i}}(1+\mathcal{O}(\epsilon))}{1-\phi_0(\rho_0)-\sum_{i=1}^{\ell}\frac{\gamma_i\lambda^\star}{\kappa_i}(1+\mathcal{O}(\epsilon))}, \qquad (\mathbf{93})$$

$$\phi_i^\star \approx \frac{\gamma_i\lambda^\star}{\kappa_i}(1+\mathcal{O}(\epsilon)) + \frac{\sqrt{\frac{\gamma_i K_i}{\kappa_i}}\left(1-\phi_0(\rho_0)-\sum_{i=1}^{\ell}\frac{\gamma_i\lambda^\star}{\kappa_i}(1+\mathcal{O}(\epsilon))\right)}{\sum_{i=1}^{\ell}\sqrt{\frac{\gamma_i K_i}{\kappa_i}}(1+\mathcal{O}(\epsilon))}. \qquad (\mathbf{94})$$

Proteome mass fractions are approximately linear in growth rate in the limit of strong nutrient limitation (apart from non-linearities introduced by $\phi_0(\rho_0)$, and metabolite dilution terms of order ϵ) and converge to well-defined values as derived above. Correction terms are of order $\mathcal{O}(\epsilon)$ in Eqs (**93**) and (**94**). For physiological parameter values, they can thus be neglected even at higher growth rates, and the approximation above remains valid as long as $\beta_i^\star \approx \beta_0^\star$. For this, $\beta_0^\star$ must be larger than all β_i^{rich}. We estimate based on Eq (**92**), and the definition of ϵ, $\sqrt{\beta_0^\star}-1 \geq (1-\phi_0(\rho_0)-\sum_{i=1}^{\ell}\gamma_i\lambda^\star/\kappa_i)/\epsilon$. Position-dependent metabolite production costs β_i^{rich} do not exceed 1. If the constraint on ϕ_0 is stringent enough to ensure $\phi_0 < 1-\sum_{i=1}^{\ell}\frac{\gamma_i\lambda}{\kappa_i}-2\epsilon$, the added cost exceeds 1. Thus, proteome fractions are approximately linear in growth rate at growth rates

$$\lambda^\star < \lambda^{\max}(1-\phi_0^{\max}-2\epsilon). \qquad (\mathbf{95})$$

This constitutes the boundary between the nutrient-rich and the nutrient-poor regime. The boundary translates approximately into $\rho_0/K_0 \simeq 1$: Above the boundary, the catalyzed flux through the first reaction is largely unaffected by reductions of ρ_0, and the

growth rate is approximately $\lambda^{\max}$. Below $\rho_0/K_0 \simeq \phi_0^\infty/\phi_0^{\max}$, ϕ_0 is not up-regulated meaningfully further, a property discussed in more detail in the section on regulatory constraints. Then, any further reduction in ρ_0 will lead to a near-proportional reduction in λ, and β_0 quickly exceeds one. The approximate equivalence of $\lambda \simeq \lambda^{\max}(1-\phi_0^{\max}-2\epsilon)$ with $\rho_0/K_0 \simeq 1$ was further verified numerically (A2F Fig).
Analogously, this argument holds for pathways embedded in more complex metabolic networks. In this case, $M_r(J_0)/M_P$ takes the position of ϕ_0. As long as $1 - M_r(J_0)/M_P)$ remains finite in the limit of low growth, the approximations above remain valid.

Linearity of expression level changes. Under physiological conditions, both $\phi_0^{\max}$ and ϵ are typically small. Neglecting both, an even stronger approximation can be made to simplify proteome fractions as a function of growth rate (**94**),

$$\phi_i^\star \approx \frac{\gamma_i \lambda}{\kappa_i} + \left(1 - \frac{\lambda}{\lambda^{\max}}\right) \frac{\sqrt{\frac{\gamma_i K_i}{\kappa_i}}}{\sum_{i=1}^{\ell} \sqrt{\frac{\gamma_i K_i}{\kappa_i}}}, \tag{96}$$

which is a linear function of the growth rate for each enzyme. We normalize by the expression level in the nutrient-rich regime $\phi_i \approx \lambda^{\max}/\kappa_i + \mathcal{O}(\epsilon)$, and identify

$$c_i \equiv \sqrt{\frac{K_i \kappa_i}{\gamma_i \lambda^{\max}}}, \qquad \text{with } \bar{c} \equiv \frac{\sum_{i=1}^{\ell} \frac{\gamma_i \lambda^{\max}}{\kappa_i} \sqrt{\frac{\kappa_i K_i}{\gamma_i \lambda^{\max}}}}{\sum_{i=1}^{\ell} \frac{\gamma_i \lambda^{\max}}{\kappa_i}}, \tag{97}$$

Inserting this definition of c and the approximate maximal growth rate for negligible ϕ_0 and ϵ, $\lambda^{\max} \approx (\sum_{k=1}^{\ell} 1/\kappa_i)^{-1}$, we find that an enzyme's biochemical quality c_i directly determines the optimal relative expression changes between a nutrient-rich environment and the limit of vanishing nutrient availability,

$$\frac{\phi_i^\star(\lambda)}{\phi_i^\star(\lambda^{\max})} \approx \frac{c_i}{\bar{c}} + \left(1 - \frac{c_i}{\bar{c}}\right) \frac{\lambda}{\lambda^{\max}}. \tag{98}$$

In a chain of irreversible Michaelis-Menten reactions with physiological parameters, any constraint that restricts the uptake proteome fraction from becoming a macroscopic fraction of the proteome under nutrient limitation leads to an optimal resource allocation pattern, approximately linear in growth rate for all enzymes except the uptake enzyme. Enzymes with $c/\bar{c} < 1$ are down-regulated upon nutrient limitation, and enzymes with $c/\bar{c} > 1$ are up-regulated. If a finite ϕ_0 is to be taken into account – or in embedded pathways, for which $\phi_r \equiv M_r(J_0)/M_P$ takes the role of ϕ_0 – the critical value of $c/\bar{c}$ that differentiates between up- and down-regulation shifts to $c_C/\bar{c} \approx 1 + \phi_0^{\max}$. Further non-linear corrections arise at high growth rates due to finite ϵ and, in particular, at very high c, due to the lowered substrate saturation in the nutrient-rich regime (S2 Fig). However, these corrections are typically small in the physiological parameter regime (S3 Fig). In embedded pathways, non-linearities in the growth response of ϕ_r can, in principle, introduce non-linearities to the growth response of individual pathway constituents. Based on experimental data[10,14], we assume that resource allocation to functional proteome sectors is commonly linear in growth rate. Below, we further explore this assumption on a theoretical basis by considering more complex metabolic networks.

Linear growth responses arise from metabolite concentration minimization

Above, we have shown that optimal growth responses of individual enzymes in an isolated pathway of Michaelis-Menten enzymes with metabolite dilution are approximately linear

in the physiological parameter regime. We have further derived a self-consistent closure of linear growth responses in complex metabolic networks in the main text. We here give an alternative, simplified derivation that illustrates the mechanisms by which linear growth responses arise despite the non-linearity of enzyme kinetics and metabolite responses, and which shows that alternative model assumptions can also yield linear proteome reallocation.
We start from enzyme mass minimization[48] with environment-dependent cost of metabolite dilution (**70**). To keep the following derivation concise, we here use the set of proteome fractions ϕ as independent variables, instead of ρ, and express the metabolite concentrations as functions of the enzyme concentrations at fixed flux,

$$\frac{\partial}{\partial \phi_i}(\beta_i \rho_i(\phi_i) + \phi_i) = \frac{\partial}{\partial \phi_{i'}}(\beta_{i'} \rho_{i'}(\phi_{i'}) + \phi_{i'}). \quad (\mathbf{99})$$

We show above, that a cell must maximize the conversion efficiency of available nutrients under strong nutrient limitation. This means that growth maximization under a strong constraint on nutrient uptake is equivalent to a minimization of metabolite loss and a minimization of the metabolite mass fraction ρ_i under the constraint of a fixed intracellular protein density. At low growth, dilution fluxes vanish, and the flux along the metabolic pathway is approximately constant, $j_i \approx \gamma_i \lambda$ – variation of a protein concentration only affects its own substrate concentration. This corresponds to $\beta_0(\lambda) \gg 1$.

Based on equation (**35**), we assume that metabolite costs in affected pathways increase in the same manner, $\beta_0^s = \xi_s^2 \beta_0(\lambda)$, but we allow for different restriction factors ξ_s between different proteome sectors. This assumption is further motivated by the fact that metabolite costs in different sectors stem from the same nutrient restriction, i.e. the same Lagrange multiplier. For example, $\xi_s \in \{1, 0\}$ can represent whether a pathway's metabolites are fully derived from the growth-limiting nutrient or not.

Inserting Michaelis-Menten kinetics (**9**) yields the simplified optimality condition

$$\frac{\partial}{\partial \phi_i} \frac{\xi_{s(i)}^2 \gamma_{p(i)} \lambda K_i}{\kappa_i(\phi_i - \frac{\gamma_{p(i)}\lambda}{\kappa_i})} = \frac{\partial}{\partial \phi_{i'}} \frac{\xi_{s(i')}^2 \gamma_{p(i')} \lambda K_{i'}}{\kappa_{i'}(\phi_{i'} - \frac{\gamma_{p(i')}\lambda}{\kappa_{i'}})}. \quad (\mathbf{100})$$

To minimize metabolite loss, allocating any additional proteome mass to reaction i must reduce the substrate concentration ρ_i by the same amount that $\rho_{i'}$ is increased if the same proteome mass is taken from reaction i' within the same sector. Between sectors, different restriction factors ξ_s modulate this balance. We define $c(\lambda) \equiv -\frac{\partial}{\partial \phi_1} \frac{\xi_{s(1)}^2 \gamma_{p(1)} \lambda K_1}{\kappa_1(\phi_1 - \frac{\gamma_{p(1)}\lambda}{\kappa_1})}$ and solve for the optimal proteome fraction,

$$\phi_i(\lambda) = \frac{\gamma_{p(i)}\lambda}{\kappa_i} + \xi_{s(i)} \sqrt{\frac{\gamma_{p(i)} \lambda K_i}{\kappa_i c(\lambda)}}. \quad (\mathbf{101})$$

Optimal proteome fractions consist of a flux-balancing proteome fraction j_i/κ_i and a metabolite-concentration minimizing fraction $\xi_{s(i)} \sqrt{\frac{\gamma_{p(i)} \lambda K_i}{\kappa_i c(\lambda)}}$. As all metabolites share the same growth-dependence - modulated by different restriction factors ξ_s - the summation constraint $\sum_i \phi_i = 1$ across the entire cell yields

$$1 = \frac{\lambda}{\lambda^{\max}} + \sum_i \xi_{s(i)} \sqrt{\frac{\gamma_{p(i)} \lambda K_i}{\kappa_i c(\lambda)}}. \quad (\mathbf{102})$$

We solve for $\lambda/c(\lambda)$ and insert into equation (**101**),

$$\phi_i(\lambda) = \phi_i^{\text{hi}} \frac{\lambda}{\lambda^{\max}} + \left(1 - \frac{\lambda}{\lambda^{\max}}\right) \frac{\xi_{s(i)} \sqrt{\frac{\gamma_{p(i)}\lambda^{\max} K_i}{\kappa_i}}}{\sum_i \xi_{s(i)} \sqrt{\frac{\gamma_{p(i)}\lambda^{\max} K_i}{\kappa_i}}}. \quad (\mathbf{103})$$

To obtain equation (**3**), we identify $\phi_i^{\text{hi}} \approx \frac{\gamma_i \lambda^{\max}}{\kappa_i}$, $c_i = \sqrt{\kappa_i K_i/(\gamma_i \lambda^{\max})}$ and $\bar{c} = \sum_i \phi_i^{\text{hi}} \xi_{s(i)} c_i$. In the limit of vanishing growth, resources are allocated to minimize metabolite concentrations in restricted pathways ($\xi > 0$). At higher growth rates, the same summation constraint yields an increase in the flux-balancing proteome fraction, and a concurrent decrease in the metabolite minimizing proteome fractions, recovering Eq (**28**). Proteome resource reallocation reflects the transition of the main cellular objective from metabolite concentration minimization to flux balance at high metabolite concentrations. In our detailed derivation, we find that this transition spans a wide range of growth rates in the physiological parameter regime.
Importantly, all metabolite minimizing proteome fractions depend in the same manner on growth rate; they are proportional to $\sqrt{\lambda/c(\lambda)}$, with an enzyme-specific proportionality constant. As a consequence of the summation constraint, the total metabolite minimizing proteome fraction (and therefore every single one) must decrease linearly with increasing growth rate. The non-linearity of enzyme kinetics (**9**) must cancel the non-linearity of the metabolome's growth response (**5**). Crucially this simplified argument shows that enzyme-specific linear growth laws also arise if the reason for metabolite concentration minimization differs from metabolite dilution, and if all metabolic reactions would follow a different kinetic law, such as quadratic kinetics, $\phi_i = \frac{j_i}{\kappa_i}(1 + \frac{K_i}{\rho_i^2})$.

As in the main text, we analyze the response factor $Q_i(\lambda) = \phi_i(\lambda)/\phi_i^{\text{hi}}$ of each individual enzyme as the product of intra-pathway ($i \in p$) and cross-pathway ($p \in P$) response factors,

$$Q_i = I(c_i, Q_p^v)\, Q_p(Q_1^v, \ldots, Q_P^v). \quad (\mathbf{104})$$

Consistent with sector models, we find that the decomposition can be expressed as $Q_p \sim v_p(\lambda^{\text{hi}})/v_p(\lambda)$ with a normalization across all pathways determining the proportionality constant,

$$Q_p(Q_1^v, \ldots, Q_P^v) = \frac{1}{Z_P} \frac{1}{Q_p^v}. \quad (\mathbf{105})$$

The normalization across all pathways $p \in P$,

$$Z_P = \sum_{p=1}^{P} \frac{\phi_p^{\text{hi}}}{Q_p^v}, \quad (\mathbf{106})$$

simplifies to $Z_P = \lambda^{\max}/\lambda$ by inserting the definition of Q_p^v. In conjunction, the intra-pathway response is given by the intra-pathway response

$$I(c_i, Q_p^v) = \left(\frac{c_i}{\bar{c}_p} + \left(1 - \frac{c_i}{\bar{c}_p}\right) Q_p^v\right). \quad (\mathbf{107})$$

Importantly, c is defined equivalently to c in equation (**3**), but modulated by a pathway-specific normalization $\bar{c}_p = \sum_{i \in p} (\phi_i^{\text{hi}} K_i \kappa_i/\gamma_{p(i)})/\phi_p^{\text{hi}}$ instead of a whole-cell dependent normalization. Thus, starting from the assumption of sector-specific, growth independent restriction factors ξ_s, we recover growth linear resource reallocation on the level of pathways - based on realtive changes in pathway efficiencies, on the level of individual enzymes relative to the total proteome, and within pathways relative to the pathway total when using Q_p^v instead of growth rate.

Application to more complex network topologies

We show in the main text that individual expression levels in a pathway remain linear in growth rate, as long as resource allocation to the rest of the metabolic network is linear in growth rate. We illustrate below that this requirement is satisfied in more complex metabolic networks. Specifically, we discuss three examples of metabolic pathways of different topologies.

Linear pathways as building blocks in more complex networks. Optimal resource allocation in a metabolic pathway of length ℓ_p which does not span the entire metabolic network must internally still minimize the total proteome mass at a given production flux J_{ℓ_p} (**62**). Therefore, the constitutive relations for cellular resource allocation (**71**) and (**72**) also apply to pathways embedded in more complex metabolic networks. If the pathway is part of a longer metabolic pathway that represents the entire metabolic network, linear allocation to the remainder of the network follows directly from the calculus above. Neglecting any bias introduced by unrestricted pathways, the aggregate expression change of a pathway p,

$$\frac{\sum_{i\in p}\phi_i(\lambda)}{\sum_{i\in p}\phi_i^{\mathrm{hi}}} \approx \bar{c}_p + (1-\bar{c}_p)\,\frac{\lambda}{\lambda^{\max}}. \tag{108}$$

is given by its mass-average response parameter

$$\bar{c}_p \equiv \frac{\sum_{i\in p}\phi_i^{\mathrm{hi}} c_i}{\sum_{i\in p}\phi_i^{\mathrm{hi}}}. \tag{109}$$

Individual pathways exhibit biased distributions of response parameters, for example, due to the particular biochemical reactions they catalyze. Such biases lead to systematic resource reallocation between pathways under nutrient limitation. To create pathways with a biased distribution of response parameters as shown in S3 Fig, a maximum entropy distribution of kinetic parameters in the pathway $P_p(K,\kappa)$ under a constraint on the expectation value of c can be obtained using a suitable Lagrange multiplier a as $P_p(K,\kappa) \sim P(K,\kappa)\exp(-ac(K,\kappa))$.

Branched networks. We focus on one particularly interesting use case that can be directly linked to existing proteomics studies: the consumption of two different, non-interchangeable environmental nutrients by the cell, such as a carbon and a nitrogen source. We specifically model this system as two convergent pathways with two different environmental nutrients (S3D–F Figs). To ease the notation, we call one pathway the N-branch and the other the C-branch. At the convergence point, both metabolite types (C-type and N-type derived from the two nutrients) are required for a single reaction. The product of this reaction is further converted by a third pathway, the stem of the metabolic network.
We assume that the enzyme at the convergence point exhibits Michaelis-Menten kinetics in both of its substrates – i.e., both substrates are needed for the reaction to proceed,

$$J_y = \frac{\kappa_y M_y}{1 + \frac{K^N}{\rho_N} + \frac{K^C}{\rho_C} + \frac{K^{CN}}{\rho_N \rho_C}}. \tag{110}$$

This rate law features a kinetic constant K^{CN} (with units of concentration squared), for which no physiological parameter regime is known. We here assume that $K^{CN} = K^N K^C$.

$$J = \frac{\kappa M}{(1+\frac{K^N}{\rho_N})(1+\frac{K^C}{\rho_C})}. \tag{111}$$

We further extend the framework of mass conservation to the convergence point: we assume that the two metabolite types are used with a fixed mass stoichiometry. The C-branch contributes a mass flux fraction γ_C to the downstream mass flux, the N-branch $\gamma_N = 1 - \gamma_C$, with $0 < \gamma_C < 1$.
Enzymes along the C-branch and the stem form a chain of ℓ enzymes. Its enzymes are numbered as before. On the other hand, enzymes in the N-branch are numbered by their position in the branch. To differentiate, we denote indices in the N-branch by a tilde $\tilde{\cdot}$. The N-branch has length $\tilde{\ell}$. Let y be the index of the enzyme with two substrates, so that $\rho_C \equiv \rho_y$. Mass conservation demands

$$J_C \equiv J_{y-1} = \gamma J_y + J_\ell \rho_C, \qquad \text{and} \qquad J_N \equiv J_{\tilde{\ell}} = (1-\gamma) J_y + J_\ell \rho_N. \tag{112}$$

We can now express M_y as a function of only fluxes and kinetic constants,

$$M_y = \frac{J_y}{\kappa_{CN}} \left(1 + \frac{K^N J_\ell}{J_N - (1-\gamma) J_y} + \frac{K^C J_\ell}{J_C - \gamma J_y} + \frac{K^{CN} (J_\ell)^2}{(J_N - (1-\gamma) J_y)(J_C - \gamma J_y)} \right). \tag{113}$$

All other enzyme masses are still given by Eq (**58**). Thus, all fluxes from $J_\ell^\star$ to $J_y^\star$ can still be constructed iteratively according to Eq (**65**) from a starting condition of the iteration $J_{\ell-1}^\star$ (and $J_\ell^\star$, which sets system size). Analogously, we can pick another starting condition $J_N^\star \equiv J_{\tilde{\ell}}^\star$ for the N-branch of the cell. This starting condition reflects an additional degree of freedom in space of growth-maximizing metabolic states solution: next to system size (associated with J_ℓ) and nutrient environment (associated with $J_{\ell-1}$), which were both also present in the linear chain, an optimal state is now also dependent on a second nutrient concentration in the environment (associated with J_N). For any J_N, one can solve $\partial M_P / \partial J_y = 0$ for J_C analytically by a suitable computer algebra system. For all other criteria on optimality, Eq (**113**) behaves like a regular Michaelis-Menten kinetic rate law with

$$\kappa_y \equiv \frac{\kappa_{CN}}{1 + \frac{K^N J_\ell}{J_N - (1-\gamma) J_y}}, \qquad \text{and} \qquad K_y \equiv \frac{\kappa_y}{\kappa_{CN}} (K^C + \frac{K^{CN} J_\ell}{J_N - (1-\gamma) J_y}). \tag{114}$$

Thus, the iterative construction of the optimal metabolic state can proceed until $J_0^\star$, where a matching environmental nutrient concentration must be determined. In comparison to the solution of a linear chain with ℓ enzymes, all fluxes $J_{i<y}$ are scaled by γ. Analogously, all fluxes in the N-branch can be constructed, once $J_C^\star$ is known, by viewing the N-branch and the stem together as a linear chain. In this manner, a consistent solution is always found, because the same equation $\partial M_P / \partial J_y = 0$ is used to determine first J_C from a given J_N and then J_N again from the determined J_C. Due to the irreversible nature of reactions considered, matching environmental nutrient concentrations ρ_0 and $\rho_{\tilde{0}}$ can be determined (even for constrained expression levels ϕ_0 and/or $\phi_{\tilde{0}}$), analogously to the linear chain. To parameterize the branched network, we assume that the substrate mass M_S on each branch is given by γ or $1-\gamma$ times the substrate mass present in the stem of the network, respectively. While M_E, ν, and the distributions of k_{cat} and $\tilde{K}$ are the same as in the stem, thus both K and κ are on average reduced by a factor of γ (or $1-\gamma$) in comparison to their values in the stem.
In the nutrient-rich regime, optimal resource allocation in two convergent pathways qualitatively resembles optimal resource allocation in a single chain. Scaled metabolite concentration gradients in each separate pathway are qualitatively unaffected by the altered topology. They exhibit a strong decrease over the first few reactions after nutrient uptake and a weaker decline after. At the convergence enzyme itself, the cost of metabolite production suddenly increases, requiring only one production pathway beforehand, but both in the shared stem. Thus, saturation drops at the convergence.

If one of the two nutrients is available at a growth-limiting concentration, the proteome reallocation within the branch with limited nutrient influx and in the stem resembles the proteome reallocation in a single chain: high-c enzymes are up-regulated while low-c enzymes are down-regulated (S3D and S3F Figs). All metabolites of sectors C and T are derived from a growth-limiting nutrient. At a lowered growth rate, their costs increase according to equation (**86**). Therefore, metabolite concentrations in sectors C and T are reduced under nutrient limitation. On the other hand, the N-sector does not use the growth-limiting nutrient, and metabolites $\tilde{\rho}_i$ are not required to fulfill eq. (**84**) with metabolites ρ_j from sectors C or T. While flux in the N-sector also changes approximately proportional to the growth rate, metabolite costs in the N-sector do not increase under C-limitation. Instead, costs remain dominated by proteome allocation to upstream reactions, as previously in the nutrient-rich environment. As a consequence, metabolite concentrations in the N-sector remain approximately constant, and proteome fractions are reduced proportionally with decreasing growth rate. For approximately constant, high ρ_N, equation (**110**) corresponds to a Michaelis-Menten reaction in ρ_C with slightly altered kinetic constants. Taken together, C-limitation leads to resource reallocation from the unaffected branch and low-c enzymes in the affected branch and stem to high-c enzymes in the affected branch and stem.

Non-linear pathways. Metabolic networks and pathways contain non-linear elements. A prominent example in central carbon metabolism is the citric acid cycle. In the citric acid cycle, oxaloacetate is produced in several metabolic steps from citrate, while the synthesis of citrate itself requires oxaloacetate – together with a second substrate derived from glucose via glycolysis. Intermediates of the citric acid cycle are used for amino acid synthesis. We represent this topology as a linear pathway (from glucose via intermediates of the citric acid cycle to protein biosynthesis) with an additional feedback cycle: a focal reaction y (representing citric acid synthesis) of the linear pathway uses a second substrate derived from a metabolite produced by reaction $y+k$ according to Michaelis-Menten-kinetics in both substrates (**110**). Under nutrient limitation, all metabolites of this cyclic network must fulfill equation (**84**). As a consequence, all metabolite costs increase equally, and metabolite concentrations throughout the pathway decrease. Due to the variable concentration of the second substrate ρ_N, equation (**110**) can no longer be written as a Michaelis-Menten reaction in ρ_C with constant kinetic parameters. Equations (**71**) and (**72**) must be adjusted for the focal reaction to reflect the altered kinetic rate law. Combining the optimality condition and metabolite costs (**70**a, b) with the kinetic rate law (**111**), we obtain

$$\rho_C^\star = \sqrt{\frac{K_C}{\kappa_{CN}}}\sqrt{\frac{j_{CN}^\star}{\beta_C^\star}\left(1+\frac{K_N}{\rho_N^\star}\right)}, \tag{115}$$

$$\phi_{CN}^\star = \beta_C^\star\rho_C^\star\left(1+\frac{\rho_C^\star}{K_C}\right). \tag{116}$$

The same equations are valid under the exchange of the indices C and N. In the limit of strong nutrient limitation ($\rho_N^\star \ll K_N,\ \rho_C^\star \ll K_C, \beta_C^\star \approx \beta_N^\star$), both equations of type (**115**) together yield

$$\left(\frac{\rho_C^\star}{K_C}\right)^3 \approx \frac{K_N}{K_C^2\kappa_{CN}}\frac{j_{CN}^\star}{\beta_C^\star} \tag{117}$$

Substrate concentrations of the focal reaction of the metabolic cycle scale as $(j/\beta)^{1/3}$, in contrast to a scaling as $(j/\beta)^{1/2}$ for all other reactions – presenting a relatively weaker decline with decreasing growth rate and increasing metabolite cost. Even though both

substrates are derived from the growth-limiting nutrient, the second-order rate constant K^{CN} at reaction y thus prohibits a linear reduction of both substrate concentrations with decreasing flux at low growth rates. We speculate that this effect results in largely growth-independent optimal co-factor concentrations, and could explain why neglecting common co-factors is allowable. At the same time, the reduction of two substrate concentrations results in a faster-than-proportional reduction of v_y with decreasing growth rate. As a consequence, the focal enzyme's expression increases in comparison to all other reactions (**116**). To compute optimal resource allocation in a cyclic network under variable degrees of nutrient limitation (S3G–I Figs), we numerically determine sets of fluxes, j, that fulfill all constitutive equations (**70**b-**72**, **115**, **116**), a constrained protein density (**51**) and mass conservation (**57**) for different values of β_0.

Model evaluation against physiological cell characteristics

Applicability to individual cascades. Our calculations indicate that the discovered flux- and saturation gradients, as well as linear expression level changes upon nutrient limitation, are not just properties of averages, but can be recovered for individual parameterizations of pathways. We illustrate this result in A4 Fig. Quantitatively, position and biochemical quality account for about 98 % of the variances in scaled metabolite concentrations and expression levels, respectively, in the nutrient-rich regime, as well as about 80 % of the corresponding variances under nutrient limitation in our ensemble of optimal resource allocation in physiologically parameterized cascades. These fractions denote percent reductions in squared residuals before and after applying the re-scaling derived through our analytical calculations (see A4 Fig). We thus expect our results to be applicable to a single physiological parameterization, as would be encountered in a given experiment. Beyond metabolic chains representing the full conversion of environmental nutrients to new protein biomass, our analysis of branched and cyclic metabolic units indicates that our predictions are also applicable to individual pathways embedded in more complex metabolic networks, if they are converting metabolites derived from a growth-limiting environmental nutrient.

Analyzed metabolic pathways. Microbial metabolic networks generally exhibit high topological complexity. Reactions often convert multiple metabolic precursors and contribute to multiple metabolic functions. In contrast, our model framework is best suited for pathways consisting of several successive reactions with similar flux in a given condition, and equal dependence on the availability of specific environmental nutrients. To identify metabolic pathways within the metabolic network, we used the KEGG database and retrieved all 217 annotated metabolic modules. Next, we matched the enzymes involved in each module with Uniprot IDs, and identified modules for which at least 5 proteins were detected in all growth conditions of a recent proteomic dataset for *E. coli* growth under carbon limitation. This yielded 55 metabolic modules. To remove pseudoreplicates, we chose among all modules with more than 50 % overlap with another module (e.g., Gluconeogenesis overlapping with Glycolysis) only those with the larger number of proteins included, resulting in a final count of 34 reliably detected metabolic modules. S1 Table lists these modules together with their detected constituent proteins.

Growth-linearity of expression data, To test whether the proteomics data can be explained by a linear model of expression as a function of growth rate, we test whether the deviations between a linear fit and the measured expression data can be explained by measurement errors.

We used the median coefficient of variation between biological replicates reported by Mori et al. of $\Delta\phi_i/\phi_i = 10.7\%$[14] as an estimate for measurement errors and verified that the root mean squared error between biological replicates is approximately proportional to a protein's expression level. We use this error model to compute the root mean squared error between a linear expression model $\phi_i^{\text{fit}}(\lambda) = \phi_i^{\text{hi}}(\hat{q}_i + (1-\hat{q}_i)\frac{\lambda}{\lambda^{\text{hi}}})$ and the measured data at various growth rates under carbon limitation.

Among all proteins detected by Mori et al.[14], 1647 were detected in all conditions of carbon limitation. Of those, 549 belong to any metabolic module in *E. coli*. We find that a vast majority of 516 metabolic proteins are explained by a linear model within a root mean squared error margin of three standard deviations. We matched detected proteins to KEGG modules by retrieving all KEGG IDs annotated for each metabolic module, converting them to Uniprot IDs[93] using the KEGG database and comparing to the Uniprot IDs reported by Mori et al.. Modules were analyzed further, if at least five proteins were matched to a metabolic module. Metabolic modules can overlap. If more than 50 % of matched proteins were also matched to a single other module, the module with fewer matched proteins was excluded from further analysis.

Predicted correlations in proteomics and metabolomics datasets. Our model predicts a correlation between protein growth responses and protein expression levels, as both depend on kinetic parameters. For physiologically small dilution strength, most enzymes are well-saturated, and the quadratic term dominates Eq (**72**),

$$\frac{\phi_i}{K_i} \approx \beta_i \left(\frac{\rho_i^\star}{K_i}\right)^2 \qquad \text{for } \left(\frac{\rho_0}{K_0} \gtrsim 1\right). \tag{118}$$

We insert Eq (**71**), (**97**) and $j_i \approx \lambda + \mathcal{O}(\epsilon)$, to find $\phi_i/K_i \sim 1/c_i^2$. Together with Eq (**98**), this defines the correlation

$$\log\left(\frac{\phi_i^{\text{lo}}}{\phi_i^{\text{hi}}}\right) \approx -\frac{1}{2}\log(\phi_i^{\text{hi}}) + \frac{1}{2}\log(K_i) + \text{const.} \tag{119}$$

We predict an anti-correlation with log-log-slope of $-1/2$ between expression levels in the nutrient-rich regime and relative expression levels in the limit of vanishing growth. We note that the affinity-dependent term $\log(K_i)/2$ may introduce considerable noise. If no affinities are known, averaging over a large number of enzymes may be required to identify the anti-correlation.

We here test whether an anti-correlation is present in the metabolic modules identified above. In a majority of metabolic modules, we observe an anti-correlation stronger than expected from regression to the mean. In particular, we test whether correlation patterns arise under exchange of ϕ_i^{lo} and ϕ_i^{hi}. Using the same approximations as above, we find $\phi_i^{\text{lo}} \sim \sqrt{K_i/\kappa_i}$ and $\phi_i^{\text{hi}} \sim 1/\kappa_i$. This results in an ambiguous predicted correlation pattern. If variances in κ and K are equal and independent, no correlation would be expected. We indeed find no clear anti-correlation of $\hat{q}_i^{-1}$ with ϕ_i^{lo} (S7 Fig).

Inclusion of metabolomic data. We matched the Enzyme Commission (EC) numbers reported by Bennett et al.[16] with catalyzing proteins by querying UniProt[93] for *E. coli* K12 (taxonomy_id:83333) proteins associated with these reaction identifiers. Retrieved protein identifiers were matched with expression data acquired by Mori et al.[14]. In this manner, 221 proteins were identified for which both expression data and metabolite concentrations (excluding ATP- and NAD-related cofactors) were previously measured. If more than one metabolite concentration or substrate affinity was assigned to a protein, we used the geometric mean of all assigned values. Subsequently, we analyzed all pathways for which five or more such proteins were identified, yielding eight

pathways[49]. Data resolved by pathway is shown in Fig 3. We compare equations (**71**) and (**97**) to derive the second correlation pattern

$$\log\left(\frac{\phi_i^{\rm lo}}{\phi_i^{\rm hi}}\right) \approx -\log(\frac{\rho_i^{\rm hi}}{K_i}) - \log(\beta_i) + \text{const.}, \tag{120}$$

which predicts an anti-correlation slope of $y = 1$. We find that variation in β lowers this slope on average to $y = 0.7$ (S4 Fig). To compute significance tests for both the predictive value of metabolomics data and protein expression levels in a nutrient-rich environment, datasets were corrected for pathway-specific biases by subtracting the pathway-average log protein growth response and log substrate saturation ρ/K or log expression $\phi^{\rm hi}$ from log data and subsequently aggregating over all eight pathways. We determine the significance level of a non-zero slope of the correlation between metabolite saturation or expression at high growth and protein growth responses under nutrient limitation (S6 Fig).

Our analysis of the proteome could further be improved by including additional kinetic or metabolomic data, or metabolite concentration data under nutrient limitation. Unfortunately, accurate absolute metabolomic and kinetic data are not available for many reactions. A small number of reactions were recently analyzed by Dourado et al.[46]. In this dataset, quantitative deviations from the original enzyme-substrate relationship appear too small for the considered enzymes for meaningful analysis of systematic shifts induced by variable metabolite costs β in nutrient-rich environments. We expect that ongoing efforts to expand the availability and accuracy of kinetic data[37] will allow for a direct test of our predictions on metabolite concentrations in nutrient-rich environments. Under nutrient limitation, we predict that the added cost of metabolite production shifts the optimal enzyme-substrate relationship to lowered metabolite concentrations. Interestingly, a qualitatively similar deviation from the unshifted enzyme-substrate relation was observed experimentally when reducing the growth rate by switching the carbon source of a growth medium[46].

Regulatory constraints

Regulation of the uptake enzyme. In a simple regulatory model, a metabolite can directly influence protein expression. Here we assume that such a regulatory framework leads to a Hill function governing an enzyme's expression level as a function of its substrate's concentration, with a basal expression level ϕ^0 and an expression level ϕ^∞ at full promoter saturation with the metabolite. At a concentration $K^{\rm reg}$, the promoter is half-saturated,

$$\bar{\phi}_i(\rho_i) = \phi^0 + \frac{a_i}{1 + K_i^{\rm reg}/\rho_i}, \qquad \text{with } a_i = \phi_i^\infty - \phi_i^0. \tag{121}$$

In this model, the metabolite can either induce protein production ($\phi^0 < \phi^\infty$) or inhibit protein production ($\phi^0 > \phi^\infty$). We focus first on the nutrient uptake reaction ($i = 0$). Consider a cell that efficiently converts environmental nutrients into new protein biomass ($J_0 \approx J_\ell(1 + \mathcal{O}(\epsilon))$) and that exhibits a small uptake protein mass fraction $\phi_0 \ll 1$ at high nutrient supply. The flux that can be efficiently handled by downstream metabolism ($i = 1, \ \ldots, \ \ell$) is proportional to $1 - \phi_0$. For sufficiently small ϕ_0 and only moderate reductions in nutrient availability, keeping the nutrient uptake rate approximately constant therefore recapitulates the unconstrained optimal resource allocation strategy,

$$\phi_0^\star(\rho_0) \approx (1 + \frac{K_0}{\rho_0})\phi_0^\infty, \qquad \text{with} \qquad \phi_0^\infty \equiv \lim_{\rho_0\to\infty} \phi_0^\star \qquad \text{for } \left(\frac{\rho_0}{K_0} > 1\right). \tag{122}$$

In the case of the nutrient uptake reaction, we thus expect that the proteome fraction of the uptake enzyme should increase with decreasing nutrient availability (A3A Fig). For the reasons outlined in the main text, we assume that the approximate form (**122**) is only valid at high growth rates. Instead, expression levels off to $\phi^0 = \phi_0^{\max}$ well before the uptake protein becomes the dominant proteome mass fraction in the cell. To achieve qualitatively comparable up-regulation of all parameterizations in the ensemble despite different optimal expression levels at high nutrient supply, we use $\phi^0 = \phi_0^{\max} \equiv n\phi_0^{\infty}/(1 + n\phi_0^{\infty})$, where n determines the relative expression level of ϕ_0 under nutrient limitation. In this manner, ϕ_0 can be up-regulated n-fold for parameterizations with small ϕ^{∞} but does not exceed 1 for larger ϕ^{∞}. In all simulations, we use $n = 5$. To mimic optimal resource allocation (**122**), K^{reg} must then be $K^{\text{reg}} = K_0\phi_0^{\infty}/\phi_0^{\max}$. This regulatory scheme approximates unconstrained optimal resource allocation to the uptake protein at high nutrient concentrations $\rho_0/K_0 \gg 1$, and smoothly interpolates to a cellular state with a constrained nutrient uptake flux at lower nutrient concentrations (A3 Fig).

Approximating optimal resource allocation by feed-forward regulation. Can a metabolic system come close to an optimal growth state by local regulation of its enzymes[69]? Mimicking optimal resource allocation under variable degrees of nutrient limitation requires the stable convergence of metabolite and enzyme concentrations from a perturbed state to a close-to-optimal stationary state[69], and growth-dependent shifts in metabolite and enzyme levels, $\rho_i^\star(\lambda)$ and $\phi_i^\star(\lambda)$, as given by equations (**93**, **94**). We define an extended regulatory scheme for system-wide metabolism. Protein concentrations change by synthesis with rate b_i and by dilution, $\dot{\phi}_i = b_i - \lambda\phi_i$. In the simplest case of transcriptional regulation, the stationary level

$$\bar{\phi}_i = \frac{b_i}{\lambda} = \phi_i^0 + \frac{a_i}{1 + K_i^{\text{reg}}/\rho}, \qquad (\mathbf{123})$$

depends on the equilibrium binding of a transcription factor present at density ρ (Fig 5A). This regulation function has three parameters: the basal level ϕ_i^0, the regulatory amplitude a_i, and the regulatory binding constant K_i^{reg}.
We consider two simple regulatory motifs[94]: feed-forward and feedback regulation[95]. Either the substrate, ρ_i, of each metabolic step, or its product, ρ_{i+1}, controls the transcription of its cognate enzyme ϕ_i (Fig 5A). First, we focus on a feed-forward scheme, in which each metabolite regulates the expression level of its consuming enzyme (Fig 5A, top). Transcription takes place with enzyme-specific rates

$$b_i^t(\rho) = b_0^t\left(\phi^0 + \frac{a_i}{1 + K_i^{\text{reg}}/\rho_i}\right), \qquad (i = 2, \ \ldots, \ \ell), \qquad (\mathbf{124})$$

where b_0^t is a basal transcription rate. We use metabolite-mediated regulation specified by the set of parameters $\mathcal{R}_m(\kappa, K) = (\phi_0, a, K^{\text{reg}})(\kappa, K)$ with

$$\phi_i^0 = \frac{\frac{K_i}{\kappa_i}}{\sqrt{\frac{K_i}{\kappa_i}}\sum_{i=2}^{\ell}\sqrt{\frac{K_i}{\kappa_i}}}, \qquad a_i = \frac{\frac{1}{\kappa_i}}{\sum_{i=2}^{\ell}\frac{1}{\kappa_i}} - \phi_i^0, \qquad K_i^{\text{reg}} = \frac{\sqrt{\frac{K_i}{\kappa_i}}\sum_{i=2}^{\ell}\sqrt{\frac{K_i}{\kappa_i}}}{\sum_{i=2}^{\ell}\frac{1}{\kappa_i}}. \quad (\mathbf{125})$$

As a second step, we use a minimal model of translation that specifies protein synthesis rates proportional to the corresponding transcription rates,

$$b_i(\rho, \lambda) = \frac{\lambda\, b_i^t(\rho)}{\sum_{k=1}^{\ell} b_k^t(\rho)}. \qquad (\mathbf{126})$$

This form sets the overall normalization of protein synthesis, $\sum_k b_i(\rho, \lambda) = \lambda$, as required in a steady state with dilution at rate λ, and leads to stationary expression levels $\bar{\phi}_i(\rho)$

given by equation (**123**). The relation between transcription and translation rates reflects competition for a growth-dependent global resource, e.g., RNA polymerases, translating ribosomes, or energy.

First, we verify the consistency relation

$$\bar{\phi}_i(\rho_i^\star(\lambda), \mathcal{R}_m) \approx \phi_i^\star(\lambda) \qquad (i = 2, \; \dots, \; \ell), \tag{127}$$

where $\rho_i^\star(\lambda)$ and $\phi_i^\star(\lambda)$ are given by equations (**29** – **28**). As required for an optimal growth response to nutrient limitation, rate-optimized enzymes ($c_i/\bar{c} > 1$) must exhibit a higher expression level at low metabolite concentrations. Consequently, rate-optimized enzymes are repressed, and specificity-optimized enzymes ($c_i/\bar{c} < 1$) are activated by their cognate metabolite.

Second, we numerically study time-dependent metabolic states $\mathcal{S}(t) = (\rho(t), \phi(t))$ given by the dynamical equations

$$\frac{d\rho_i(t)}{dt} = \frac{\kappa_{i-1}\phi_{i-1}(t)}{1 + \frac{K_{i-1}}{\rho_{i-1}(t)}} - \frac{\kappa_i \phi_i(t)}{1 + \frac{K_i}{\rho_i(t)}} - \lambda(t)\rho_i(t), \qquad (i = 2, \; \dots, \; \ell), \tag{128}$$

$$\frac{d\phi_i(t)}{dt} = b_i(\rho_i(t), \lambda(t)) - \lambda(t)\phi_i(t) \qquad (i = 1, \; \dots, \; \ell) \tag{129}$$

with $\lambda(t) = \kappa_\ell \phi_\ell(t)/(1 + K_\ell/\rho_\ell(t))$ and a time-dependent input signal $\rho_0(t)$ (Fig 5).

The adaptive dynamics of the metabolic state, $\mathcal{S}(t) = (\rho, \phi)(t)$, consists of regulated changes of protein levels and correlated changes of metabolite levels given by mass conservation, equation (**13**). Interestingly, when we use metabolite-mediated feed-forward regulation specified by the set of parameters $\mathcal{R}_m = (\phi^0, a, K^{\text{reg}})$ for the entire network, metabolite and enzyme concentrations robustly converge to a defined stationary state, independent of the simulation's starting conditions. Fig 5B shows these dynamics under time-dependent nutrient limitation, $\rho_0(t)$, switching between subsequent stationary levels. Rate-optimized and specificity-optimized enzymes change in opposite directions and settle to a stable stationary state $\bar{\mathcal{S}}(\rho_0)$ with growth rate $\bar{\lambda}(\rho_0)$ that matches the growth-optimal state in very good approximation. We find stable stationary states $\bar{\mathcal{S}}(\rho_0, \mathcal{R}_m)$ with

$$\begin{aligned} \bar{\rho}_i(\rho_0, \mathcal{R}_m) &= \rho_i^\star(\rho_0)(1 \pm 0.5), \\ \bar{\phi}_i(\rho_0, \mathcal{R}_m) &= \phi_i^\star(\rho_0)(1 \pm 0.05), \\ \bar{\lambda}(\rho_0, \mathcal{R}_m) &= \lambda^\star(\rho_0)(0.98 \pm 0.02). \end{aligned} \tag{130}$$

We note that no flux-sensing or growth-sensing mechanism is required to robustly implement growth-dependent gene expression. With regulatory parameters, which depend only on local reaction kinetics, $\phi_i^0 = s_i$, $a_i = r_i - s_i = r_i(1 - c_i)$, and $K_i^{\text{reg}} = s_i \epsilon^2$, the stationary expression level (**123**) follows the optimal growth response, $\bar{\phi}_i(\rho_i^\star(\lambda)) = \phi_i^\star(\lambda)$, up to terms of order ϵ (Fig 5C).

Metabolic response by regulation. Similarly, feed-back regulation can maintain a close-to-optimal metabolic state with the same regulatory parameters but $K_i^{\text{reg}} = s_{i+1}\epsilon^2$. In this case, the stability of metabolic states is more involved and can be achieved, for example, by a parameter bias towards inhibition combined with a post-transcriptional mechanism ensuring normalized total gene expression (S8 Fig).

More broadly, our analysis suggests that metabolite-induced regulation of enzymes can be robust to perturbations in metabolite and enzyme concentration and can generate partial optimization of metabolic states under diverse conditions. We note that the local regulatory schemes introduced above are not a unique solution to this optimization problem. Alternative schemes may employ both feed-forward and feedback regulation for

different reactions; the regulation of central metabolism also includes post-transcriptional mechanisms [96]. In metabolic networks with many constituents, local regulation of each enzyme is not a plausible scenario. However, few, broad-acting regulatory inputs can generate coarse-grained optimization of the average expression for co-regulated target genes in pathways or functional sectors [97].

Approximating optimal resource allocation by feedback regulation. Analogously, a feed-back regulation scheme (Fig 5A, bottom),

$$b_i^t(\rho) = b_0^t\left(\phi^0 + \frac{a_i}{1+K_i^{\mathrm{reg}}/\rho_{i+1}}\right), \qquad (i=2,\ \ldots,\ \ell-1), \tag{131}$$

can be implemented using the same set of regulatory parameters (**125**) as the feed-forward scheme presented above, except for an index-shift from i to $i+1$ in the definition of K_i^{reg}. This regulatory scheme cannot be applied to the final reaction of the network for lack of a product metabolite. We instead use the feed-forward scheme introduced above to control the expression of the final enzyme. As derived above, a close-to-optimal state is a fixpoint of this regulatory scheme. However, concentrations do not generally converge to this fixpoint from arbitrary starting conditions. A perturbation of an enzyme below its growth-optimal concentration leads to a lowered production of the following metabolite, and thus causes its concentration to drop. For specificity-optimized enzymes, such a drop in the product concentration would lead to down-regulation of their expression. Sufficiently strong down-regulation can lead to a vicious cycle and divergence from the close-to-optimal steady state.
A stable feedback regulatory scheme can be implemented if the additional regulatory degree of freedom associated with expression normalization by translation is used. If all transcription rates are modulated by the same growth-dependent factor, the stationary state is unaltered. Due to this degeneracy, the combination

$$\phi_i^0 = \theta\frac{\frac{K_i}{\kappa_i}}{\sqrt{\frac{K_i}{\kappa_i}}\sum_{i=2}^{\ell}\sqrt{\frac{K_i}{\kappa_i}}}, \qquad a_i = \frac{\frac{\theta}{\kappa_i}}{(1+\theta)\sum_{i=2}^{\ell}\frac{1}{\kappa_i}} - \phi_i^0, \qquad K_i^{\mathrm{reg}} = \frac{\sqrt{\frac{K_{i+1}}{\kappa_{i+1}}}\sum_{i=2}^{\ell}\sqrt{\frac{K_i}{\kappa_i}}}{(1+\theta)\sum_{i=2}^{\ell}\frac{1}{\kappa_i}}. \tag{132}$$

yields the same stationary state. We find numerically that this combination is additionally stable against perturbations if a sufficiently strong bias towards feedback inhibition, $\theta \gg 1$, is used (S8 Fig).
Further regulatory degrees of freedom exist: Combinations of feed-forward and feedback schemes, using one metabolite to control multiple expression levels and using differently globally growth-modulated expression rates $b_i^t(\rho,\lambda) = b_i^t(\rho)b(\lambda)$ can all maintain a close-to-optimal state, but it remains to be explored under which conditions such regulatory schemes also converge to a close-to-optimal stationary state from arbitrary starting conditions.

Network evolution

Consider an enzyme mutation changing the affinity to its cognate metabolite, $\log K_i \to \log K_i + \delta\log K_i$, and/or the enzyme's catalytic constant, $\log\kappa_i \to \log\kappa_i + \delta\log\kappa_i$. We estimate selection on enzyme-metabolite binding and transition state energies in networks with a regulated physiological response specified by a regulation function depending on the reaction parameters, $\mathcal{R}(\kappa, K)$. We evaluate the selection coefficients, or relative growth effects in balanced growth, of amino acid changes that affect the metabolite

binding affinity, $K_i \to K_i + \Delta K_i$ $(i \neq 0)$,

$$\begin{aligned} \varsigma_i(\rho_0, \mathcal{R}, \kappa, K) &\equiv \frac{\Delta\bar{\lambda}(\rho_0, \mathcal{R}, \kappa, K)}{\bar{\lambda}(\rho_0, \mathcal{R}, \kappa, K)} \\ &= \frac{K_i}{\bar{\lambda}(\rho_0, \mathcal{R}, \kappa, K)} \frac{\partial\bar{\lambda}(\rho_0, \mathcal{R}, \kappa, K)}{\partial K_i} \Delta\Delta G_{K_i}, \end{aligned} \qquad \mathbf{(133)}$$

where $\Delta\Delta G_{K_i} = \Delta K_i / K_i$ is the corresponding change in reduced free energy of binding. In transition state theory, such a change, which only affects the Michaelis constant K_i at constant catalytic constant κ_i, corresponds to a mutation that equally alters the free energy of the substrate-bound enzyme and the high-energy transition state along the reaction coordinate towards product release.[98] The mutation selectively affects reaction rates at low substrate concentrations. We assume uniform effects $|\Delta\Delta G_{K_i}| = 1$, in line with typical amplitudes of mutations affecting protein evolution. In equation (**133**), we compute only the direct effect of the mutation on binding, while keeping the regulation function $\mathcal{R}(\kappa, K)$ at the wild-type parameters. We assume that regulation tunes the wild-type metabolic state $\bar{\mathcal{S}}(\rho_0, \mathcal{R}, \kappa, K)$ to near-optimal growth,

$$\bar{\lambda}(\rho_0, \mathcal{R}, \kappa, K) \approx \lambda^\star(\rho_0, \kappa, K); \qquad \mathbf{(134)}$$

An example of such regulation functions is the metabolite-mediated scheme $\mathcal{R}_m$ discussed above. This assumption is self-consistent if the evolution of protein binding affinities is followed by compensatory evolution of regulation, $\mathcal{R}(\kappa, K) \to \mathcal{R}(\kappa, K + \Delta K)$. However, details of the regulation function and its evolution are not relevant for our current purpose, to obtain an order-of-magnitude estimate of selection on protein binding affinities.

We compute $\varsigma_i(\rho_0, \mathcal{R}, \kappa, K)$ from the variation of optimal growth rates. This approximation couples local enzyme changes $K_i \to K_i + \Delta K_i$ and compensatory changes of regulation, $\mathcal{R}(\kappa_i, K_i) \to \mathcal{R}(\kappa, K_i + \Delta K_i)$, assuming a hypothetical simultaneous evolution of an enzyme's protein and cis-regulatory sequence, which slightly overestimates the mutant fitness. On the approximate fitness landscape defined by

$$\lambda^\star \approx \frac{j_0}{1 + \sum_{i=1}^{\ell} \rho_i(\lambda)} \qquad \mathbf{(135)}$$

with $\rho_i(\lambda)$ given by equation (**93**), we obtain selection coefficients

$$\begin{aligned} \varsigma_i(\rho_0, \kappa, K) &= -\frac{K_i}{\lambda^\star(\rho_0, \kappa, K)} \frac{\partial\lambda^\star(\rho_0, \kappa, K)}{\partial K_i} && \mathbf{(136)} \\ &\approx K_i \frac{\frac{1}{2}\sqrt{\frac{j_i}{K_i \kappa_i \beta_i}}}{1 + \sum_{k=2}^{\ell} \sqrt{\frac{K_k j_k}{\kappa_k \beta_k}}} && \mathbf{(137)} \\ &= \frac{1}{2}\rho_i(\rho_0, \kappa, K)\,[1 + O(\epsilon)]. && \mathbf{(138)} \end{aligned}$$

Here we have used that j_0 is independent of K_i (which is justified under nutrient limitation, see equation (**121**), and correct to order ϵ otherwise), and neglected position-dependent inhomogeneities and changes of the cost factors (i.e., $\beta_i \approx \beta_0 \approx$ const.), as appropriate under nutrient limitation. Using the growth response of ρ_i, we find

$$\varsigma_i \sim \rho_i^\star \sim \frac{\epsilon^2 \lambda}{(\lambda^{\max} - \lambda)} s_i, \qquad \mathbf{(139)}$$

with a regulation-dependent prefactor of order $1/\ell$. In the same rationale, variation of the growth-optimal metabolic state $\mathcal{S}^\star(\rho_0, \kappa, K)$ provides estimates of the buffering in

regulated networks,

$$\frac{\Delta\rho_i}{\rho_i} \approx \frac{1}{2}\frac{\Delta K_i}{K_i}, \tag{140}$$

$$\frac{\Delta\phi_i}{\phi_i} \approx \frac{1}{2}\left(1 - \frac{\lambda}{\lambda^{\max}}\frac{\phi_i^{\max}}{\phi_i}\right)\frac{\Delta K_i}{K_i}, \tag{141}$$

with $\phi_i^{\max} = \lambda^{\max}/\kappa_i$, which are obtained from equations (**29**) and (**28**).
We can compare this strength of selection with the case of a single enzyme at constant metabolite density,

$$\begin{aligned}\varsigma_0(\rho,\kappa,K) &= \frac{K}{j(\rho,\kappa,K)}\frac{\partial j(\rho,\kappa,K)}{\partial K} \\ &= \frac{1}{1+\rho/K}. \end{aligned} \tag{142}$$

This shows that in a network, moderate affinity changes of bulk enzymes are largely buffered by compensatory changes of the cognate metabolite, $\Delta\rho_i/\rho_i \approx -\delta K_i/K_i$, leaving a residual effect on growth by metabolite dilution.

Analogously, the selection coefficient $\tilde{\varsigma}_i$ associated with a change in catalytic constants, $\kappa_i \to \kappa_i + \Delta\kappa_i$ $(i \neq 0)$ can be estimated. We here focus on the change in optimal growth rate as an upper limit to selection coefficients. We assume that an amino acid change affects free energy of the transition state, ΔG^{++}, of the catalyzed process and thereby also affects catalytic constants exponentially.

We compute the selection coefficient associated with variation of κ at constant K/κ,

$$\tilde{\varsigma}_i(\rho_0,\kappa,K) = -\frac{\kappa_i}{\lambda^\star(\rho_0,\kappa,K)}\frac{\partial\lambda^\star(\rho_0,\kappa,K)}{\partial\kappa_i}\bigg|_{K_i/\kappa_i} \tag{143}$$

$$\approx \frac{1}{2}\frac{\epsilon^2\lambda^3}{\lambda^{\max}(\lambda^{\max}-\lambda)^2}r_i. \tag{144}$$

Such mutations selectively affect reaction rates at high substrate concentrations.
Importantly, selection coefficients are drastically smaller for enzymes embedded in a metabolic network than would be estimated from Michaelis-Menten kinetics alone. In a mean-field approximation, we find $\varsigma_i, \tilde{\varsigma}_i \sim \epsilon^2/\ell \sim 10^{-4}$ at physiological network parameters and intermediate growth rates. These buffered selection coefficients are at least two orders of magnitude lower than the growth effect of an affinity change for a single Michaelis-Menten pair at constant ρ, as given by equation.
Selection coefficients depend on the nutrient environment, which sets optimal metabolite concentrations and growth rate. At high growth rate, selection on specificities is of order ρ_i, whereas selection on catalytic constants is on the order of ϕ_i. For physiological kinetic parameters, this results in stricter selection of catalytic capacities at high growth for most enzymes. Under nutrient limitation, selection on specificities declines more slowly ($\sim \lambda$) than selection on catalytic constants ($\sim \lambda^3$). At intermediate growth rates, selection on catalytic constants and specificities is comparable and of order ϵ^2. At intermediate and low growth rates, the dominant objective of selection switches to selection on specificities.

Numerical simulations

Numerical computations were performed using Python. Equations (**65**), (**13**) and (**9**) were used to construct optimal balanced growth states of stochastically parameterized metabolic models from iteration starting conditions $(J_{\ell-1}, J_\ell)$. Iteration starting conditions were varied numerically using the Broyden-Fletcher-Goldfarb-Shanno algorithm to

match a nutrient-rich environment (Figs 2, S2–S3 and A2–A4), or match an otherwise defined environmental nutrient concentration (Fig S2, orange points). Subsequently, the ratio $J_{\ell-1}/J_\ell$ was reduced to construct optimal balanced growth states under nutrient limitation. In case of the branched metabolic network, also the metabolite concentration ρ_N was varied numerically for each ratio $J_{\ell-1}/J_\ell$ using the same algorithm to ensure a constant environmental nutrient concentration ρ_0^N under nutrient limitation of the C branch. Time evolutions of the feed-forward regulated metabolic cascades (Fig 5, Eqs (**128**) and (**129**)) were computed by means of Euler's method.

Cytoplasmic density affects optimal resource allocation

Throughout the whole paper, we assume that the protein mass density of the cell is approximately $400\,\mathrm{g\,L^{-1}}$, independent of environmental conditions. This assumption is in line with experimental observations [1,13,52–54], which indicate an approximately constant cellular dry mass density under nutrient limitation. However, literature estimates of the cellular protein mass density vary, and we can not exclude that the composition of the cell – in particular the fraction cellular dry mass which is protein – varies under nutrient limitation which may lead to a modified biosynthetic protein density at constant dry mass density, or exclude that cellular dry mass density changes within the margins of experimental accuracy. A recent study has proposed potential underlying mechanisms to a constant intracellular protein density based on a model of cellular self-replication similar to the one we present here [99]. Simultaneous optimization of protein density and proteome allocation under a nutrient limitation may be feasible but lies beyond the scope of this paper.

Generally, we predict that cells exhibit higher optimal metabolite mass fractions and lowered mean enzyme saturation at lowered protein mass density in the nutrient-rich regime. The metabolite mass fraction changes in proportion to the square-root fold change in protein mass density. The same behavior is expected under a nutrient uptake constraint, which concerns ϕ_0. Thus, changes in resource allocation induced by changes in protein mass density are likely negligible in comparison to the predicted changes due to differences in biochemical quality c at constant protein mass density. Only a constraint on j_0 (but unconstrained ϕ_0 relative to all other metabolic proteins in the cell) qualitatively yields a different behavior. In this case, both the cellular metabolite mass fraction and mean enzyme saturation would increase in the nutrient-poor regime with decreasing biosynthetic protein mass density.

Overview of modeling approaches to microbial resource allocation

A quantitative understanding of microbial resource allocation has developed along three closely related lines of research - constraint-based modeling, analytical investigation and numerical simulation - combining insights from enzyme kinetics, cell physiology, and systems-level modeling.

Early analytical work laid the conceptual foundation by linking enzyme activity, metabolite concentrations, and pathway fluxes in metabolic networks. Classical studies of enzymatic reaction schemes already derived steady-state relationships between intermediates and reaction rates in simple systems [39]. These ideas were later embedded into the framework of metabolic control analysis, which derived conditions on enzyme allocation that maximize flux under constraints [41,42]. Closely related contributions by Kacser and colleagues established how control of flux is distributed across enzymes and depends on enzyme saturation and system parameters [27], while work by Ehrenberg and Kurland highlighted the role of biosynthetic costs and growth optimization in shaping enzyme expression and cellular composition [40]. Together, these studies provided a transparent and mechanistic basis for understanding pathway efficiency and resource allocation

principles. However, they remained largely focused on mathematical properties of such systems, without explicitly addressing how resources are allocated in a concrete context, matching predictions with experimental data.

Relation to Flux Balance Analysis, ME-models and kinetic simulations

By assuming that the dominant constraint on metabolic fluxes is mass balance - specifically, the balance between production and consumption of all intracellular metabolites - constraint-based modelling reduces the problem to one of feasible flux distributions under stoichiometric constraints[8,9,100]. This abstraction enables efficient prediction of flux states in genome-scale networks without requiring detailed kinetic parameters. The key strength of flux-balance models lies in their scalability and predictive success across diverse environments, enabling the identification of active pathways and essential reactions.

At the same time, by construction, fluxes are largely decoupled from the underlying allocation of cellular resources to proteins and metabolites. As a consequence, while flux-balance-analysis captures what flux states are possible or optimal, linking these fluxes back to enzyme expression, metabolite levels, and biochemical constraints - and understanding how these quantities co-vary under environmental perturbations - remains challenging. Addressing this limitation has motivated complementary approaches that explicitly incorporate enzyme kinetics and resource allocation into metabolic modeling.

An important extension in this direction are ME-models (metabolism and expression models), which augment constraint-based frameworks by explicitly accounting for the biosynthetic cost of proteins and the coupling between metabolic fluxes and gene expression[22–25]. These models integrate kinetics and protein-production processes with metabolic networks, thereby enabling predictions of proteome allocation and growth rate under varying environmental conditions. In parallel, enzyme cost minimization provides a numerical framework for comparing the proteome costs of different kinetic realizations of the same fluxes and selecting resource allocation patterns that optimize growth[48].

These approaches demonstrated that intracellular metabolite concentrations and enzyme efficiencies are tightly coupled and that optimality principles can be solved numerically by first determining metabolite concentrations. Subsequent work extended this perspective to systems-level questions such as overflow metabolism and global proteome allocation strategies[71], and more recent efforts integrate flux optimization directly into kinetic frameworks[32]. Collectively, ME-models and fully kinetic simulations can, in principle, predict resource allocation and growth responses across environments by jointly optimizing fluxes, enzyme levels, and metabolite concentrations.

Despite these advances, numerical models typically rely on large parameter sets and optimization procedures, which can obscure the mechanistic origin of predicted patterns and limit interpretability. In particular, while features such as linear growth responses or coordinated proteome changes are often observed, it is unclear whether these are generic or parameter-specific. Moreover, both ME-models and kinetic models frequently predict flux-proportional resource allocation with approximately constant metabolite concentrations across conditions[27,43,47,71]. This prediction is closely related to earlier results from metabolic control theory and flux-balance approaches, which imply a “Universal Method” of flux-proportional enzyme allocation[27,43,101].

Experimental data, however, reveal systematic deviations from these predictions. Individual protein concentrations within the same metabolic pathway often change differently under nutrient limitation[12,14,64]. Our model explains these deviations by explicitly accounting for metabolite dilution. Under nutrient limitation, the cost of metabolite loss increases, leading to reduced optimal metabolite concentrations. These

changes in metabolite levels, in turn, alter optimal enzyme allocation in a pathway-specific and enzyme-specific manner.

This perspective is consistent with earlier resource allocation models under nutrient limitation[22], which proposed that cells minimize normalized fluxes per unit enzyme, v_i/κ_i, rather than total proteome mass. Our model provides a mechanistic basis for this principle: it arises naturally from minimizing metabolite dilution under constraints on protein mass and nutrient uptake. At the same time, we predict that reductions are not uniform; they depend on each enzyme's degree of saturation at high growth. In this way, our framework reconciles kinetic and constraint-based approaches while extending them to capture heterogeneous, enzyme-specific growth responses.

Relation to sector models

In parallel, empirical and coarse-grained approaches have identified robust patterns in proteome allocation. Proteome sector models show that large groups of functionally related proteins exhibit coherent and often linear scaling with growth rate across diverse conditions[10,14,21]. These models rationalize global resource reallocation at the level of large proteome sectors and have been successfully integrated with flux-based approaches[50]. However, they rely on the assumption of approximately constant enzyme efficiencies within sectors and do not explicitly account for metabolite concentrations and kinetic effects. As a result, they do not resolve heterogeneous responses of individual enzymes within pathways.

Conceptual syntheses of kinetic and sector-based modeling[47,57] emphasize that common allocation patterns can emerge from diverse underlying mechanisms, but still predict largely uniform responses within sectors. In contrast, experimental data reveal enzyme-specific linear up- and down-regulation even within the same pathway[12,14,64].

Our model provides a mechanistic link between enzyme-level and sector-level behavior. We predict and observe linear growth responses at the level of individual enzymes. As a result, proteome sectors, which aggregate many such responses, also exhibit approximately linear scaling, consistent with sector models. However, in our framework, these sector-level patterns emerge from underlying heterogeneity rather than uniform behavior.

Furthermore, our model suggests an alternative explanation for coordinated sector shifts: correlations between kinetic parameters and enzyme function[51]. For example, anabolic enzymes may be preferentially down-regulated under nutrient limitation not due to fixed saturation, but due to characteristic kinetic parameters such as lower K and κ values. In branched networks, we also recover partial correspondence between enzyme function and growth response: pathways unaffected by a limitation are down-regulated due to reduced flux demand at nearly constant metabolite concentrations, while pathways directly limited exhibit stronger and more heterogeneous responses. Thus, inter-sector reallocation may arise from a combination of kinetic parameter correlations, pathway-specific nutrient limitation, and changes in flux demand.

Relation to growth balance analysis

A distinct analytical line of research has developed general theories of optimal growth in metabolic networks with nonlinear kinetics. Dourado et al.[44,45] formulated a framework for balanced growth that incorporates arbitrary kinetic rate laws, metabolite dilution, and network topology. These studies derive general balance conditions and characterize the structure of optimal states, providing deep insight into how enzyme efficiencies, metabolite levels, and fluxes are interrelated. Their emphasis lies on identifying universal properties and local optimality conditions rather than computing explicit solutions, although such computations are in principle possible.

Importantly, these frameworks can yield predictive relationships independent of specific parameterizations, such as the substrate-enzyme scaling relation[46]. However, deriving concise, experimentally testable predictions typically requires further simplification. In contrast, our pathway-level model starts from a reduced setting that directly yields closed-form predictions for enzyme-specific responses. In an important distinction from Growth Balance Analysis, we do not impose a fixed total mass constraint on metabolites and proteins. Instead, we derive an effective constraint with a position- and environment-dependent weight β, arising from the proteome cost of compensating metabolite dilution. We additionally assume that reduced metabolite concentrations cannot be offset by unbounded overexpression of uptake proteins, introducing a physiologically motivated limitation absent in previous formulations.

Overarching comparison to established models

Taken together, existing literature has established that (i) enzyme kinetics and metabolite concentrations fundamentally constrain resource allocation, (ii) analytical modeling can describe kinetics-based allocation and capture its mathematical complexity, and (iii) global proteome allocation follows simple empirical laws. What remains less explored is an analytically tractable framework that connects these elements and yields quantitative predictions for enzyme-specific responses.

Our work addresses this gap by combining elements of these traditions into a simplified yet mechanistically grounded framework. Starting from balance relations closely related to enzyme cost minimization and balanced growth theory[44,45,48], we derive an analytically solvable model for a heterogeneous Michaelis-Menten pathway that explicitly includes metabolite dilution and nutrient-dependent constraints. This yields closed-form predictions for fluxes, enzyme abundances, and metabolite concentrations in terms of a small number of interpretable parameters, and in particular predicts how individual abundances change under nutrient limitation as a function of few reaction parameters.

Key to our analysis is a direct, statistical comparison of these predicted response patterns with experimental data. Such a statistical analysis was only possible, because our model decouples the response of individual enzymes from the global parameterization of the cell. In this manner, neither successful numerical nor analytical prediction of optimal resource allocation alone would have sufficed to conduct a comparable statistical validation.

Changes in temperature

Catalytic constants and substrate affinities change with temperature. A related kinetic model incorporating metabolite dilution has recently been used to explain growth responses to temperature changes[29]. While that work focused on growth rate, our model extends this perspective to enzyme-specific responses under nutrient limitation.

Combining both insights suggests that growth responses depend jointly on nutrient availability and temperature. Our framework predicts non-trivial, gene-specific changes in both the metabolome and proteome under combined perturbations. Thus, even though temperature changes are often assumed to induce limited proteome reallocation, substantial reorganization may occur under concurrent nutrient limitation.

References

1. Bremer H, Dennis PP. Modulation of Chemical Composition and Other Parameters of the Cell at Different Exponential Growth Rates. EcoSal Plus. 2008 1;3.
2. Jun S, Si F, Pugatch R, Scott M. Fundamental principles in bacterial physiology—history, recent progress, and the future with focus on cell size control: a review. Reports on Progress in Physics. 2018 5;81:056601.
3. Westerhoff HV, Palsson B. The evolution of molecular biology into systems biology. Nature Biotechnology. 2004 10;22:1249-52.
4. Raman K, Chandra N. Flux balance analysis of biological systems: applications and challenges. Briefings in Bioinformatics. 2009 7;10:435-49.
5. Molenaar D, van Berlo R, de Ridder D, Teusink B. Shifts in growth strategies reflect tradeoffs in cellular economics. Molecular Systems Biology. 2009 1;5.
6. Dam S, Pagès JM, Masi M. Stress responses, outer membrane permeability control and antimicrobial resistance in Enterobacteriaceae. Microbiology. 2018 3;164:260-7.
7. Gu C, Kim GB, Kim WJ, Kim HU, Lee SY. Current status and applications of genome-scale metabolic models. Genome Biology. 2019 12;20:121.
8. Orth JD, Thiele I, Palsson B. What is flux balance analysis? Nature Biotechnology. 2010 3;28:245-8.
9. Monk JM, Lloyd CJ, Brunk E, Mih N, Sastry A, King Z, et al. iML1515, a knowledgebase that computes Escherichia coli traits. Nature Biotechnology. 2017 10;35:904-8.
10. Hui S, Silverman JM, Chen SS, Erickson DW, Basan M, Wang J, et al. Quantitative proteomic analysis reveals a simple strategy of global resource allocation in bacteria. Molecular Systems Biology. 2015 2;11:784.
11. Peebo K, Valgepea K, Maser A, Nahku R, Adamberg K, Vilu R. Proteome reallocation in Escherichia coli with increasing specific growth rate. Molecular BioSystems. 2015 2;11:1184-93.
12. Schmidt A, Kochanowski K, Vedelaar S, Ahrné E, Volkmer B, Callipo L, et al. The quantitative and condition-dependent Escherichia coli proteome. Nature Biotechnology. 2016 1;34:104-10.
13. Erickson DW, Schink SJ, Patsalo V, Williamson JR, Gerland U, Hwa T. A global resource allocation strategy governs growth transition kinetics of Escherichia coli. Nature. 2017 11;551:119-23.
14. Mori M, Zhang Z, Banaei-Esfahani A, Lalanne J, Okano H, Collins BC, et al. From coarse to fine: the absolute Escherichia coli proteome under diverse growth conditions. Molecular Systems Biology. 2021 5;17.
15. Gupta M, Johnson ANT, Cruz ER, Costa EJ, Guest RL, Li SHJ, et al. Global protein turnover quantification in Escherichia coli reveals cytoplasmic recycling under nitrogen limitation. Nature Communications. 2024 7;15:5890.

16. Bennett BD, Kimball EH, Gao M, Osterhout R, Dien SJV, Rabinowitz JD. Absolute metabolite concentrations and implied enzyme active site occupancy in Escherichia coli. Nature Chemical Biology. 2009 8;5:593-9.

17. Taymaz-Nikerel H, Mey MD, Baart G, Maertens J, Heijnen JJ, van Gulik W. Changes in substrate availability in Escherichia coli lead to rapid metabolite, flux and growth rate responses. Metabolic Engineering. 2013 3;16:115-29.

18. Link H, Fuhrer T, Gerosa L, Zamboni N, Sauer U. Real-time metabolome profiling of the metabolic switch between starvation and growth. Nature Methods. 2015 11;12:1091-7.

19. Radoš D, Donati S, Lempp M, Rapp J, Link H. Homeostasis of the biosynthetic E. coli metabolome. iScience. 2022 7;25:104503.

20. Maaløe O. In: Regulation of the Protein-Synthesizing Machinery—Ribosomes, tRNA, Factors, and So On. Springer US; 1979. p. 487-542.

21. Scott M, Gunderson CW, Mateescu EM, Zhang Z, Hwa T. Interdependence of Cell Growth and Gene Expression: Origins and Consequences. Science. 2010 11;330:1099-102.

22. O'Brien EJ, Lerman JA, Chang RL, Hyduke DR, Palsson B. Genome-scale models of metabolism and gene expression extend and refine growth phenotype prediction. Molecular Systems Biology. 2013 1;9.

23. Bordbar A, Monk JM, King ZA, Palsson B. Constraint-based models predict metabolic and associated cellular functions. Nature Reviews Genetics. 2014 2;15:107-20.

24. Wang H, Robinson JL, Kocabas P, Gustafsson J, Anton M, Cholley PE, et al. Genome-scale metabolic network reconstruction of model animals as a platform for translational research. Proceedings of the National Academy of Sciences. 2021 7;118.

25. Passi A, Tibocha-Bonilla JD, Kumar M, Tec-Campos D, Zengler K, Zuniga C. Genome-Scale Metabolic Modeling Enables In-Depth Understanding of Big Data. Metabolites. 2021 12;12:14.

26. Scott M, Klumpp S, Mateescu EM, Hwa T. Emergence of robust growth laws from optimal regulation of ribosome synthesis. Molecular Systems Biology. 2014 8;10:747.

27. Kacser H, Acerenza L. A universal method for achieving increases in metabolite production. European Journal of Biochemistry. 1993 9;216:361-7.

28. Li X, Walhout AJM, Yilmaz LS. Enhanced flux potential analysis links changes in enzyme expression to metabolic flux. Molecular Systems Biology. 2025 2;21:413-45.

29. Knapp BD, Willis L, Gonzalez C, Vashistha H, Jammal-Touma J, Tikhonov M, et al. Metabolic rearrangement enables adaptation of microbial growth rate to temperature shifts. Nature Microbiology. 2024 12;10:185-201.

30. Saa PA, Nielsen LK. Formulation, construction and analysis of kinetic models of metabolism: A review of modelling frameworks. Biotechnology Advances. 2017 12;35:981-1003.

31. Kerkhoven EJ. Advances in constraint-based models: methods for improved predictive power based on resource allocation constraints. Current Opinion in Microbiology. 2022 8;68:102168.

32. Bekiaris PS, Klamt S. COBRA-k: A powerful framework bridging constraint-based and kinetic metabolic modeling. Science Advances. 2026 1;12:3022.

33. Gutenkunst RN, Waterfall JJ, Casey FP, Brown KS, Myers CR, Sethna JP. Universally Sloppy Parameter Sensitivities in Systems Biology Models. PLoS Computational Biology. 2007 10;3:e189.

34. Terzer M, Maynard ND, Covert MW, Stelling J. Genome-scale metabolic networks. WIREs Systems Biology and Medicine. 2009 11;1:285-97.

35. Saa PA, Nielsen LK. Construction of feasible and accurate kinetic models of metabolism: A Bayesian approach. Scientific Reports. 2016 7;6:29635.

36. Choudhury S, Narayanan B, Moret M, Hatzimanikatis V, Miskovic L. Generative machine learning produces kinetic models that accurately characterize intracellular metabolic states. Nature Catalysis. 2024 8;7:1086-98.

37. Nilsson A, Nielsen J, Palsson B. Metabolic Models of Protein Allocation Call for the Kinetome. Cell Systems. 2017 12;5:538-41.

38. Bernstein DB, Sulheim S, Almaas E, Segrè D. Addressing uncertainty in genome-scale metabolic model reconstruction and analysis. Genome Biology. 2021 2;22:64.

39. Waley S. A note on the kinetics of multi-enzyme systems. Biochemical Journal. 1964 6;91:514-7.

40. Ehrenberg M, Kurland CG. Costs of accuracy determined by a maximal growth rate constraint. Quarterly Reviews of Biophysics. 1984 2;17:45-82.

41. Heinrich R, Klipp E. Control Analysis of Unbranched Enzymatic Chains in States of Maximal Activity. Journal of Theoretical Biology. 1996 10;182:243-52.

42. Klipp E, Heinrich R. Competition for enzymes in metabolic pathways: Implications for optimal distributions of enzyme concentrations and for the distribution of flux control. Biosystems. 1999 12;54:1-14.

43. Bruggeman FJ, Remeijer M, Droste M, Salinas L, Wortel M, Planqué R, et al. Whole-cell metabolic control analysis. Biosystems. 2023 12;234:105067.

44. Dourado H, Lercher MJ. An analytical theory of balanced cellular growth. Nature Communications. 2020 3;11:1226.

45. Dourado H, Liebermeister W, Ebenhöh O, Lercher MJ. Mathematical properties of optimal fluxes in cellular reaction networks at balanced growth. PLOS Computational Biology. 2023 6;19:e1011156.

46. Dourado H, Mori M, Hwa T, Lercher MJ. On the optimality of the enzyme–substrate relationship in bacteria. PLOS Biology. 2021 10;19:e3001416.

47. Noor E, Liebermeister W. Optimal enzyme profiles in unbranched metabolic pathways. Interface Focus. 2024 2;14.

48. Noor E, Flamholz A, Bar-Even A, Davidi D, Milo R, Liebermeister W. The Protein Cost of Metabolic Fluxes: Prediction from Enzymatic Rate Laws and Cost Minimization. PLOS Computational Biology. 2016 11;12:e1005167.

49. Kanehisa M. KEGG: Kyoto Encyclopedia of Genes and Genomes. Nucleic Acids Research. 2000 1;28:27-30.

50. Mori M, Hwa T, Martin OC, Martino AD, Marinari E. Constrained Allocation Flux Balance Analysis. PLOS Computational Biology. 2016 6;12:e1004913.

51. Bar-Even A, Noor E, Savir Y, Liebermeister W, Davidi D, Tawfik DS, et al. The Moderately Efficient Enzyme: Evolutionary and Physicochemical Trends Shaping Enzyme Parameters. Biochemistry. 2011 5;50:4402-10.

52. Kubitschek HE, Baldwin WW, Schroeter SJ, Graetzer R. Independence of buoyant cell density and growth rate in Escherichia coli. Journal of Bacteriology. 1984 4;158:296-9.

53. Basan M, Zhu M, Dai X, Warren M, Sévin D, Wang Y, et al. Inflating bacterial cells by increased protein synthesis. Molecular Systems Biology. 2015 10;11:836.

54. Oldewurtel ER, Kitahara Y, van Teeffelen S. Robust surface-to-mass coupling and turgor-dependent cell width determine bacterial dry-mass density. Proceedings of the National Academy of Sciences. 2021 8;118.

55. Benyamini T, Folger O, Ruppin E, Shlomi T. Flux balance analysis accounting for metabolite dilution. Genome Biology. 2010 4;11:R43.

56. de Groot DH, Hulshof J, Teusink B, Bruggeman FJ, Planqué R. Elementary Growth Modes provide a molecular description of cellular self-fabrication. PLOS Computational Biology. 2020 1;16:e1007559.

57. Chure G, Cremer J. An optimal regulation of fluxes dictates microbial growth in and out of steady state. eLife. 2023 3;12.

58. Fishov I, Zaritsky A, Grover NB. On microbial states of growth. Molecular Microbiology. 1995 3;15:789-94.

59. Neidhardt FC, Ingraham JL, Schaechter M. Physiology of the Bacterial Cell. A Molecular Approach. Biochemical Education. 1992 4;20:124-5.

60. Lengeler JW, Drews G, Schlegel HG. In: Cellular and Subcellular Organization of Prokaryotes. Wiley; 1998. p. 19-46.

61. Milo R, Phillips R. Cell Biology by the Numbers. Garland Science; 2015.

62. Scott M, Hwa T. Bacterial growth laws and their applications. Current Opinion in Biotechnology. 2011 8;22:559-65.

63. Boer VM, Crutchfield CA, Bradley PH, Botstein D, Rabinowitz JD. Growth-limiting Intracellular Metabolites in Yeast Growing under Diverse Nutrient Limitations. Molecular Biology of the Cell. 2010 1;21:198-211.

64. Xia J, Sánchez BJ, Chen Y, Campbell K, Kasvandik S, Nielsen J. Proteome allocations change linearly with the specific growth rate of Saccharomyces cerevisiae under glucose limitation. Nature Communications. 2022 5;13:2819.

65. Cho BK, Barrett CL, Knight EM, Park YS, Palsson B. Genome-scale reconstruction of the Lrp regulatory network in Escherichia coli. Proceedings of the National Academy of Sciences of the United States of America. 2008 12;105:19462-7.

66. Caldara M, Charlier D, Cunin R. The arginine regulon of Escherichia coli: whole-system transcriptome analysis discovers new genes and provides an integrated view of arginine regulation. Microbiology. 2006 11;152:3343-54.

67. Forchhammer J, Lindahl L. Growth rate of polypeptide chains as a function of the cell growth rate in a mutant of Escherichia coli 15. Journal of Molecular Biology. 1971 2;55:563-8.

68. Pavlov MY, Ehrenberg M. Rate of Translation of Natural mRNAs in an Optimizedin Vitro System. Archives of Biochemistry and Biophysics. 1996 4;328:9-16.

69. Planqué R, Hulshof J, Teusink B, Hendriks JC, Bruggeman FJ. Maintaining maximal metabolic flux by gene expression control. PLOS Computational Biology. 2018 9;14:e1006412.

70. Scott M, Hwa T. Shaping bacterial gene expression by physiological and proteome allocation constraints. Nature Reviews Microbiology. 2023 5;21:327-42.

71. Wortel MT, Noor E, Ferris M, Bruggeman FJ, Liebermeister W. Metabolic enzyme cost explains variable trade-offs between microbial growth rate and yield. PLOS Computational Biology. 2018 2;14:e1006010.

72. You C, Okano H, Hui S, Zhang Z, Kim M, Gunderson CW, et al. Coordination of bacterial proteome with metabolism by cyclic AMP signalling. Nature. 2013 8;500:301-6.

73. Hu XP, Schroeder S, Lercher MJ. Proteome efficiency of metabolic pathways in Escherichia coli increases along the nutrient flow. mSystems. 2023 10;8.

74. Flamholz A, Noor E, Bar-Even A, Liebermeister W, Milo R. Glycolytic strategy as a tradeoff between energy yield and protein cost. Proceedings of the National Academy of Sciences. 2013 6;110:10039-44.

75. Noor E, Bar-Even A, Flamholz A, Reznik E, Liebermeister W, Milo R. Pathway Thermodynamics Highlights Kinetic Obstacles in Central Metabolism. PLoS Computational Biology. 2014 2;10:e1003483.

76. Greulich P, Scott M, Evans MR, Allen RJ. Growth-dependent bacterial susceptibility to ribosome-targeting antibiotics. Molecular Systems Biology. 2015 3;11:796.

77. Pinheiro F, Warsi O, Andersson DI, Lässig M. Metabolic fitness landscapes predict the evolution of antibiotic resistance. Nature Ecology & Evolution. 2021 3;5:677-87.

78. Rodrigues JV, Bershtein S, Li A, Lozovsky ER, Hartl DL, Shakhnovich EI. Biophysical principles predict fitness landscapes of drug resistance. Proceedings of the National Academy of Sciences. 2016 3;113:E1470-8.

79. Angermayr SA, Pang TY, Chevereau G, Mitosch K, Lercher MJ, Bollenbach T. Growth-mediated negative feedback shapes quantitative antibiotic response. Molecular Systems Biology. 2022 9;18.

80. Goldberg AL, Dice JF. Intracellular Protein Degradation in Mammalian and Bacterial Cells. Annual Review of Biochemistry. 1974 6;43:835-69.

81. Towbin BD, Korem Y, Bren A, Doron S, Sorek R, Alon U. Optimality and sub-optimality in a bacterial growth law. Nature Communications. 2017 1;8:14123.

82. Koch AL, Wang CH. How close to the theoretical diffusion limit do bacterial uptake systems function? Archives of Microbiology. 1982 2;131:36-42.

83. Akaike H. A New Look at the Statistical Model Identification. IEEE Transactions on Automatic Control. 1974;19:716-23.

84. Gerland U, Hwa T. On the Selection and Evolution of Regulatory DNA Motifs. Journal of Molecular Evolution. 2002 10;55:386-400.

85. Berg J, Willmann S, Lässig M. Adaptive evolution of transcription factor binding sites. BMC Evolutionary Biology. 2004 10;4:42.

86. Mustonen V, Kinney J, Callan CG, Lässig M. Energy-dependent fitness: A quantitative model for the evolution of yeast transcription factor binding sites. Proceedings of the National Academy of Sciences. 2008 8;105:12376-81.

87. Manhart M, Morozov AV. Protein folding and binding can emerge as evolutionary spandrels through structural coupling. Proceedings of the National Academy of Sciences. 2015 2;112:1797-802.

88. Held T, Klemmer D, Lässig M. Survival of the simplest in microbial evolution. Nature Communications. 2019 6;10:2472.

89. Labourel F, Rajon E. Resource Uptake and the Evolution of Moderately Efficient Enzymes. Molecular Biology and Evolution. 2021 8;38:3938-52.

90. Cornish-Bowden A. Fundamentals of Enzyme Kinetics. Wiley-Blackwell; 2012.

91. Mori M, Marinari E, Martino AD. A yield-cost tradeoff governs Escherichia coli's decision between fermentation and respiration in carbon-limited growth. npj Systems Biology and Applications. 2019 5;5:16.

92. Park JO, Rubin SA, Xu YF, Amador-Noguez D, Fan J, Shlomi T, et al. Metabolite concentrations, fluxes and free energies imply efficient enzyme usage. Nature Chemical Biology. 2016 7;12:482-9.

93. Bateman A, Martin MJ, Orchard S, Magrane M, Adesina A, Ahmad S, et al. UniProt: the Universal Protein Knowledgebase in 2025. Nucleic Acids Research. 2025 1;53:D609-17.

94. Alon U. Network motifs: theory and experimental approaches. Nature Reviews Genetics. 2007 6;8:450-61.

95. Goyal S, Yuan J, Chen T, Rabinowitz JD, Wingreen NS. Achieving Optimal Growth through Product Feedback Inhibition in Metabolism. PLoS Computational Biology. 2010 6;6:e1000802.

96. Kochanowski K, Sauer U, Chubukov V. Somewhat in control — the role of transcription in regulating microbial metabolic fluxes. Current Opinion in Biotechnology. 2013 12;24:987-93.

97. Kochanowski K, Gerosa L, Brunner SF, Christodoulou D, Nikolaev YV, Sauer U. Few regulatory metabolites coordinate expression of central metabolic genes in Escherichia coli. Molecular Systems Biology. 2017 1;13.

98. Heinrich R, Schuster S, Holzhütter H. Mathematical analysis of enzymic reaction systems using optimization principles. European Journal of Biochemistry. 1991 10;201:1-21.

99. Pang TY, Lercher MJ. Optimal density of bacterial cells. PLOS Computational Biology. 2023 6;19:e1011177.

100. Edwards JS, Palsson B. Systems Properties of the Haemophilus influenzae Rd Metabolic Genotype. Journal of Biological Chemistry. 1999 6;274:17410-6.

101. Heinrich R, Rapoport SM, Rapoport TA. In: Metabolic regulation and mathematical models. Elsevier; 1978. p. 1-82.

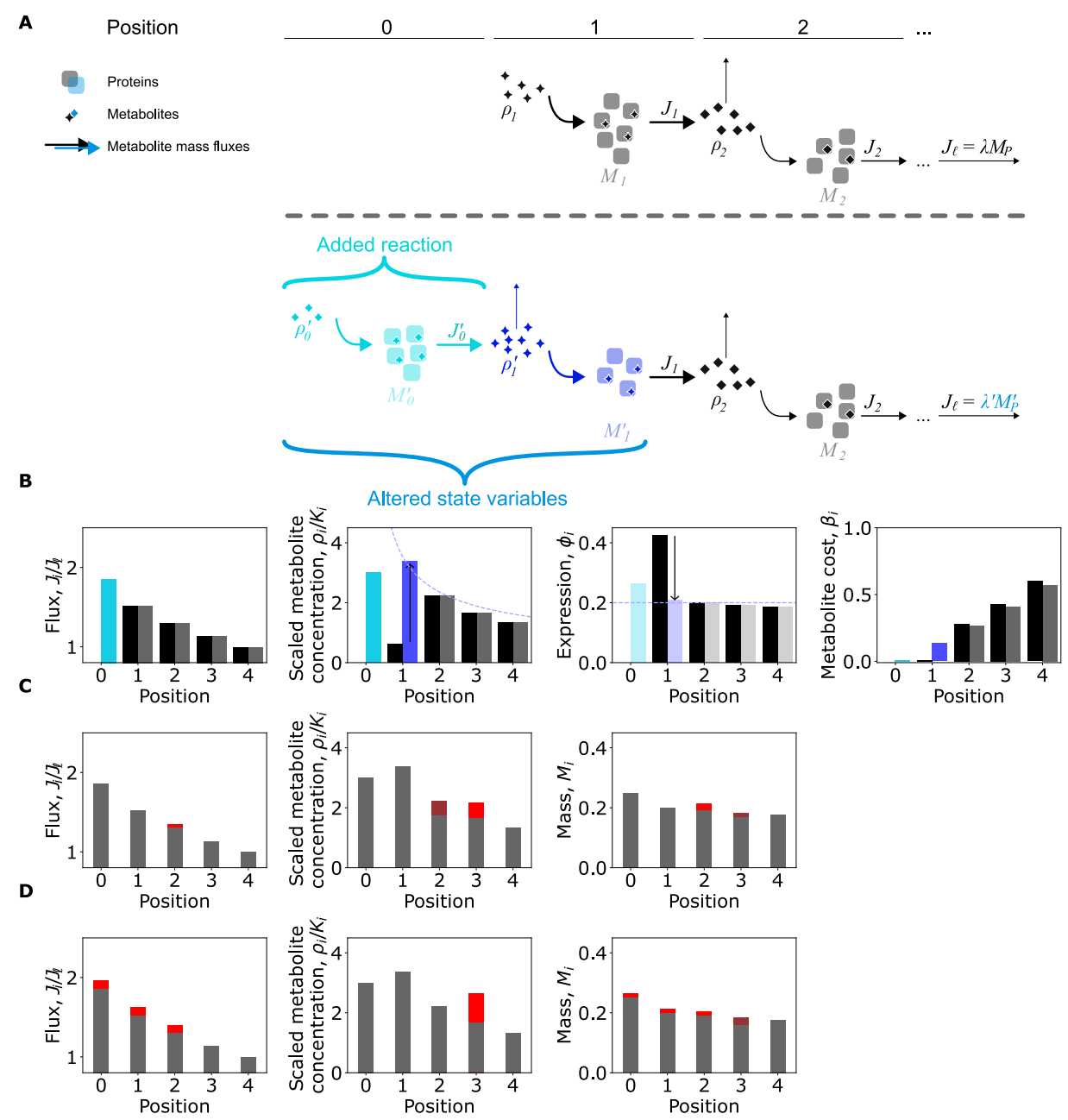

Figure A1. Optimal metabolic states can be constructed iteratively.
(A) For a two-enzyme chain, the optimal metabolic state in a given nutrient environment ρ_1 can be determined analytically[57]. Here we construct optimal metabolic states of longer chains, by iteratively adding an enzyme to the chain that produces the previous environmental nutrient.
Suppose all growth-optimal fluxes J of a chain of length ℓ are known. We show (Appendix) that a new growth-optimal set of fluxes can be constructed by adding a single new flux J'_0 to the chain and finding a suitable concentration of the new environmental nutrient ρ'_0. Such an extension of the chain defines a protein mass of the newly added enzyme M'_1, and affects the metabolite mass concentration of the previous environmental nutrient ρ'_1, as well as the required mass of the consuming enzyme M'_1, but does not affect further downstream metabolite mass concentrations or enzyme masses, making the problem analytically tractable.
(B) The optimal metabolic state of an exemplary short chain (left, black) and the optimal state of the corresponding elongated chain (right, shaded) are shown next to each other for comparison. For simplicity, we illustrate a cascade with equal substrate affinities $K_i = 0.1$ and catalytic constants $\kappa_i = 1\ \mathrm{h}^{-1}$ for all enzymes. A system of five equations can be solved for the five modified or new state variables J'_0, M'_0, ρ'_0, M'_1, and ρ'_1. All other fluxes, saturations, and masses are conserved under this elongation. Instead of enzyme masses M_i, dimensionless enzyme mass fractions M_i/M_P are shown. Light gray bars represent unchanged enzyme masses before rescaling due to the change of $M_P \to M'_P$, caused by the added mass $M'_0 + M'_1 - M_1$. Arrows indicate changes in the metabolite mass concentration ρ'_1 and enzyme mass M'_1 caused by adding a metabolite-producing reaction with index $i = 0$. The resulting patterns in fluxes, saturation, and expression can be understood through the cost of metabolite production β_i. The cost reflects how many proteome resources are needed to produce a given metabolite.
For physiological chain parameters, the cost of metabolite production is well approximated by the proteome fraction upstream of the reaction of interest. It increases as a function of position. Thus, the optimal metabolite concentration decreases with position. The resulting gradient is well-approximated by $(Ki)^{-1/2}$ (curved dashed line). At the same time, dilution of these metabolites induces gradients in mass fluxes. Both gradients in flux and enzyme saturation together result in a very weak gradient in enzyme mass fractions along the metabolic chain. Equal allocation to all five enzymes is shown as a horizontal dashed line for comparison.
(C, D) Changes in metabolic state variables for the perturbation of a growth-optimal state in a single flux or metabolite concentration, as discussed in the main text. (C) Upon a variation that increases flux J_2 in comparison to the growth-optimal state, the metabolite concentration ρ_3 increases, whereas ρ_2 decreases. At the same time, the enzyme masses required for these fluxes and metabolite concentrations in steady state, change for enzymes 2 and 3. In the optimal state, the total proteome mass must remain unchanged for a small variation in any single flux. (D) A concerted variation of all fluxes upstream of metabolite 3 leads to an isolated change in ρ_3. All enzyme masses upstream increase, whereas the mass M_3 is reduced. In the optimal state, the total proteome mass must remain unchanged for a small variation in a single metabolite concentration. In all panels, increases are shown in bright red, whereas decreases are shown in dark red.

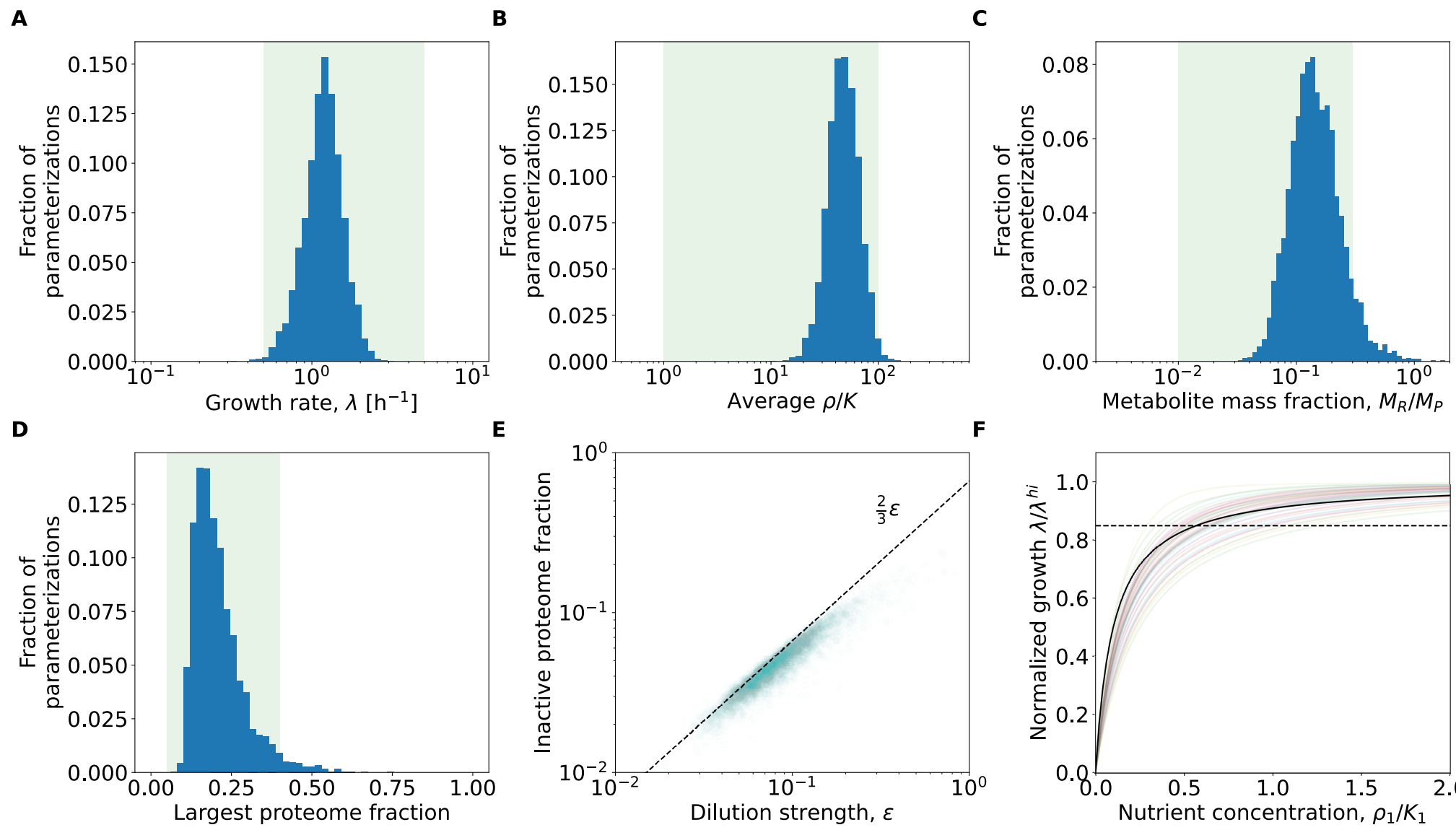

Figure A2. Model cells exhibit physiological properties.
An ensemble of 5000 linear chains of enzymes was parameterized by drawing individual enzyme affinities and catalytic constants from log-normal distributions with physiological mean and variance informed by measurements of Bar-Even et al.[51] (see A1 Table). The environmental nutrient concentration was set to $\rho_0 = 1000K_0$ to reflect a nutrient-rich environment.
(A) Cells parameterized in this manner exhibit physiological growth rates between $0.5\ \mathrm{h}^{-1}$ and $5\ \mathrm{h}^{-1}$.
(B) The geometric mean of scaled intracellular metabolite concentrations (ρ_i/K_i for $i = 2, \ \ldots, \ \ell$) in physiologically parameterized model cells matches physiological estimates: enzymes are typically operating above their K, but not at exceedingly high substrate concentrations. Thus, values between 1 and 100 are deemed physiological.
(C) The cumulative metabolite mass fraction of all metabolite species in a cell. Typically, metabolites contribute on the order of $10\,\%$ of the proteome mass to the total dry mass in a cell (from BNID: 101436). A small fraction of physiologically parameterized cascades exhibit unphysiologically large metabolite mass fractions, exceeding the single percent range. We hypothesize that this result, as well as a relatively high average saturation of enzymes in our model, may stem from neglecting additional growth costs associated with metabolites in a real cell.
(D) In a typical bacterial cell, the largest single proteome fraction is given by the ribosome and constitutes about $30\,\%$ of the cellular proteome. A small fraction of physiologically parameterized cascades exhibit unphysiologically large largest proteome mass fractions.
(E) Inactive proteome fraction predicted from the simplified resource allocation pattern (**76**) and obtained from optimal balanced growth states in physiologically parameterized cascades in the nutrient-rich regime.
(F) Predicted, representative nutrient dose-response functions for physiologically parameterized chains with a regulatory constraint on nutrient uptake limiting uptake overexpression to 5-fold (colored thin lines), are well approximated by a Monod-function with $K_{\mathrm{Monod}} = K_0/10$ (black solid line). The nutrient-rich regime is separated from the nutrient-poor regime by a growth reduction of $\phi_0^{\max} + \epsilon$ (on average at the black dashed line), which typically corresponds to $\rho_0 \approx K_0$.

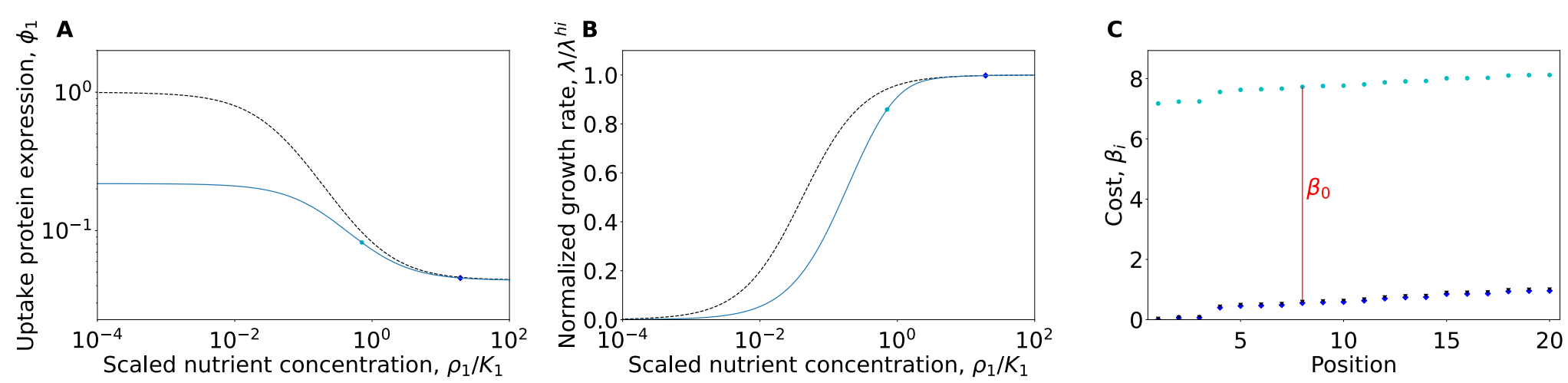

Figure A3. A nutrient uptake constraint increases the costs of metabolite production.
(A) Unconstrained optimal resource allocation leads to non-physiological overexpression of uptake proteins under nutrient limitation (black, dashed). This ultimately leads to a cell consisting solely of the nutrient-uptake enzyme. In contrast, we constrain uptake protein expression (blue) at low nutrient concentrations. The constraint was chosen such that it mimics unconstrained optimal resource allocation at high growth rates and exhibits the same Hill-like shape (Appendix).
(B) Growth is reduced in a Monod-like fashion in response to nutrient limitation for both unconstrained (black) and constrained (blue) overexpression of the uptake protein, with a Monod-constant on the order of the nutrient affinity of the uptake protein. The Monod-like growth response results mainly from the Michaelis-Menten kinetics of the nutrient uptake reaction but is modulated by the uptake expression change, as well as the increasing overall efficiency of nutrient conversion under nutrient limitation.
(C) When uptake expression is not constrained, the cost of metabolite production $\beta_i^\star$ increases several-fold along the metabolic chain (dark blue). It is closely approximated by the cumulative upstream proteome fraction. When the nutrient uptake is reduced below its optimal level, all costs increase (cyan dots). The additional cost is the same for all enzymes ($\beta_0^\star$). While the cost of metabolite production still increases monotonously, relative changes are reduced. When accounting for differences in c, this monotonous increase in cost along the chain leads to a monotonous gradient in metabolite saturations along the chain. As relative gradients in cost flatten upon nutrient limitation, concentration gradients flatten. This leads to a position-dependent fold change in metabolite concentration upon nutrient limitation.
All panels illustrate a single, representative, physiological parameterization of a heterogeneous chain of length $\ell = 20$.

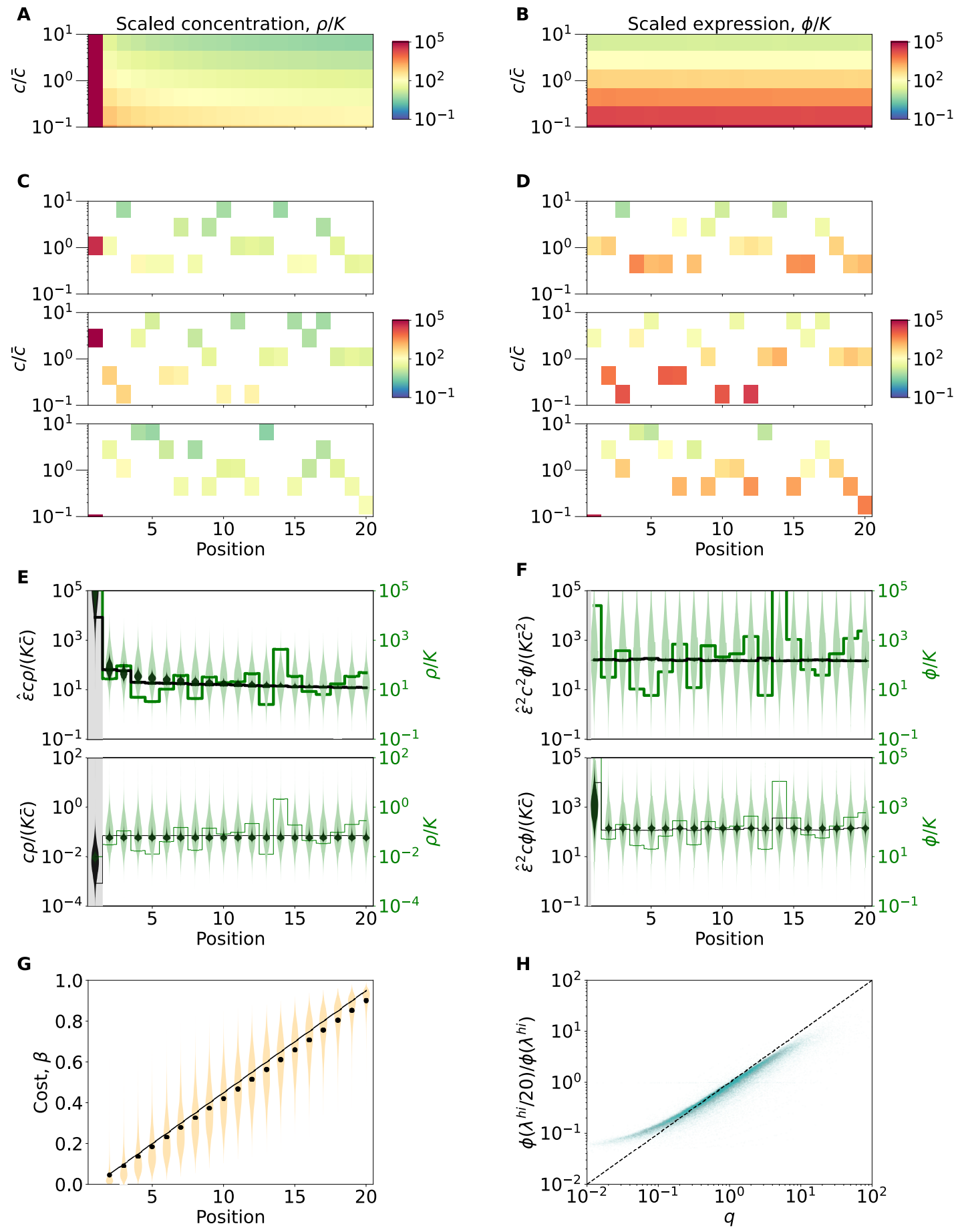

Figure A4. Position and biochemical quality-based predictions are accurate for individual simulated enzyme-substrate pairs.
(A, B) Analytical maps of optimal scaled metabolite concentrations and expression levels. (C, D) Corresponding maps of optimal scaled metabolite concentrations and expression levels for three individual model parameterizations. (E, F) To quantify the residual variability, unexplained by position and quality, we rescale growth-optimal concentrations and expression levels according to our theory by c. The normalization of $\bar{c}$ ensures that the mean value of all $c_i/\bar{c}$ is one - this enables a direct comparison of unscaled patterns (green) and scaled patterns (black). Both are shown for a single parameterization of the model cell (shown in the third line of (C, D), lines). Distributions of scaled metabolite concentrations and expression levels before and after rescaling by $c/\bar{c}$ and ϵ in nutrient-rich and poor environments from the ensemble of physiologically parameterized chains are shown as violins. To conserve the mean of each distribution and allow for a direct comparison of both scaled and unscaled variables, $\hat{\epsilon} \equiv \epsilon/\langle\epsilon\rangle$ was used for rescaling. Our theory reduces the squared residuals per enzyme of the $\log$ concentrations in the nutrient-rich regime by a factor 40, in the nutrient-poor regime by a factor of 6, and the squared residuals of the $\log$ expression levels by 50- and 5-fold respectively. (G) Distributions of metabolite production costs in an ensemble of physiologically parameterized chains as a function of enzyme position. The solid line corresponds to the estimate of $\beta_i^\star = (i-1)/\ell$. Black dots denote the means of the shown distributions. (H) Correlation between enzyme quality and protein growth response upon severe nutrient limitation for all individual enzymes of the ensemble (excluding nutrient uptake enzymes). Model parameters as in A2 Fig.
