# Supplementary material for: Enzyme kinetics shapes the growth response of metabolic networks
Source: PLoS Comput Biol. 2026 Aug 19;22(8):e1014642. doi: 10.1371/journal.pcbi.1014642 (PMC13533438; doi:10.1371/journal.pcbi.1014642)
Supplement: Supplementary file 2 [file pcbi.1014642.s002.pdf]

Pathway	KEGG ID	Proteins	$\hat{q}_p$	$\hat{\Delta}_p$	y	z	AIC	Sector	Notes
Shikimate pw	M00022	aroB, aroD, aroC, aroL, aroK, aroH, aroA, aroF⋆, aroG†	0.18± 0.04	0.42	**0.46± 0.13**	**1.26±0.64**	-249	T (R)	
Glucuronate degradation	M00061	uxuB, uxuA, uxaC, kdgK, eda	1.71± 0.04	2.54		**1.04±0.19**	-327		
3-Hydroxypropionate bi-cycle	M00376	fumC⋆, sdhB, sdhA, sdhC, accB, accC, accD, accA	1.88± 0.04	1.78		**0.44±0.24**	-491	C (R)	
Tetrahydrofolate bs	M00126	folA, folB, folP, folC, folE	0.16± 0.03	0.26		**0.61±0.12**	-63		
Ornithine-ammonia cycle	M00978	argF, argI, carB, carA, argG, argH, glnA†	0.12± 0.04	0.31	**0.20± 0.04**	**0.73±0.43**	-141		
Pentose phosphate pw	M00004	talA⋆, rpiB, pgl, rpe, rpiA, zwf, gnd, tktB, talB, tktA, pgi	0.75± 0.02	0.57	**0.44± 0.11**	**0.32±0.07**	-470	C (R)	
Glycolysis	M00001	pykA, ytjC, glk, gpmA, pfkB, pfkA, pgk, tpiA, fbaA, gapA, pgi, eno, gpmM, pykF†, fbaB⋆	0.39± 0.03	0.36		**0.26±0.16**	-622	C (R)	
Lysine bs	M00016	dapF, dapA, lysC, lysA, argD, dapE, metL, asd, dapB, thrA, dapD	0.27± 0.03	0.23	**1.01± 0.56**	**0.44±0.16**	-137	T (R)	
Methionine bs	M00017	malY, metA, metC, metH, metB, lysC, metL, asd, thrA, metE†	-0.03± 0.02	0.21	-0.01± 0.41	**0.57±0.12**	-399	T (R)	b-
De novo pyrimidine bs	M00051	pyrB, pyrI, pyrC, pyrF, pyrD, carB, pyrE, carA	0.40± 0.03	0.37	**0.31± 0.14**	0.21±0.28	-199		
Methionine salvage pw	M00034	tyrB, mtn, speE, metK, speD	0.44± 0.04	0.36		0.17±0.55	-77		
Citrate cycle	M00009	fumC⋆, frdB, frdA, acnA, fumB, fumA, acnB, sdhB, sdhD, sdhA, sucD, sucC, icd, sdhC, sucB, gltA, sucA, mdh, lpd	2.46± 0.08	0.89	0.19± 0.24	**0.15±0.03**	-295	C (R)	b+
Adenine ribonucleotide degradation	M00958	add⋆, ushA, umpG, yaiE, deoD, umpH	1.71± 0.03	1.06		0.21±0.16	-71		
Reductive pentose phosphate cycle	M00165	rpiB, prkB, rpe, rpiA, fbp, glpX, tktB, tktA, pgk, tpiA, fbaA, gapA	0.49± 0.02	0.21	0.07± 0.07	**0.26±0.04**	-618	C (R)	
Isoleucine bs	M00570	ilvB, ilvN, ilvH, ilvI, ilvA, ilvD, ilvE, ilvM, ilvC	0.70± 0.02	0.46		**0.20±0.13**	-228	T (R)	
Pyridoxal phosphate bs	M00124	pdxJ, pdxH, pdxB, epd, serC	0.45± 0.05	0.33		**0.41±0.11**	-39		
De novo purine bs	M00048	purC, purT, purL, purN, purD, purH, purM, purB, purK purE, purF	0.43± 0.03	0.25		-0.08±0.15	-27		
Molybdenum cofactor bs	M00880	moaB, moaE, mog, moaC, moeA	1.56± 0.06	1.10		-0.04±0.49	-29		
Photorespiration	M00532	lpd, glyA, gcvT, gcvP, gcvH, katE⋆	0.97± 0.02	0.42		-0.26±0.17	-495	C (R)	
Betaine degradation	M00975	ilvA, sdaA, glyA, tdcG, sdaB	0.60± 0.03	0.16	-0.01± 0.13	-0.09±0.07	-15		
Pyrimidine deoxyribonucleotide bs	M00938	ndk, nrdE, nrdB, nrdA, dut, tmk, thyA	2.32± 0.10	0.48		-0.17±0.13	-325		
UDP-MurNAc bs	M00995	murB, glmM, murA, glmS, glmU	0.60± 0.02	0.19		**0.24±0.16**	-13		
F-type ATPase	M00157	atpE, atpH, atpF, atpA, atpD, atpC, atpG	1.64± 0.05	0.75		-0.06±0.11	-15		c
Assimilatory sulfate reduction	M00176	cysH, cysC, cysI, cysN, cysJ, cysD	0.20± 0.01	0.06		0.26±0.19	2	(U)	
C5 isoprenoid bs	M00096	ispF, dxs, ispE, ispD, ispG, ispH	1.04± 0.02	0.37		0.19±0.16	-22		
Heme bs	M00121	hemD, hemC, hemB, hemN, hemG, hemE, hemL, gltX	0.83± 0.02	0.23		**0.26±0.05**	-91		
Riboflavin bs	M00125	yigB, ribD, ribF, ribC, ribE, ribB	0.83± 0.02	0.23		**0.26±0.12**	-3		
Tryptophan bs	M00023	trpA, trpB, trpC, trpD, trpE	0.25± 0.03	0.06		0.04±0.48	5	T (R)	o
Fatty acid bs	M00083	fabB, fabG, fabZ, fabF, fabI, fabA	0.72± 0.01	0.20		-0.05±0.24	-3		
KDO2-lipid A bs	M00060	lpxC, lpxB, lpxA, waaA, lpxD, lpxM	1.20± 0.04	0.24		-0.05±0.12	-9		
Ubiquinone bs	M00117	ubiD, ubiH, ubiF, ubiI, ubiE, ubiG	1.07± 0.02	0.29		**0.25±0.13**	-11		
Histidine bs	M00026	hisH, hisF, hisB, hisD, hisA, hisC, hisI, hisG	0.45± 0.05	0.13		**0.26±0.15**	6	T (R)	o
NADH:quinone oxidoreductase	M00144	nuoI, nuoJ, nuoF, nuoG, nuoC, nuoM, nuoB, nuoE, nuoH, nuoA, nuoL	2.39± 0.06	0.16		0.01±0.06	-1		c
Ornithine bs	M00028	argD, argB, argA, argE, argC	0.41± 0.02	0.03		-0.13±0.07	9	T (R)	
Nitrogen metabolism	map00910	can, gdhA, gltD, gltB, glnA†	0.29± 0.03	0.30				N (U)	
Carbon metabolism	map01200	acs, fumC⋆, talA⋆, glcB, pck, frdB, yqeF, frdA, acnA, maeB, fumB, fumA, acnB, sdhB, yeiG, ppsA, pykA, sdhD, rpiB, sdhA, sucD, sucC, icd, sdhC, prkB, pgl, sucB, rpe, gltA, sucA, mdh, rpiA, cysE, lpd, pta, ytjC, kdgK, maeA, accB, ilvA, ydbK, glk, fbp, accC, ackA, cysK, frmA, prs, zwf, accD, glpX, accA, gnd, aceF, sdaA, tktB, talB, gpmA, tktA, pfkB, eda, aceE, pfkA, glyA, pgk, tpiA, fbaA, serB, frmB, folD, gcvT, gcvP, gcvH, tdcG, sdaB, gapA, serC, pgi, ppc, metF, eno, edd, gpmM, serA†, pykF†, aceA⋆, aceB⋆, fadB⋆, katE⋆, fbaB⋆	1.31 ± 0.02	1.92				C (R)	
Biosynthesis of amino acids	map01230	talA⋆, acnA, acnB, pykA, malY, ilvB, rpiB, ilvN, proC, icd, alaA, aroB, leuB, rpe, gltA, ilvH, rpiA, cysE, dapF, aroD, ltaE, proA, tyrB, ytjC, ilvI, argF, leuA, metA, leuC, ilvA, argI, leuD, aroC, mtn, aspC, aroL, metC, cysK, prs, luxS, ilvD, dapA, cysM, sdaA, tktB, talB, proB, gpmA, tktA, metH, hisH, hisF, ilvE, pfkB, hisB, hisD, pfkA, metB, ilvM, asnB, glyA, hisA, hisC, lysC, pgk, lysA, tpiA, fbaA, serB, argD, pheA, ilvC, dapE, argB, hisI, asnA, argA, argE, tdcG, sdaB, aroK, argC, metL, gapA, hisG, serC, metK, trpA, trpB, trpC, trpD, asd, thrC, thrB, trpE, eno, dapB, argG, thrA, argH, gltD, dapD, aroH, gpmM, aroA, gltB, tyrA†, aroF⋆, serA†, aroG†, pykF†, metE†, glnA†, fbaB⋆	0.80± 0.01	0.76				T (R)	
Ribosome	map03010	rpmH, rplX, rplL, rpsR, rpsU, rpmB, rplW, rpsL, rpsJ, rplF, rpsQ, rpmF, rpsT, rplY, rplI, rplJ, rpsF, rplE, rplV, rpmC, rpsI, rpsH, rplM, rpsK, rpsO, rplU, rpsE, rpsA, rplR, rpsC, rplD, rpsP, rplQ, rpsM, rpmA, rpsG, rpsD, rpmD, rplP, rplN, rpsB, rplK, rplA, rplC, rpmG, rpsS, rplB, rplT, rplS, rpsN, rplO	0.37± 0.01	0.08				T (R)	c

Table S1. Empirical data of pathways and enzymes. Curated pathways (top) and sectors (bottom) of *E. coli* used in this study are listed by decreasing variance in log growth response ($\log \hat{q}$). From left to right: (1) Pathway name. (2) KEGG ID. (3) Enzymes measured in all growth conditions [14], ordered by decreasing $\hat{q}$ (top to bottom in Figs 1, 3, S1, and S6) (4) Mass-weighted average expression growth response, $\hat{q}_p$ ($\pm$ standard error). (5) Standard deviation $\hat{\Delta}_p$ of intra-path response coefficients $\hat{q}$. (6) Fit exponent y ($\pm$ standard error) of the empirical correlation between growth response and saturation at high growth (Figs 3 and S6), cf. equation (**6**). Power-laws are marked in bold face and shown in Fig 3 if the exponent differs by > 1.5 standard deviations from zero. (7) Fit exponent z ($\pm$ standard error) of the empirical correlation between growth response and expression at high growth (Figs 3 and S6), cf. equation (**7**). Power-laws are marked in bold face and shown in Fig 3 if exponents differ by more than 1.5 standard deviations from zero. Outlier proteins excluded from fits are marked by a star (Methods). (8) Akaike Information Criterion [83] of the comparison between a joint linear response model for the entire pathway and an enzyme-specific linear response model. Negative values indicate that the data is best explained by an enzyme-specific response model, positive values indicate a homogeneous pathway response. (9) Sector association based on metabolic module association with Nitrogen metabolism (N), Terminal anabolism (T) or Carbon metabolism (C) according to the KEGG classification [49]. Terminal anabolism and carbon metabolism are restricted (R). Nitrogen metabolism and Sulfur metabolism are unrestricted (U). (10) Notes indicate pathways in specific classes (see main text): [**c**] (protein complex), [**o**] (operon), [**b**−] (kinetic parameter bias towards specificity-optimization), [**b**+] (kinetic parameter bias towards rate-optimization). Abbreviations: pathway (pw), biosynthesis (bs).
