# Supplementary material for: Enzyme kinetics shapes the growth response of metabolic networks
Source: PLoS Comput Biol. 2026 Aug 19;22(8):e1014642. doi: 10.1371/journal.pcbi.1014642 (PMC13533438; doi:10.1371/journal.pcbi.1014642)
Supplement: Supplementary file 3 [file pcbi.1014642.s003.pdf]

Symbol	Name
V	Cell volume
J	Mass flux output of a reaction or pathway
R	Cellular mass of a metabolite type or group
M	Cellular mass of a metabolite type or group
$M_{P/R}$	Total cellular protein/metabolite mass
J_P	Total protein-biomass production flux
J_0	Nutrient-supply flux
$M_r(J_0)$	Total mass of enzymes sustaining the nutrient-supply flux J_0
κ_i	Mass-normalized catalytic constant of enzyme species i
K_i	Substrate binding constant of enzyme species i
λ	Cellular growth rate
$\lambda^{\max}$	Maximum growth rate in the absence of metabolite dilution
j	Protein-mass-normalized flux through a reaction or pathway
ϕ	Protein mass fraction of an enzyme or pathway
ρ	Total protein-mass-normalized concentration of a metabolite species
v	Flux per unit enzyme mass for a reaction or pathway
σ	Substrate saturation of an enzyme species
η	Metabolome-to-proteome mass ratio at high growth
ϵ	Dilution strength (deviation from production-consumption balance)
ℓ	Number of reactions in a metabolic pathway
$\cdot_{i,p,s}$	Reaction, pathway, or sector index
$\cdot_{\nu}$	Index of the nutrient-uptake reaction
$\cdot^{\text{hi,lo}}$	Value at high nutrient supply or extrapolated value at zero growth
$\cdot^{\star}$	Growth-optimal value of a variable
$\bar{\cdot}$	Average or stationary value of a variable, as specified in context
$\hat{\cdot}$	Empirically estimated value of a variable
q	Enzyme growth-response factor extrapolated to zero growth
c	Kinetic growth-response parameter of enzyme species
β_i	Metabolite cost associated with metabolite species i
β_0	Metabolite cost component generated by constrained nutrient-uptake
A_i	Amplitude factor describing pathway position and metabolite cost
B_p	Contribution of pathway p to biomass production
ξ	Restriction factor of a metabolic sector
$Q^{\,,\rho,v,j}(\lambda)$	Growth-response factor of protein, metabolite, efficiency or flux
Δ_p	Root-mean-square variation of growth response within pathway p
y	Power-law exponent relating growth response to substrate saturation
z	Power-law exponent relating growth response to high-growth expression
α	Correlation exponent between catalytic and substrate-binding constants
C	Carbon metabolism
N	Nitrogen- and sulfur-metabolism
T	Terminal amino-acid- and protein-synthesis
R	Nutrient-restricted metabolic sector
U	Unrestricted metabolic sector
t	Time
ρ_ν	Concentration of the growth-limiting nutrient
$\bar{\rho}_i$	Stationary regulated concentration of metabolite species i
ϕ_i^0	Basal expression level of enzyme species i
$\phi_i^{\max}$	Physiological upper bound on enzyme expression
$\bar{\phi}_i$	Stationary regulated expression level of enzyme species i
K_i^{reg}	Binding constant of the regulatory unit controlling enzyme i
a_i	Maximal regulatory change in the synthesis rate of enzyme species i
b_i	Synthesis rate of enzyme species i
$\mathcal{S}$	Metabolic state defined by metabolite and enzyme concentrations

Table S1. Table of variables and parameters. List of mathematical symbols, abbreviations, and notation together with their definitions.
